# Machine learning and automation methods for the segmentation, classification and quantification of testicular tissue sections

**DOI:** 10.1530/RAF-25-0210

**Published:** 2026-05-29

**Authors:** Adam J R Gadd, Iris Sanou, Eleanor Brain, Jill Davies, Adomas Liugaila, Kathleen Duffin, Agnes Stefansdottir, Rod T Mitchell

**Affiliations:** ^1^ Centre for Reproductive Health, Institute for Regeneration and Repair, The University of Edinburgh, Edinburgh, UK; ^2^ Reproductive Biology Laboratory, Centre for Reproductive Medicine, Amsterdam UMC, University of Amsterdam, Amsterdam, The Netherlands; ^3^ Amsterdam Reproduction & Development Research Institute, Amsterdam UMC, University of Amsterdam, Amsterdam, The Netherlands; ^4^ Royal Hospital for Children & Young People, Edinburgh, UK; ^5^ Oxford Cell and Tissue Biobank, John Radcliffe Hospital, Oxford University Hospitals NHS Trust, Oxford, UK; ^6^ Biomedical Sciences, Edinburgh Medical School, The University of Edinburgh, Edinburgh, UK

**Keywords:** human, testis, germ cells, machine learning, cell quantification

## Abstract

**Graphical Abstract:**

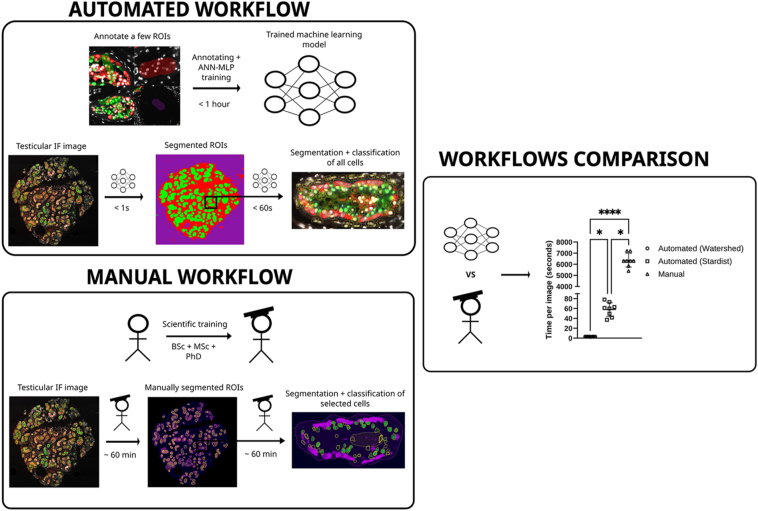

**Abstract:**

Analysis of immunofluorescent images can be time-consuming and observer-dependant. We aimed to develop a standardised method for analysing immunofluorescent images of human and mouse testicular tissues using an existing software package. We used QuPath to train and apply an artificial neural network with multilayer perception (ANN-MLP) on images. We were able to automate the segmentation of cells in regions of interest (ROIs) using both StarDist (a convolution neural network) and Watershed image transformation. Segmented cells were classified using QuPath object classification system, which was successfully applied to a range of mouse and human interstitial, Sertoli and spermatogonial germ cell markers. We found that manual counting and classification of cells decoupled the relationship between tubular area and a number of SOX9^+^ (*r*^2^ = 0.26, *P* = 0.35) and MAGE-A^+^ (*r*^2^ = 0.26, *P* = 0.35) cells. However, automating the segmentation of ROIs and cell classification with simple macros yielded results that maintained the correlation between tubular area and number of SOX9^+^ (*r*^2^ = 0.56, *P* = 0.03) and MAGE-A^+^ (*r*^2^ = 0.93, *P* = 0.002) cells. In addition, we were able to export data into R/RStudio allowing for the analysis of classification-specific parameters such as mitotic index and cellular organisation in different regions of the tubule. Importantly, the time taken per image using the automated method was significantly faster (6,247 vs three seconds; *P* < 0.001) at segmenting tubules and quantifying cells than previous manual annotation methods. We propose the use of this method for analysis and cell quantification in testicular tissues.

**Lay summary:**

Machine learning (ML) is a type of algorithm that forms part of artificial intelligence (AI). ML is able to learn, detect and predict sequences and structures such as words in sentences or objects in images. ML combined with the ability of modern computers to process large quantities of data and perform repetitive tasks in an automated way makes it an attractive research tool. In particular, the analysis of images taken from a microscope of patient or experimental samples is one area in which ML can excel. We found that open-source software containing ML could be trained on as few as six images. Once trained, the machine learning algorithm could analyse an image in approximately one minute. The same image would take a skilled researcher nearly two hours to analyse. In addition to speed, ML was able to do this more accurately and consistently as well as being automated by a simple piece of code.

## Introduction

Childhood cancer survival rates have increased to >80% for the following five years after diagnosis (Murphy *et al.* 2013). Long-term survivors face an increased risk of functional impairments, as well as secondary malignancies (Armstrong *et al.* 2009, Ward *et al.* 2014). Of the noted impairments, reduced fertility of the survivors (Chow *et al.* 2016, Skinner *et al.* 2017) has been attributed to the off-target effects of gonadotoxic cancer treatments (Anderson *et al.* 2015). In prepubertal boys, fertility preservation is limited to testicular tissue cryopreservation (Duffin *et al.* 2024) as undifferentiated spermatogonia, required for spermatogenesis, do not begin to differentiate into sperm until puberty (Mitchell *et al.* 2008, Ntemou *et al.* 2019). In the prepubertal testis, the germ cell population consists of undifferentiated spermatogonia, which include spermatogonial stem cells (SSCs) (Altman *et al.* 2014). Spermatogonia, including the SSC population, express melanoma antigen genes-A (MAGE-A), in contrast to the meiotic cells in the later stages of spermatogenesis (Valli-Pulaski *et al.* 2019, Guo *et al.* 2020). SSCs and undifferentiated spermatogonia are typically intermingled with Sertoli cells (SOX9^+^) within structures known as seminiferous tubules (Guo *et al.* 2020). Infertility can result from the loss of SSCs due to chemotherapy in childhood (Lopes *et al.* 2020).

To develop and understand treatments for fertility preservation, a range of histology-based workflows have been developed for the characterisation of seminiferous tubules and quantification of germ cells. These typically rely on intensive manual annotation of tubules, performing manual counts of cells or determining qualifying regions of interest (ROIs) by shape or other criteria (Medrano *et al.* 2021, Lahtinen *et al.* 2024). However, there is no consensus on the preferred methodology (Kearney *et al.* 2025). The advent of machine learning has been applied to the field of digital immunohistochemistry where whole slide images (WSIs) are captured at high magnification and deep learning models are trained to perform pathology analysis (Komura *et al*. 2025). This has allowed for the simplification and consistency of analysis by removing the human decision-making element, but these methods are still significantly hindered by the large dataset needed for deep learning training and the time-consuming manual annotation (Komura *et al*. 2025). To alleviate this bottleneck, large datasets of WSIs and corresponding ‘omics’ collections are publicly available; however, they typically focus on pathological analysis of cancer and may not be directly useful for analysis of other types of tissues, such as the human testis. In addition, there are a number of technological hurdles to overcome, such as handling large datasets or accounting for data variation from multiple sources or capture technologies when using these datasets for deep learning (Lonsdale *et al.* 2013, Weinstein *et al.* 2013).

Here, we found that models generated from the multilayer perception artificial neural network (MLP-ANN) featured within QuPath (Bankhead *et al.* 2017) were easily trainable using simple annotations from a small pool of images and able to detect and annotate specific ROIs in human testis tissues. QuPath’s features in synergistic combination with another pre-trained deep learning convolutional neural network (CNN) ‘StarDist’ for detecting star convex polygons (such as nuclei) (Schmidt *et al.* 2018, Weigert *et al.* 2020, Weigert & Schmidt 2022) or a traditional image processing method for discerning overlapping objects/pixel intensities called Watershed; we were able to generate ad-hoc machine learning workflows. These workflows could segment ROIs such as tubules and perform simple cell classification based on fluorescent immunohistochemistry markers. By automating this workflow using bespoke macros, analysis of an image typically takes as little as a few seconds to be processed depending on the cell segmentation method used. When applying automation to all images within an experiment, a single segmentation and classification system could be applied consistently, removing user bias from the quantification and significantly improving the speed of analysis. In addition, we found that this workflow could be applied across images from a range of developmental stages in testicular tissues, including mouse and human samples.

## Methods

### Automated image analysis

Human and mouse immunofluorescence (IF) images (protocol provided in supplementary information) were analysed using QuPath v0.6.0 (Bankhead *et al.* 2017) software in an automated and blinded manner using a Framework 13 laptop (Ryzen 7640U, 64 GB RAM). Images of tissue sections were produced by capturing multiple 20x images with 10% overlap regions using a 0.8 aperture Pan-Neo objective on a Zeiss Observer microscope with an automated stage. Single images were produced as CZI files by stitching overlapping regions using Zeiss Zen software. Images were imported into QuPath using the bio-formats importer without any transformation of the captured image.

**Figure 1:**
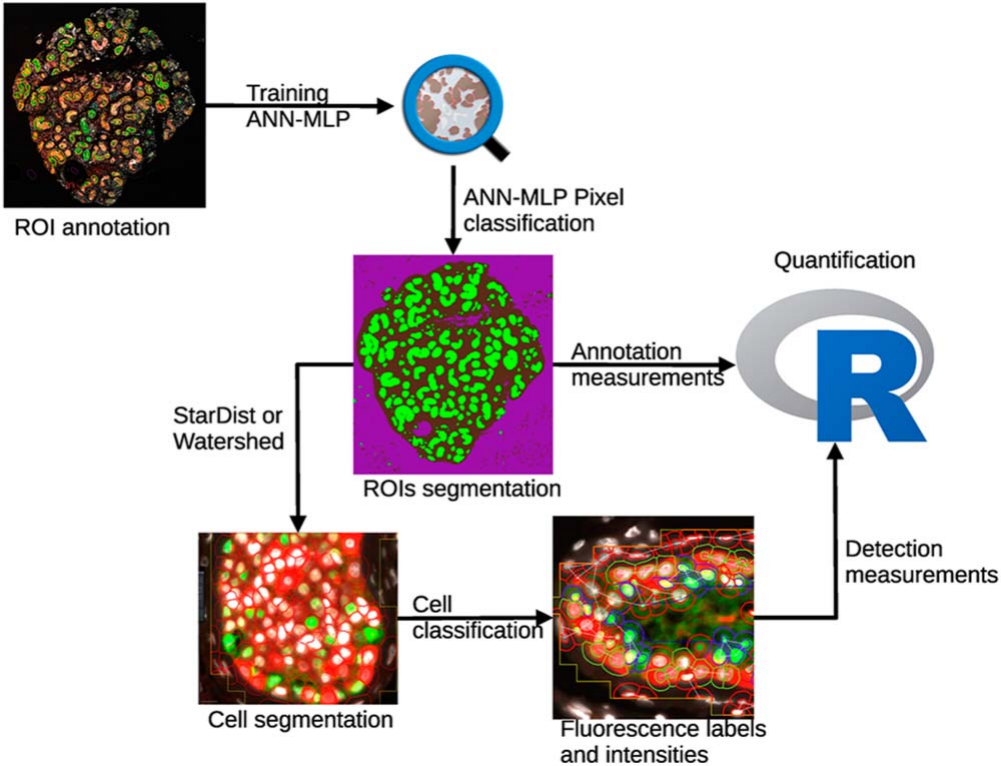
Workflow overview of automated testicular tissue analysis: immunofluorescent images were stitched together from tiled images captured at 20× magnification on an epifluorescent microscope. Small ROIs were annotated, typically three regions of each class per image. Annotations were then used to train QuPath’s ANN-MLP pixel classification system. Trained pixel classifiers were then used to segment areas of interest, specifically tubules. Annotation measurements were exported for quantification. Cells were then segmented in ROIs using either StarDist or Watershed to produce nuclei and cell boundaries. Cells were classified based on fluorescence intensity and location. Cell properties were exported as detection measurements for quantification. Data were then processed in RStudio and visualisation performed in RStudio or GraphPad Prism.

Initially, small ROIs were labelled as ‘tissue’, ‘tubule’ or ‘background’ and used to train an artificial neural network with multilayer perception (ANN-MLP) pixel classifier in QuPath. In general, three regions of each area of interest were labelled in each image (3× tubule, 3× interstitial tissues and 3× background) across six images (2× fully stained, 2× SOX9 antibody control, 2× MAGE-A antibody control). It was important to include antibody controls in the training images as it improved the consistency of tubule identification. Models were trained on all available fluorescent channels and on images spanning experimental groups (data presented consists of pre-culture controls for human samples and vehicle-treated mice) and antibody controls using the following selected features: Gaussian filter (intensity and colour), gradient magnitude (edges), structure tensor coherence (cell orientation) and Hessian determinant (blob-like structures). After training, images were then segmented using the trained pixel classification into ROIs consisting of ‘tubules’, ‘tissue’ and ‘background’ with the ‘minimum object size’ and ‘minimum hole size’ set to 400 and 200 μm^2^, respectively. Within ROIs such as tubules, cells were segmented using either a Watershed-based transformation or a CNN-based star-convex polygon approach ‘StarDist’ (Schmidt *et al.* 2018, Weigert *et al.* 2020, Weigert & Schmidt 2022) applied to the counterstain channel (DAPI or Hoechst). Once cell boundaries (nuclei and cytoplasm) had been established, cells were then classified by fluorescence location and fluorescence intensity using QuPath’s object classifier. Cells were categorised according to marker-specific characteristics such as nuclear intensity of SOX9 or cytoplasmic intensity of MVH. Cells not meeting classification criteria were designated as unclassified. Parameters for pixel and object classification remained consistent for each set of experiments. All MAGE-A and SOX9 images had the same pixel and object classification settings. Once classified, the total area of ROIs and the frequency of cell classes were measured allowing for cells to be calculated as follows: frequency (number)/area (mm^2^). Data were exported as either ‘annotation measurements’ or ‘detection measurements’ for further processing (outlined in Fig. 1). Additional complexity can be added to the ANN-MLP training annotations that can identify areas of tissue that are damaged, overexposed or out of focus. We found this was largely not necessary as the classifier was able to annotate intact sections of tubules around damaged areas and either any of the cellular classification tools in QuPath or data analysis could be used to highlight the identification of erroneous data.

### Manual image analysis

After acquisition, image file names were first randomised to prevent bias during analysis. File name randomisation was performed according to https://www.howtogeek.com/57661/stupid-geek-tricks-randomly-rename-every-file-in-a-directory/.

Images were loaded into QuPath; tissue fragments and the corresponding tubules were then identified and manually annotated using the wand and brush tool. Aggregates of cells present in each tubule were then identified and manually annotated based on the presence of pyknotic nuclei. MAGE-A^+^ cells were then manually counted using the point-to-annotation counting tool (physically clicking on the corresponding fluorescence associated with MAGE-A staining). SOX9^+^ cells were then identified using QuPath cell detection system on the SOX9 fluorescent channel based on the SOX9^+^ fluorescent area. Tissue sections were excluded if there were not both SOX9 and MAGE-A staining in tubules, or if the tissue was perceived to be mechanically damaged, or if the sensitivity of fluorescence staining was too high, i.e. settings were adjusted and gauged based on conformity to fully stained and antibody control staining. Once completed, cell counts, area of fluorescence and area of tissue/tubules were exported and processed using GraphPad Prism 10 software (USA).

### Data processing

Annotation measurements were exported from QuPath as CSVs with graphs and stats produced from GraphPad Prism 10 using either two-way ANOVA or non-parametric Mann–Whitney test with statistical significance set at *P*=<0.05.

Packages in R (dplyr (Wickham *et al.* 2025), readr (Wickham *et al.* 2024) and ggplot2 (Wickham 2016)) were installed via renv (Ushey & Wickham 2025). Detection measurements (consisting of cell-specific measurements) were imported into R via readr. Dataframes were produced from filtered raw data by dplyr, and graphs were generated using ggplot2.

## Results

### Training ANN-MLPs for segmenting ROIs in human testicular tissue samples

**Figure 2:**
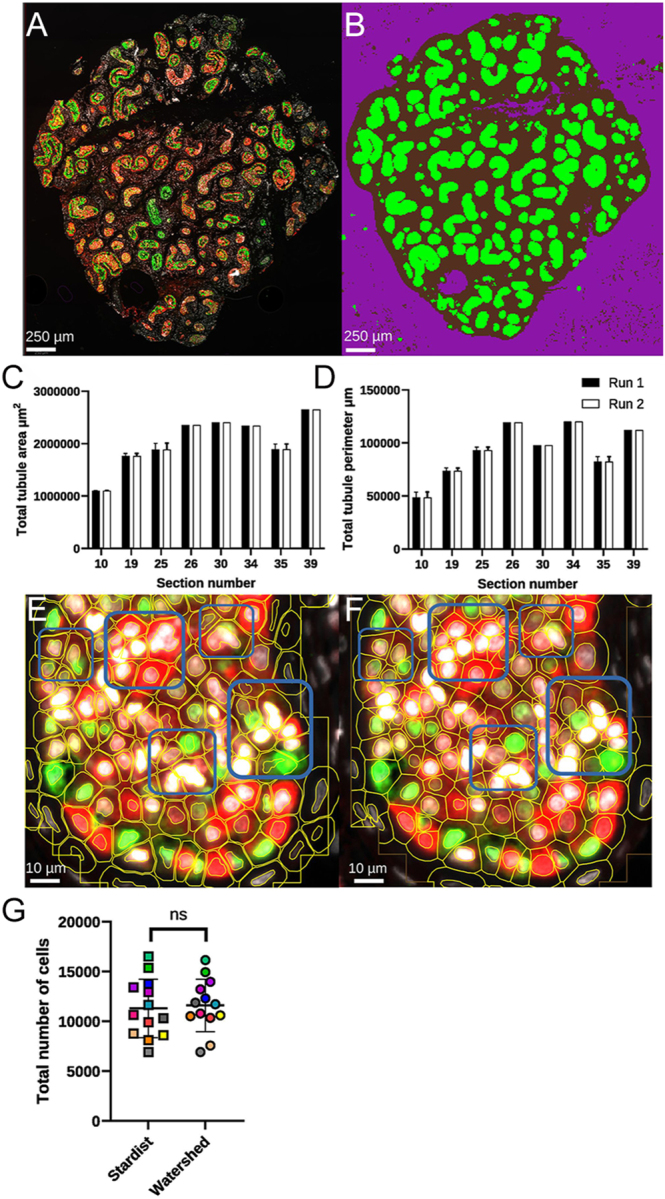
Segmentation of regions of interest (ROIs) such as tissue area, tubules and cells over multiple runs and histology sections. (A) Original immunofluorescent image prior to segmentation. (B) Prediction map of segmentation illustrating different ROIs: green – tubules, brown – tissue, purple – background, using QuPath’s ANN-MLP-based pixel classifier. (C) Total tubular area (μm^2^) and (D) total tubular perimeter (μm) corresponding to depth within the original tissue sample, each run represents the independent output from running ANN-MLP on the same sections. (E) Example of cell segmentation using a Watershed method. (F) Example of cell segmentation using StarDist. (G) Total number of cells detected by either Watershed- or StarDist-based cell segmentation; matching colours represent the same tissue section measured by either StarDist or Watershed. Data were collected from a total of 13 images from freshly fixed prepubertal testicular tissue. In panels (C, D and G), error bars represent standard deviation. Fluorescence staining in (A, E and F): red = MAGE-A; green = SOX9.

To better understand the role of automation for an image analysis workflow, an ANN-MLP pixel classifier was trained by annotating three small areas per image for each ROI (Fig. 1). Training was performed on selected images consisting of fully stained sections and antibody controls. Typically, two images per group were used to produce a classification system that was robustly and accurately able to segment regions of interest such as tubules in addition to other ROIs of the whole tissue section or background. Once trained the model was applied to the original image (Fig. 2A) and generated the corresponding prediction map (Fig. 2B). This illustrates the ability of an ANN-MLP within QuPath to segment regions satisfactorily. Importantly, it took less than one second to complete the annotation per image analysed. With an eye to accuracy and reproducibility, using the trained ANN-MLP pixel classifier on separate computers and different versions of QuPath (v0.5.1 vs v0.6.0), measurements were compared between runs from a range of sections. Run-to-run variation was found to be non-existent, and data were identical when comparing the area and perimeter of tubule segmentation (Fig. 2C and D, *P* => 0.99, two-way ANOVA). We also found that ANN-MLP could be easily adjusted based on the training annotations. In the examples here, the peritubular myoid (PTM) cells have been included in the training annotations and, therefore, images segmented with the trained ANN-MLPs will include these as part of the segmented tubule. We found that selecting features such as gradient magnitude (detecting edges) and structure tensor coherence (cell orientation) and including the PTM cells in the pixel classifier training gave very clear and clean segmentation of tubules. However, this could be optimised on other parameters or regions to define tubules if needed.

### Segmentation of cells using either conventional or pre-trained CNN models in human testicular tissue

The impact of using a conventional image transformation method for nuclei segmentation ‘Watershed’ (Fig. 2E) was compared to a convolutional neural network (CNN) model ‘StarDist’ that has been pre-trained for detecting star-convex polygons such as nuclei (Schmidt *et al.* 2018, Weigert *et al.* 2020, Weigert & Schmidt 2022) (Fig. 2F). Surprisingly, comparison between the two methods revealed no statistically significant differences (*P* = 0.34, Mann–Whitney test) in the number of cells detected by StarDist (11,308 cells) or Watershed (11,614 cells) across a range of sections (Fig. 2G). However, despite the lack of differences in the number of detections, we found the CNN method demonstrated higher segmentation quality and a greater ability to deconvolute multiple overlapping nuclei and align more consistently with perceived fluorescent staining (Fig. 2E and F).

### Impact of cell segmentation method on fluorescence-based cell classification in human testicular tissue

**Figure 3:**
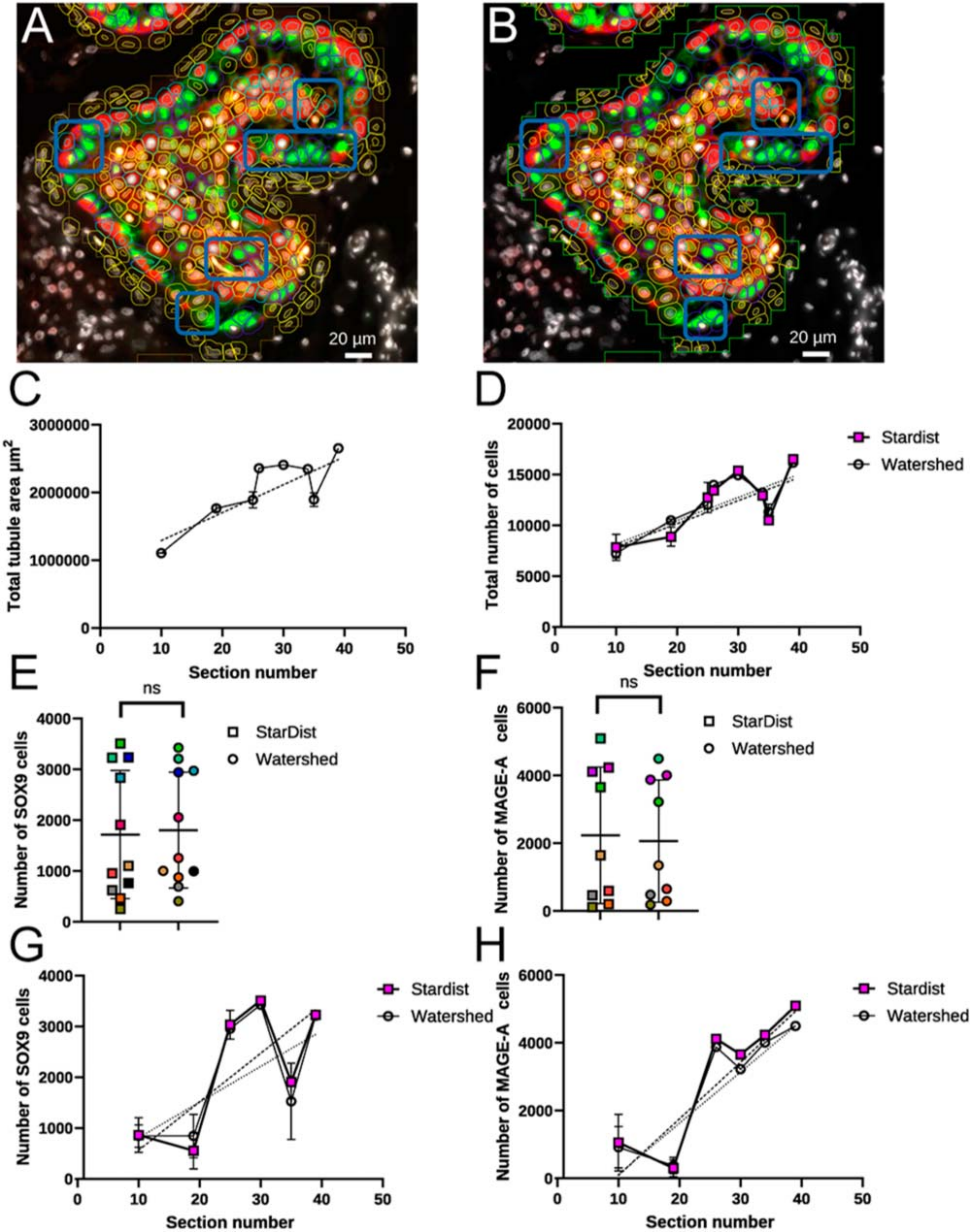
Fluorescence-based classification of cells segmented by either Watershed or StarDist methods. Cell classification: pale blue outline (red fluorescence) – MAGE-A cells, blue outline (green fluorescence) – SOX9 cells, unclassified cells – yellow outline. (A) Example of an immunofluorescent image after cell classification segmented using Watershed. (B) Example of an immunofluorescent image after cell classification segmented using StarDist. (C) Total tubular area (μm^2^) produced by MLP-ANN segmentation (*r*^2^ = 0.70, *P* = 0.0004). (D) Total number of cells segmented by either Watershed (*r*^2^ = 0.67, *P* = 0.0007) or StarDist (CNN machine learning model, *r*^2^ = 0.53, *P* = 0.0048). (E) Total number of cells classified as SOX9^+^ when using either Watershed or StarDist for segmentation. (F) Total number of cells classified as MAGE-A^+^ positive when using either Watershed or StarDist for segmentation. (G) Total number of cells classified as SOX9^+^ when using either Watershed (*r*^2^ = 0.37, *P* =) or StarDist (*r*^2^ = 0.51, *P* = 0.02) for segmentation over a range of tissue sections. (H) Total number of cells classified as MAGE-A^+^ when using either Watershed (*r*^2^ = 0.72, *P* = 0.0038) or StarDist (*r*^2^ = 0.70, *P* = 0.0047) for segmentation over a range of tissue sections. In panels (E and F), matching colours represent the same tissue section measured by either StarDist or Watershed. Measurements were taken from pooled data of 13 images representing 61,191 total cell detections. In panels (C, D, E, F, G, H), error bars represent standard deviation. In panels (G and H), the dotted lines represent linear regression.

The variation of segmentation quality between the conventional Watershed method (Fig. 3A) and the machine learning CNN model StarDist (Fig. 3B) was further investigated to understand the impact on fluorescence-based object classification.

The object classification system in QuPath can use both the location and intensity of fluorescence (among other parameters) to classify an object. As such, an object classification system based on fluorescence threshold and nuclear staining of MAGE-A and SOX9 was defined. This classification was applied after cells were segmented by both methods (Watershed and StarDist) to evaluate if the difference in cell boundaries calculated by each method impacted object classification (Fig. 3A and B, boxes illustrate differences). We found that as the section number increases (reflecting a greater depth within the tissue) the total tubular area segmented also generally increased due to the larger tissue area in the sections (*r*^2^ = 0.7, *P* =< 0.001, two-way ANOVA, Fig. 3C). As there was a larger tubular area segmented, this also in turn generally increased the number of cells detected overall by each method (*r*^2^ = 0.67 and 0.53 for Watershed and StarDist, respectively, *P* =< 0.001, two-way ANOVA, Fig. 3D). However, no difference was seen in the number of classifications when cells were pooled from all sections segmented by either Watershed or StarDist methods (Fig. 3E and F, *P* = 0.84 and *P* => 0.99 for SOX9 and MAGE-A, respectively, Mann–Whitney test). No difference was observed when comparing between methods at each specific section analysed (Fig. 3G and H *P* = 0.19 and 0.21 for SOX9 and MAGE-A, respectively, two-way ANOVA). This demonstrates that cell density, area of tubules and cell segmentation method have no impact on cell classification accuracy.

### Analysis of cellular organisation of MAGE-A and SOX9 cells within tubules in human testicular tissue

**Figure 4:**
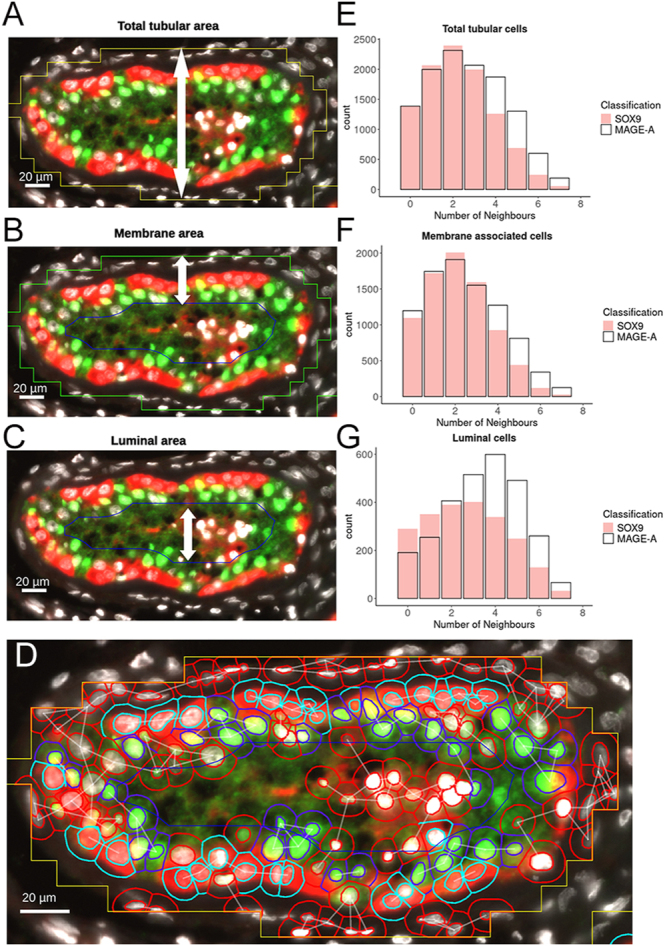
Clustering analysis of cell populations in tubules by comparing centroids of neighbouring cells in the same classification in different regions of the tubule. (A) Total tubular area. (B) Membrane area – cells located within this region are termed ‘membrane-associated cells’. (C) Luminal area – cells located within this area are termed ‘luminal cells’. (D) Example of tubule with cells classified by SOX9 (green fluorescence, dark blue outline), MAGE-A (red fluorescence, pale blue outline) and unclassified (red outline) using the ‘neighbour network’ of connected white lines. (E) Number of neighbours for SOX9 and MAGE-A cells located in the total tubular area. (F) Number of neighbours for SOX9 and MAGE-A membrane-associated cells. (G) Number of neighbours for SOX9 and MAGE-A luminal cells. Measurements were taken from pooled data of 13 images representing 61,191 total cell detections.

After establishing the consistency of automated segmentation and classification, we investigated distributions of cell populations in different regions of the tubule. The number of cells in proximity to another cell of the same classification is a parameter that will be referred to from here as the ‘number of neighbours of the same class’. To better understand the distribution of neighbouring cells in tubules, total tubule area was defined by the ANN-MLP (Fig. 4A). An additional segmentation boundary was then drawn within 35 μm of the original tubule boundary. This was typically two cell diameters and represented a simple method to separate a basement layer of cells from the rest of the tubule. Cells remaining between the original segmentation boundary and this new luminal boundary (blue line) were designated ‘membrane-associated’ (Fig. 4B). Cells located within this new segmentation area in the centre of the seminiferous tubules were designated ‘luminal’ cells (Fig. 4C). The centroid distances between cells of the same classification (MAGE-A^+^ or SOX9^+^ cells) were then calculated and a network of ‘neighbours’ (cells within 15 μm of each other) was produced (Fig. 4D). The frequency of neighbours for each class of cell either within total tubular area (Fig. 4E), the ‘membrane-associated’ (Fig. 4F) or ‘luminal’ cells was compared (Fig. 4G). We found that, when comparing the frequency of neighbouring cells of the same class (either MAGE-A or SOX9) in the total tubular area, the mode for both classes was two ‘neighbours’ per cell (Fig. 4E). This was also the case for membrane-associated cells (Fig. 4F). The distribution of SOX9^+^ cells remained consistent across all regions. However, when looking specifically at MAGE-A^+^ ‘luminal cells’, the mode was four neighbours per cell (Fig. 4G). This suggests that MAGE-A^+^ cells are more likely to cluster in larger groups 35 μm from the edge of the tubule.

### Mitotic index of MAGE-A^+^ and SOX9^+^ cells within tubules of human testicular tissue

**Figure 5:**
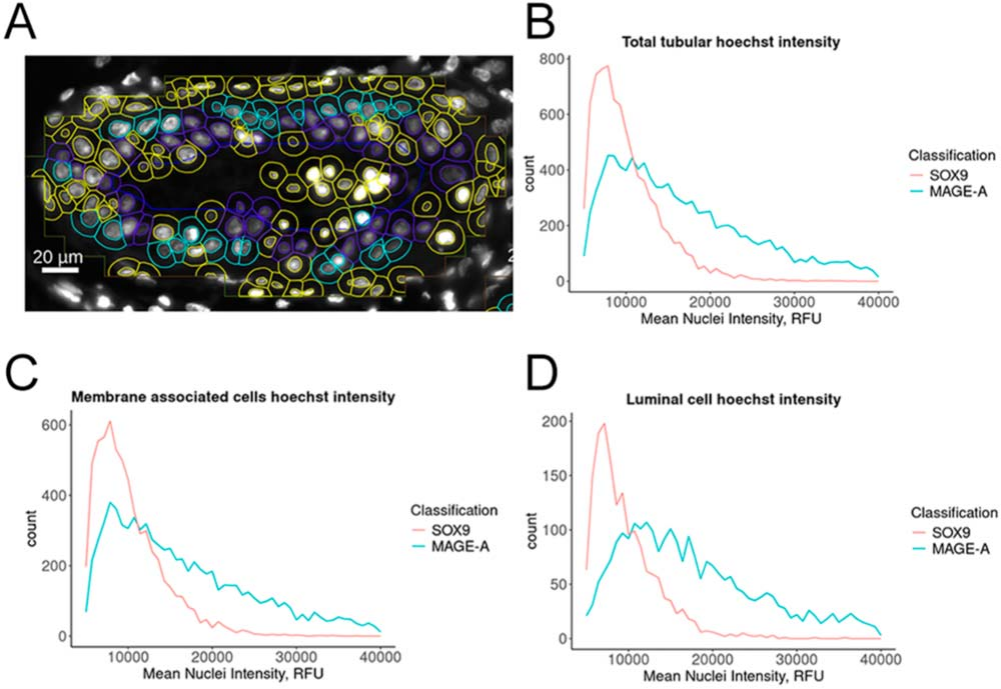
Hoechst intensity measurements in cells from different regions of the tubule. (A) Representative image of Hoechst intensity staining and cell classification in tubules (blue = SOX9, light blue = MAGE-A and yellow = unclassified) used to derive the mitotic index of cells. Hoechst intensity plots from SOX9 cells (dark blue outline) and MAGE-A cells (light blue outline) located in the (B) total tubular area. (C) Membrane area. (D) Luminal areas. Measurements were taken from pooled data of 13 images representing 61,191 total cell detections.

When cells are segmented and classified within QuPath, a large range of cell parameters are also calculated and available for analysis. This includes Hoechst intensities (Fig. 5A), which can be exported as a CSV and processed in RStudio to derive the mitotic index (the proportion of dividing cells). Hoechst 33342 binds to the minor groove in DNA where its conformation is stabilised and becomes fluorescent. This allows Hoechst (and other DNA-binding dyes) to quantify the amount of DNA present in a cell at representative parts of the cell cycle such as G0/G1 (2*n*), S (2-4*n*) and G2/M (4*n*) (Darzynkiewicz *et al.* 2017, Ligasová *et al.* 2023). We compared the profiles of MAGE-A cells to SOX9 cells using the latter as a proxy for the post-mitotic cell population (Fig. 5B). We found that the majority of SOX9 cells displayed a nuclear staining profile consistent with G1 phase of the cell cycle. This consisted of a peak intensity around 9,000 RFU, ranging from 6,000 to 14,000 RFU (Fig. 5B). We used the post-mitotic SOX9^+^ cells to create a threshold based on nuclear fluorescence intensity to identify cells as post-mitotic or mitotically active. Therefore, we set a threshold of 14,000 RFU and considered cells above this value to be mitotically active. While MAGE-A cells had a peak maximum of approximately 9,000 RFU, they had a broad range up to 25,000–30,000 RFU. This range is approximately twofold higher than the G1 peak, consistent with DNA doubling during cell division. Dataframes in R were then filtered based on cell location within the tubule and intensity profiles plotted for total tubular area (Fig. 5B), membrane-associated cells (Fig. 5C) and luminal cells (Fig. 5D), and mitotic index was calculated (Table 1).

**Table 1:** Mitotic index of cells classified as MAGE-A^+^ or SOX9^+^ in different regions of the tubule. Number of cells and percentage of population are tabulated from a pooled sample of 13 tissue sections.

Tubule region	MAGE-A			SOX9		
	Total	G1	Mitotic	Total	G1	Mitotic
Total area	11,780 (100)	5,826 (49.5)	5,954 (50.5)	10,124 (100)	8,977 (88.7)	1,147 (11.3)
Membrane-associated	8,976 (76.2)	4,639 (51.7)	4,337 (48.3)	7,934 (78.4)	6,993 (88.1)	941 (11.9)
Luminal	2,804 (23.8)	1,187 (42.3)	1,617 (57.7)	2,190 (21.6)	1984 (90.6)	206 (9.4)

SOX9 cells displayed a low mitotic index of 11.9 and 9.4% when calculated for membrane-associated and luminal cells, respectively (Table 1). This contrasts with MAGE-A cells, where the index was calculated at 48.3 and 57.7% for membrane-associated and luminal cells, respectively (Table 1). MAGE-A cells located towards the centre of the tubules had a 10% higher mitotic activity compared with membrane-associated cells (Table 1).

### Application of ANN-MLP- and CNN-based segmentation workflow to mouse testicular tissue

**Figure 6:**
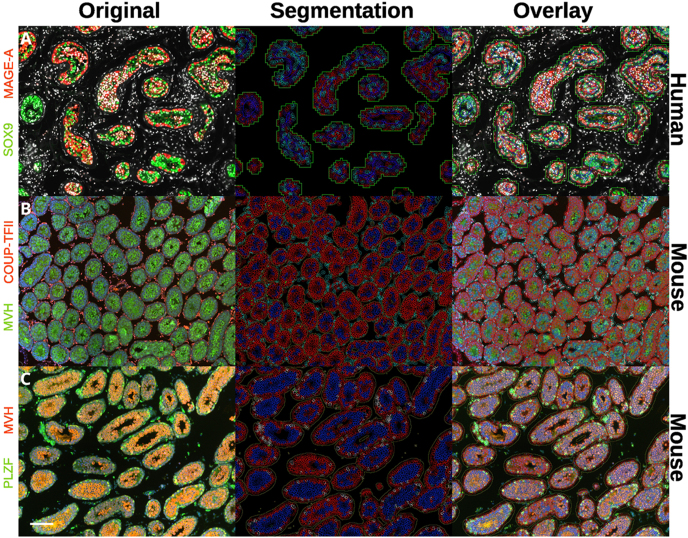
Application of automated tissue segmentation, cell segmentation and classification across different tissues and cellular markers. Left column – original immunofluorescent image and corresponding markers on the left. Middle column – tubule/cell segmentation and cell classification. Right column – overlay of segmentation and original image. (A) Human prepubertal testicular tissue stained with Hoechst, SOX9 (blue outline, green fluorescence, Sertoli cells), MAGE-A (light blue outline, red fluorescence, premeiotic germ cells) and unclassified (red outline). (B) Mouse testicular tissue stained with Hoechst, MVH (blue outline, green fluorescence, germ cells) and COUP-TFII (white outline, orange fluorescence, interstitial cells). (C) Mouse testicular tissue stained with Hoechst, MVH (blue outline, orange fluorescence, germ cells) and PLZF (white outline, green fluorescence, spermatogonia). The scale bar (bottom left) represents 50 μm. Mouse tissue was collected on postnatal day 17.

After establishing the practical application of QuPath’s automated methodologies in human prepubertal testicular tissue, we considered whether the same workflow could be applied to testicular tissues in other species. In particular, attention was given to mouse tissue due to the traditional translational workflows of progressing from animal experiments to those involving human tissues. In addition, it was important to establish if we could translate the methodology for use in other experimental conditions such as alternative fluorophore systems, microscopes or testicular cellular markers (Table S1 (see section on Supplementary materials given at the end of the article)). We found that running the same workflow in mouse tissue provided good tubule segmentation, accurate cell segmentation and classification (Fig. 6). However, a significant proportion of tubules are in close contact with each other and a small section would be bridged during segmentation (Figs S14 and S15). This could be further optimised based on the training annotations for the ANN-MLP. We found it useful to include the PTM cells in the training annotations as a defining border for segmenting tubules, which caused the bridging to occur. Bridged tubules in mouse tissues did not appear to impact significantly on downstream measurement as typically there were no additional cells between bridged tubules; therefore, cell counts and classifications were not artificially inflated. However, we have not validated this against other measurements such as those normalising to total tubular area. We trained the ANN-MLP on three small annotations per each ROI (tubules, tissue and background) on two images per group consisting of fully stained images (all fluorescent markers and counterstain) and antibody controls (Fig. 6A, B, C). However, a new model was trained for each marker/fluorophore combination (Table 2) as the pixel classifier would not run if different fluorescent channels were present compared to the training data. As before, segmented cells were then classified based on their fluorescent properties of intensity and location (Table 2) using QuPath’s object classification system, overlaid with the original image to gauge accuracy (Fig. 6).

**Table 2:** Fluorescence colour, location and corresponding classification colour for interstitial, germ, Sertoli and spermatogonial cells.

Image panel	Marker	Cell type	Fluorescence colour	Cell classification colour	Fluorescence location*	Tissue
A–C	SOX9	Sertoli cell	Green	Blue	Nuclear	Human
A–C	MAGEA	Premitotic germ cell	Red	White	Nuclear	Human
D–F	MVH	Germ cell	Green	Blue	Cytosol	Mouse
D–F	COUP-TFII	Interstitial cell	Red	White	Nuclear	Mouse
G–I	MVH	Germ cell	Orange	White	Cytosol	Mouse
G–I	PLZF	Spermatogonia	Green	Blue	Nuclear	Mouse

* UniProt, 2025.

### Comparing testicular image analysis methods: machine learning segmentation vs manual annotation in human testicular tissue

**Figure 7:**
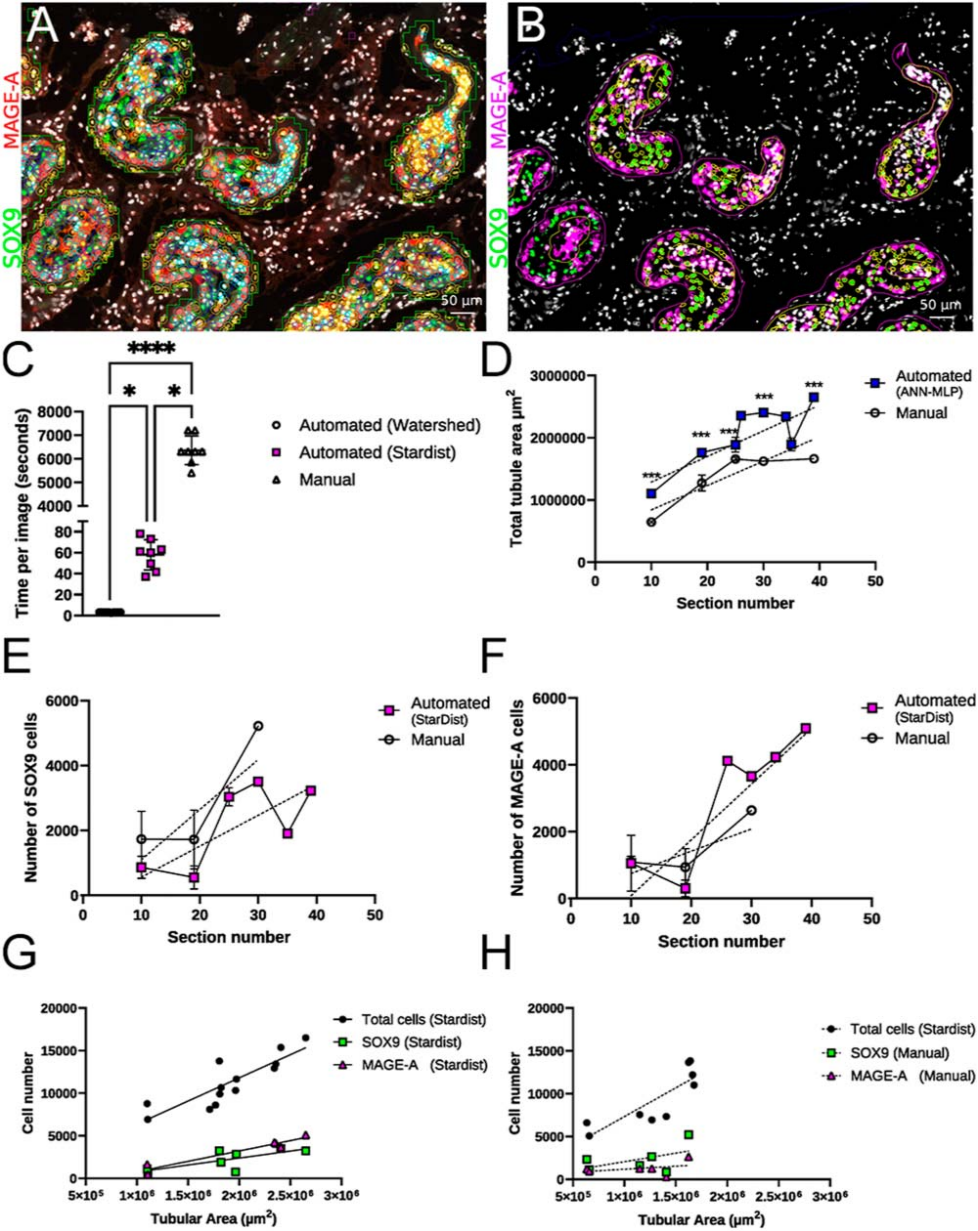
Comparison between the segmentation of tubules and classification of cells by automated or manual workflows: example of tubule segmentation performed by (A) ANN-MLP pixel classification. (B) Manual annotation. (C) The time taken in seconds to perform segmentation of tubules and cells and fluorescence-based classification of cells analysed by the Kruskal–Wallis test. (D) Total tubular area segmented either by ANN-MLP pixel classification (*r*^2^ = 0.70) or by manual annotation (*r*^2^ = 0.76) analysed by two-way ANOVA. (E) Number of SOX9^+^ cells identified per section number in tubules defined by ANN-MLP pixel classification (*r*^2^ = 0.51) or by manual annotation (*r*^2^ = 0.52) analysed by two-way ANOVA. (F) Number of MAGE-A^+^ cells identified per section number in tubules defined by ANN-MLP pixel classification (*r*^2^ = 0.70) or by manual annotation (*r*^2^ = 0.41) analysed by two-way ANOVA. (G) Scatter plot to correlate total cell number (*r*^2^ = 0.74, *P* = 0.0002), SOX9^+^ (*r*^2^ = 0.56, *P* = 0.03) and MAGE-A^+^ (*r*^2^ = 0.93, *P* = 0.002) cells per tubular area defined by ANN-MLP segmentation analysed by linear regression. (H) Scatter plot to correlate total number of cells (*r*^2^ = 0.71, *P* = 0.0046) defined by StarDist with SOX9^+^ (*r*^2^ = 0.26, *P* = 0.31) and MAGE-A^+^ (*r*^2^ = 0.14, *P* = 0.46) cells per tubular area quantified by manual annotation and analysed by linear regression. *P =< 0.05, ***P = 0.001, ****P =< 0.001. In panels (C, D, E, F), error bars represent standard deviation. In panels (D, F, G, H), the dotted lines represent linear regression.

Finally, we wanted to compare our ANN-MLP/StarDist-based workflow to existing established methods of manual annotation (Kearney *et al.* 2025) and classification of cells from testicular tissue sections. To determine if there were any improvements in the time required or accuracy of analysis, a series of images from freshly fixed prepubertal human testicular tissues were analysed (Fig. 7A and B). The time taken to analyse the same set of images was measured, and this demonstrated a significant difference between groups, which was dependent on the segmentation method used for tubules or cells (Fig. 7C). The biggest difference was seen when comparing the ANN-MLP pixel classification system for segmenting tubules, used by both StarDist and Watershed, to manual annotation. On average, both ANN-MLP methods were significantly faster than manually annotating tubules for StarDist (6,247 s per image faster, *P* = 0.022, Kruskal–Wallis test) and for Watershed (6,297 s per image faster, *P*=<0.0001, Kruskal–Wallis test). The difference in computation intensity of Watershed vs StarDist for segmenting cells also further separated the two groups with Watershed being on average 50 s per image faster to segment cells than StarDist (Fig. 7C, *P* = 0.0217, Kruskal–Wallis test). Furthermore, the segmented tubular area from either ANN-MLP pixel classification and manual annotation was compared (Fig. 7D). We found that the manually annotating images consistently and significantly had a lower tubular area compared to ANN-MLP-based segmentation (*P* = 0.001, two-way ANOVA). This was attributed to the ANN-MLP segmentation generally including the peritubular myoid cells, whereas manual annotations did not as boundaries were drawn much tighter to fluorescence staining of the SOX9^+^ and MAGE-A^+^ cells. In addition, we noted that as the sections went deeper into the tissue the tubular area plateaued, whereas the ANN-MLP segmentation continued a trend of increasing tubular area. Similarly, we compared the number of SOX9^+^ (Fig. 7E) and MAGE-A^+^ (Fig. 7F) cells at each tissue depth based on section number. Overall, we found no statistical difference in the detection of either cell type. As part of the automation process, all cells within the tubular area are segmented, which allowed us to determine that total cell number significantly positively correlates with tubular area for StarDist segmentation (Fig. 7G, *P* = 0.0002, *r*^2^ = 0.74). Similarly, SOX9^+^ (*r*^2^ = 0.56, *P* = 0.038) and MAGE-A^+^ (*r*^2^ = 0.92, *P* = 0.002) cells segmented by StarDist showed a similar statistically significant positive correlation with tubular area. In contrast, SOX9^+^ (*r*^2^ = 0.26, *P* = 0.31) and MAGE-A^+^ (*r*^2^ = 0.14, *P* = 0.46) cells identified by manually annotating tubules showed no statistically significant correlation with tubular area (Fig. 7H). To determine if the annotation method or cell detection method was causing poor correlation in tubular area and cell counts, we applied StarDist to segment all cells in the manually annotated tubules. StarDist total cell numbers from manual annotations were plotted alongside the SOX9^+^ and MAGE-A^+^ manual counts (Fig. 7H). Total cell number from StarDist in the manual annotations showed a statistically significant correlation between cell number and tubular area (*r*^2^ = 0.71, *P* = 0.0046). This was similar to the automated tubule segmentation and cell segmentation workflow highlighting a potential weakness in the manual cell classification method for SOX9^+^ and MAGE-A^+^ cells. For example, non-linear data can be removed by plotting tubular area vs number of detections in control samples (Fig. 7G and H).

## Discussion

In recent years, machine learning has increasingly received a large amount of attention due to its successful application in fields such as histopathology analysis, multi-objective optimisation and prediction models for investment return (Chen *et al.* 2025, Duan *et al.* 2025, Komura *et al*. 2025, Dang *et al.* 2026, Huang *et al.* 2026, Sun *et al.* 2026). In the context of immunofluorescent image analysis, machine learning represents an exceptionally powerful tool, but is often out of the reach for a large number of researchers due to the technical skills required by the end user for deep learning or the large datasets and annotations needed for training (Komura *et al*. 2025). As an example, recent work on tubular staging (classifying tubules on their stage of spermatogenesis) has been performed with a deep learning model using IF or H&E images as input. One group suggests that training required 3,097 images (Meikar *et al.* 2023), another required 27,873 (Meikar *et al.* 2023), and a third group required the manual annotation of 7,800 cells and over 2,000 tubules (Yang *et al.* 2022). Despite the fact that not all images will be unique, often using transformations such as rotation or scaling of training images to ensure correct performance, it still represents a large volume of data and additional complexity that is needed for deep learning. In contrast, the methodology presented in this paper has managed to achieve reasonable tubule segmentation with training on only 6 images and as few as 3 annotations per image and area of interest.

The trained ANN-MLP model presented here does have some limitations when segmenting densely packed tubules such as those found in mouse testis tissue. Where tubules are in close contact, the ANN-MLP model resulted in ‘bridging’ between tubules (Figs S1B, S2, S3, S4, S5, S6, S7, S8, S9, S10, S11, S12, S13, S14, S15B). This could be potentially corrected by optimising the training model’s annotation (not including PTM cells, for example) to give a tighter segmentation of tubules. An additional limitation of this model is the lack of training to handle artefacts from tissue processing, such as damage, or to correct issues with images being out of focus or over/under exposed. We believe ANN-MLPs can be modified to further discriminate tissue areas with training, but this can also be achieved by manually reviewing the segmentation if necessary. Under-staining regions, or inconsistent staining across tissue, do present a challenge as currently it is only possible to identify these during the data analysis phase for tissue sections that do not follow the tubular area/cell density correlation (Fig. 7 E, F, G, H). This contrasts with the manual annotation method, where the user is able to discriminate if tubules or tissue sections should progress through the segmentation and cell classification steps.

Another factor to consider when using or developing machine learning models is cost from both capital investment and time. One group reference their use of a workstation with 128 GB of RAM and an Nvidia A100 GPU (Lu *et al.* 2022). Given the current AI demands on computer resources, this represents at £10,000–15,000 investment in the GPU alone. To try and alleviate this cost, it has been shown that some of these models can run in free cloud compute instances (Meikar *et al.* 2023). Researchers also highlight the cost for annotating training datasets. The expense and time required by a histology expert for annotations was noted to be a limiting factor. In a move towards accessibility, some groups have bundled their work into a standalone package for MATLAB (Yang *et al.* 2022); this presents the deep learning model as a highly specialised app-like experience. In contrast, our study supports the use of software that provides general imaging analysis tools to researchers, such as QuPath (Bankhead *et al.* 2017). In particular, we found the synergistic combination of ANN-MLPs for image segmentation and publicly available convolutional neural networks (CNNs) for nuclei and cell segmentation (Schmidt *et al.* 2018, Weigert *et al.* 2020, Weigert & Schmidt 2022) combined with QuPath’s wide range of image analysis tools and ability to run on consumer grade computers (e.g. laptops), provided a significantly more accessible and flexible workflow to leverage machine learning for potentially a wide range of testicular research. For example, in a mature tubule, we would expect to find a significant proportion of haploid cells towards the lumen as spermatogenesis initiates. In this instance, given the tissue is prepubertal, it is not unexpected to see a greater range of mitotic activity (Fig. 5) throughout the tubule (Griswold 2016). In addition, cell population with a higher RNA content such as those undergoing differentiation and active translation could be stained with Pyronin Y and Hoechst 33342 to give a greater level of detail into differentiation and mitotic activity (Kim & Sederstrom 2015).

A final area to consider is the time saved from using computation tools to analyse images in comparison with humans performing the same analysis. Researchers utilising a deep learning model for testis staging have shown to cut down image processing time from 3 h/image for humans to 1.9 h/image using a deep learning model (Xu *et al.* 2021). In our project, the manual data analysis of each image used took on average 105 min to complete, depending on complexity. The machine learning methodology presented here offers a method that can segment tubules in both mouse and human samples instantaneously (<1 s). When considering the total time needed for cell segmentation using StarDist and training of the pixel classifier, the same quantification would take less than one hour with the proposed machine learning workflow compared to 25 h using the manual method. Based on our experience, an automated ANN-MLP/StarDist workflow could complete the same minimum analysis in as little as 2 h when factoring in classifier training and could be even less if pre-trained classifier models were available to use without the need to retrain. Therefore, this methodology significantly improves the speed and accessibility for researchers performing computational-based image analysis.

## Conclusion

We describe a machine learning approach to testicular image analysis that provides a fast and accurate method for quantification of key parameters. This can reduce the burden on the end user from a time investment perspective, compared with traditional image analysis methods. This computational-based image analysis approach using open-access software can be used by researchers for cell quantification across a range of testicular tissues.

## Supplementary materials





## Declaration of interest

The authors declare that there is no conflict of interest that could be perceived as prejudicing the impartiality of the work reported.

## Funding

RTM was supported by a UK Research and Innovation (UKRI) Future Leaders Fellowship (Grant Reference: MR/Y011783/1). For the purpose of open access, the author has applied a Creative Commons Attribution (CC BY) licence to any Author Accepted Manuscript version arising from this submission.

## Author contribution statement

AJRG was involved in conceptualisation, investigation, validation, data curation, formal analysis, visualisation, writing of the original draft and supervision of EB. IS was involved in investigation, formal analysis and review and editing. EB was involved in investigation and review and editing. JD provided resources. AL performed investigation and reviewed and edited the manuscript. KD and AS supervised AL and were involved in review and editing. RTM was involved in funding acquisition, data interpretation, project administration, resources and review and editing.
