# Supplementary material for: Machine learning and automation methods for the segmentation, classification and quantification of testicular tissue sections
Source: Reprod Fertil. 2026 May 29;7(2):RAF250210. doi: 10.1530/RAF-25-0210 (PMC13232597; doi:10.1530/RAF-25-0210)

Machine learning and automation methods for the segmentation, classification and quantification of testicular tissue sections

Running title: Machine learning for quantification of testicular tissues

Authors: Adam J. R. Gadd[1], Iris Sanou[2,3], Eleanor Brain[4], Jill Davies[5], Adomas Liugaila[6], Kathleen Duffin[1,4], Agnes Stefansdottir [6], Rod T. Mitchell[1,4*]

[1] Centre for Reproductive Health, Institute for Regeneration and Repair, The University of Edinburgh, Scotland, UK

[2] Reproductive Biology Laboratory, Centre for Reproductive Medicine, Amsterdam UMC, University of Amsterdam, the Netherlands

[3] Amsterdam Reproduction & Development Research Institute, Amsterdam UMC, University of Amsterdam, The Netherlands

[4] Royal Hospital for Children & Young People, Edinburgh, Scotland, UK

[5] Oxford Cell and Tissue Biobank, John Radcliffe Hospital, Oxford University Hospitals NHS Trust, Oxford, UK

[6] Biomedical Sciences, Edinburgh Medical School, The University of Edinburgh, Scotland, UK

Supplementary information:

Ethics and tissue preparation

Human – Tissue samples were collected under ethical approval from South-East Scotland Research Ethics Committee (13/SS0145) and Oxford University Hospitals NHS Foundation Trust (2016/0140). Written consent was obtained from parents/guardian or patients (where appropriate).

Prepubertal testicular tissue was collected from patients undergoing a testicular biopsy for fertility preservation at Royal Hospital for Sick Children in Edinburgh and John Radcliffe Hospital in Oxford. Approximately 10% of the biopsy was allocated for research purposes and transported in either Nutristem® hSEC XF Medium (Biological industries) supplemented with 1% penicillin/streptomycin (Edinburgh samples) or in Hank's Balanced Salt solution (HBSS) with 10% human serum albumin (Oxford samples). Tissues were then fixed in Bouins for three hours, embedded in paraffin wax and cut into 5 µm sections using a microtome.

The data presented here is from a 13 year old patient.

Mouse - All experiments were approved by the University of Edinburgh's Local Ethical Review Committee and carried out in accordance with UK Home Office regulations under the ASPA 1986 act. Wild-type CD-1 mice were maintained and bred in an environmentally-controlled room on a 14-hour light:10-hour dark photoperiod. Mice were culled on postnatal day 17. Testes were collected in 1x phosphate-buffered saline (PBS; Fisher Scientific UK Ltd). The testes were fixed in 10% neutral-buffered formalin solution (Sigma Aldrich Ltd) for 24 hours. After fixation, all tissue was processed, embedded into wax, cut using microtome (Leica RM2255) at 5 µm thickness and placed on microscope slides.

Immunofluorescence

Immunofluorescence was carried out to identify a range of cell types including germ, interstitial, Sertoli, spermatogonial and SSC populations in the testis of both human and mouse tissue (Table 1).

Human Tissue

Sections were dewaxed by incubating slides in xylene (2 x 5 min), then rehydrated by incubating slides in a decreasing alcohol series:100%, 95%, 80%, 70% alcohol for 20 sec each. Slides were transferred to TBS prior to heat induced antigen retrieval achieved by incubating slides in a 0.01 M citrate buffer (pH 6) in a pressure cooker for 20 min. Slides were transferred back to TBS and

washed (2 x 5 min). Blocking of endogenous peroxidase was achieved with a methanolic peroxide solution (10 v/v% peroxide in Methanol) for 15 min. Slides were washed again in TBS (2 x 5 min), tissue sections were outlined with hydrophobic barrier pens (Kisker biotech GMBH) to reduce antibody volumes required before blocking with SuperblockTM (Thermo Scientific) in TBS for 30 min. Primary antibodies were diluted in SuperblockTM and incubated on tissue sections overnight at 4 °C (Table S1). Slides were then washed in TBS (2 x 5 min) and peroxidase conjugated secondary antibodies (diluted in Superblock) were added to slides for 30 min. The Opal amplification system (Akoya Biosciences) was used at 1:100 dilution in 1X Plus Amplification diluent (Akoya Biosciences) and incubated for 10 min before washing in TBS. Dual colour staining was achieved by repeating antigen retrieval, peroxidase block, primary and secondary antibody incubation steps. Fully stained slides were counterstained with Hoechst 33342 (16 µM, 10 µg/mL in TBS) for 30 min and washed with TBS (2 x 5 min). Coverslips were mounted with Prolong Diamond antifade mounting media (Invitrogen) and cured for 48 hours prior to imaging at 20x magnification on a Zeiss observer epifluorescent microscope with Zen software (Zeiss). Two negative control slides were included for each of the immunohistochemistry experiments: one without primary antibodies and one without secondary antibodies.

Mouse tissue

Sections were dewaxed by incubating slides in xylene (2 x 5 min), then rehydrated by incubating slides in a decreasing alcohol series:100%, 95%, 80%, 70% alcohol for 20 sec each. Slides were transferred to PBS. Slides were blocked with blocking solution for one hour (See Table S1 for details). Then primary antibodies were diluted in blocking solution and applied to the tissue sections overnight at 4 °C in a humidified environment (Table S1). The next day, slides were washed in distilled H_2O (dH_2O) and 1x PBST for 5 min each. Secondary antibodies were diluted in the blocking solution and applied to the sections for one hour at room temperature. Slides were washed in 1x PBST (2 x 5 min) and submerged in 4',6-diamidino-2-phenylindole (DAPI; Invitrogen) diluted

1:5,000 in 1x PBS for 5 min to counterstain the nuclei then washed in 1x PBS (2 x 5 min). Slides were dabbed dry, Vectashield (Vector Laboratories) was applied and the slides coverslips were applied. Every tenth section was imaged using an inverted microscope at 20x magnification (ECLIPSE Ti2; Nikon Instruments). Two negative control slides were included for each of the immunohistochemistry experiments: one without primary antibodies and one without secondary antibodies.

Immunofluorescence reagents

Table S1: Antibodies and blocking reagents used during immunofluorescence staining of human and mouse testicular samples

Tissue	Cell Type	Target	Primary antibody	Dilution	Secondary	Dilution	Opal	Blocking
Human	Premeiotic germ cells	Melanoma-associated antigen (MAGE-A)	MAGE-A3 (Merck, mabc1150)	1:1,000	Goat anti-mouse HRP (Dako, P0447)	1:200 (Superblock)	Opal 650 (Akoya bioscience, OP-001005)	Superblock TBS (thermo scientific, 37581)
	Sertoli cells	SRY-Box Transcription Factor 9 (SOX9)	SOX9 (Merck, ab5535)	1:10,000	Goat anti-rabbit HRP (PI-1000-1)	1:200	Opal 520 (Akoya bioscience, OP-001001)	Superblock TBS
Mouse	Germ cell	Mouse vasa homologue (MVH)	MVH (ab13840; Abcam)	1:200	AF568 goat anti-rabbit (A11011; Invitrogen)	1:200	NA	20% goat/ 5% bovine serum, PBS-T (Merk)
			MVH (ab27591; Abcam)	1:200	AF488 goat anti-mouse (A21124; Invitrogen)	1:200	NA	20% goat/ 5% bovine serum, PBS-T (Merk)
	Interstitial cells	COUP transcription factor II (COUP-TFII)	COUPTFII (PP-H7147-00; R&D Systems)	1:200	AF568 goat anti-rabbit (A11011; Invitrogen)	1:200	NA	20% goat/ 5% bovine serum, PBS-T (Merk)
	Spermatogonia	Promyelocytic Leukemia Zinc Finger protein (PLZF)	PLZF (ab189849; Abcam)	1:4,000	AF568 goat anti-rabbit (A11011; Invitrogen)	1:200	NA	20% goat/ 5% bovine serum, PBS-T (Merk)

Original microscopy images

Figure S1A – Human prepubertal testicular tissue stained for nuclei (white), SOX9 (green) and MAGE-A (red). 2023-0248 PCC 10 1.

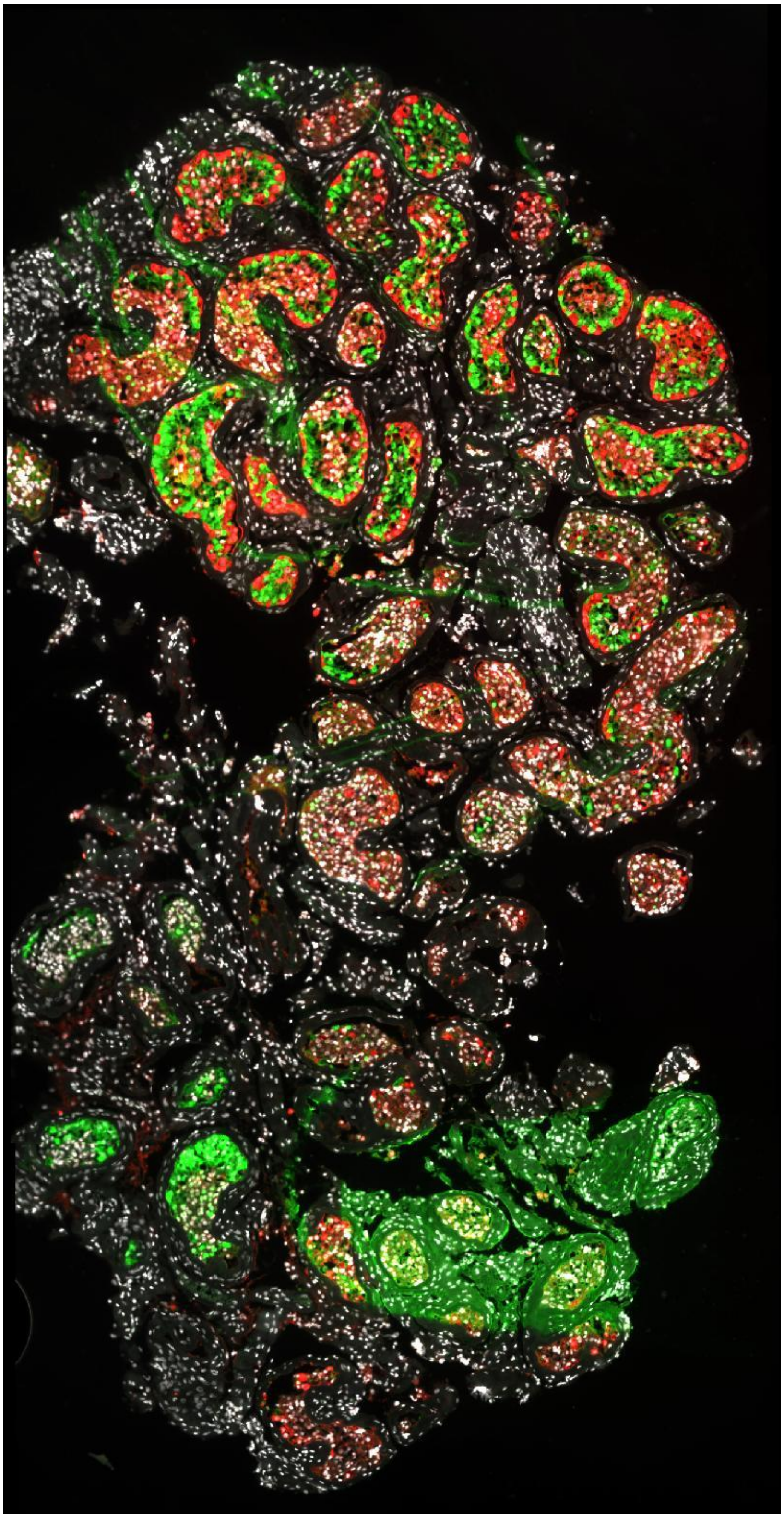

Figure S1B – Human prepubertal testicular tissue stained for nuclei (white), SOX9 (green) and MAGE-A (red). 2023-0248 PCC 10 1.

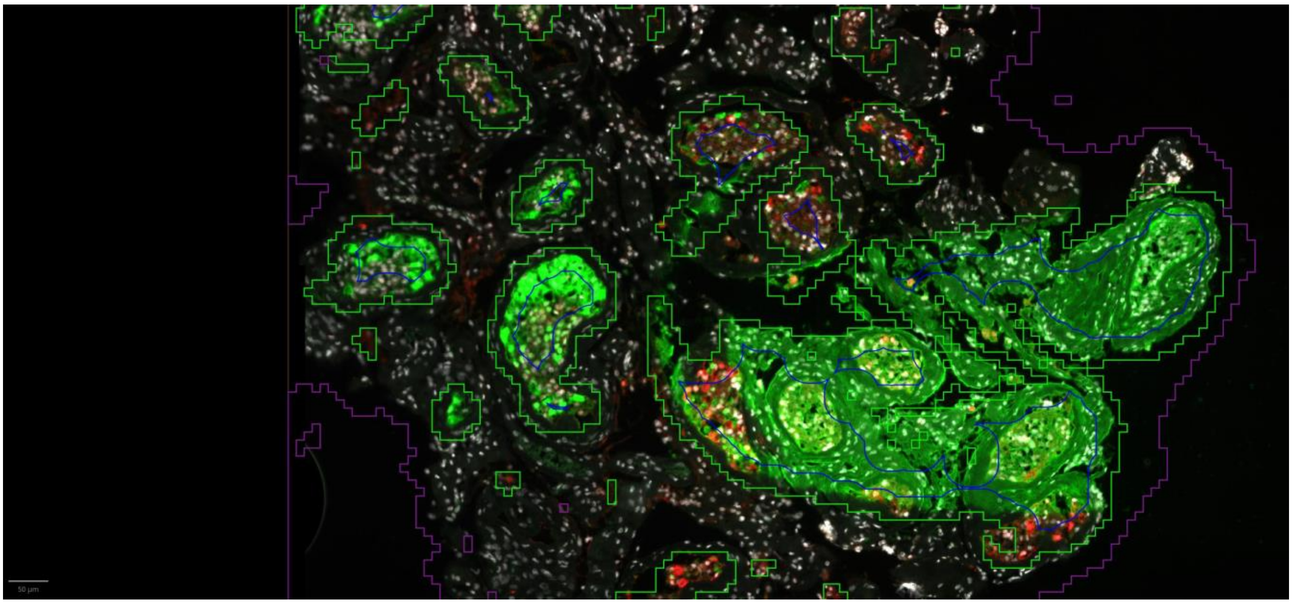

Figure S2A - Human prepubertal testicular tissue stained for nuclei (white), SOX9 (green) and MAGE-A (red). 2023-0248 PCC 10 2

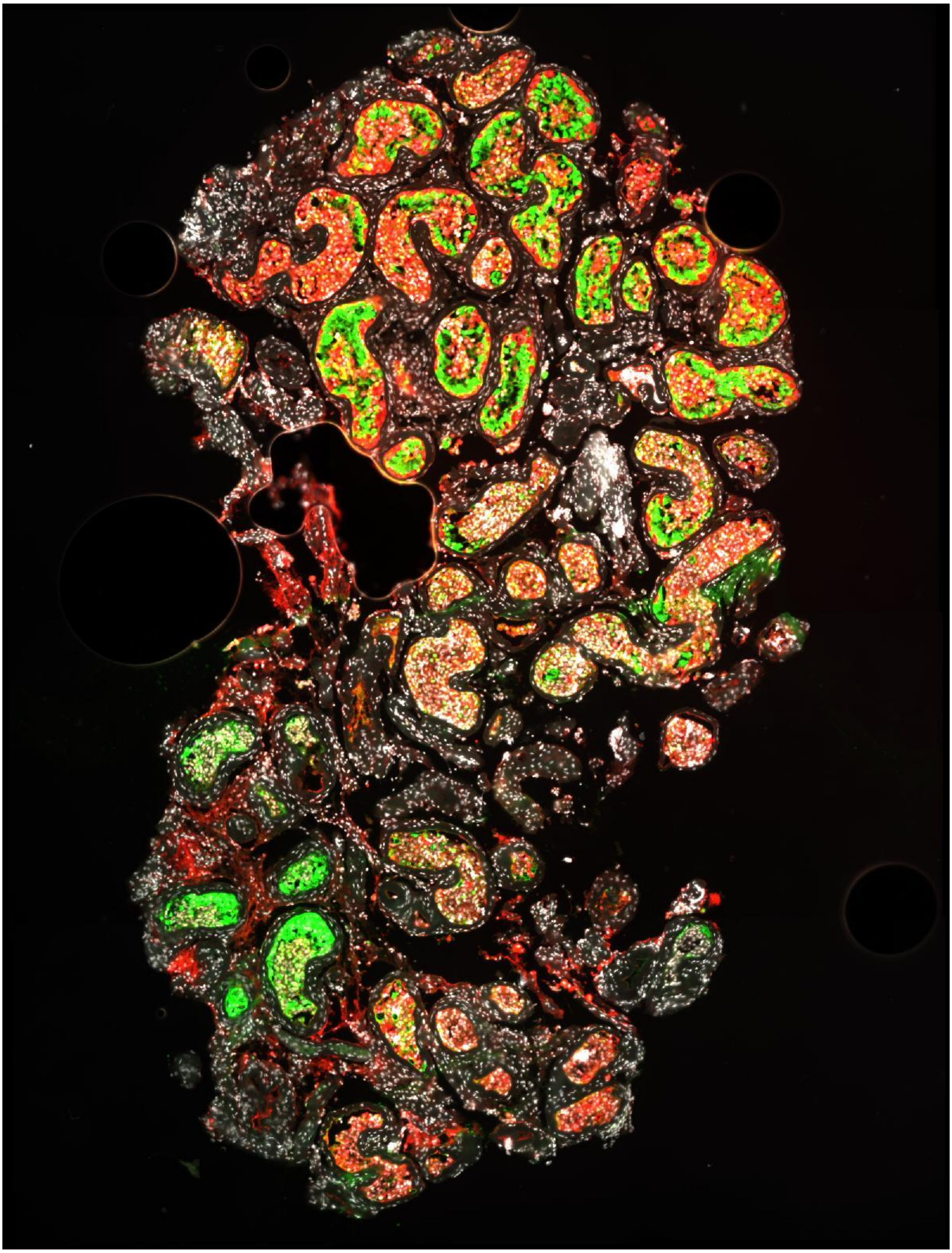

Figure S2B - Human prepubertal testicular tissue stained for nuclei (white), SOX9 (green) and MAGE-A (red). 2023-0248 PCC 10 2

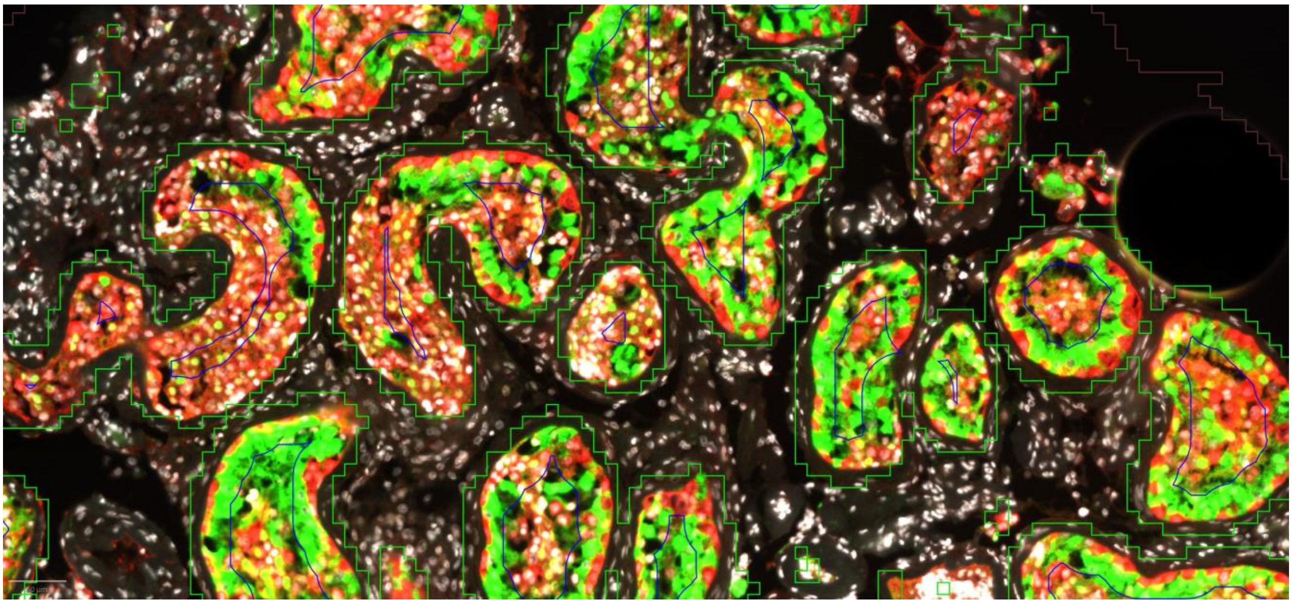

Figure S3A - Human prepubertal testicular tissue stained for nuclei (white), SOX9 (green) and MAGE-A (red). 2023-0248 PCC 19 1

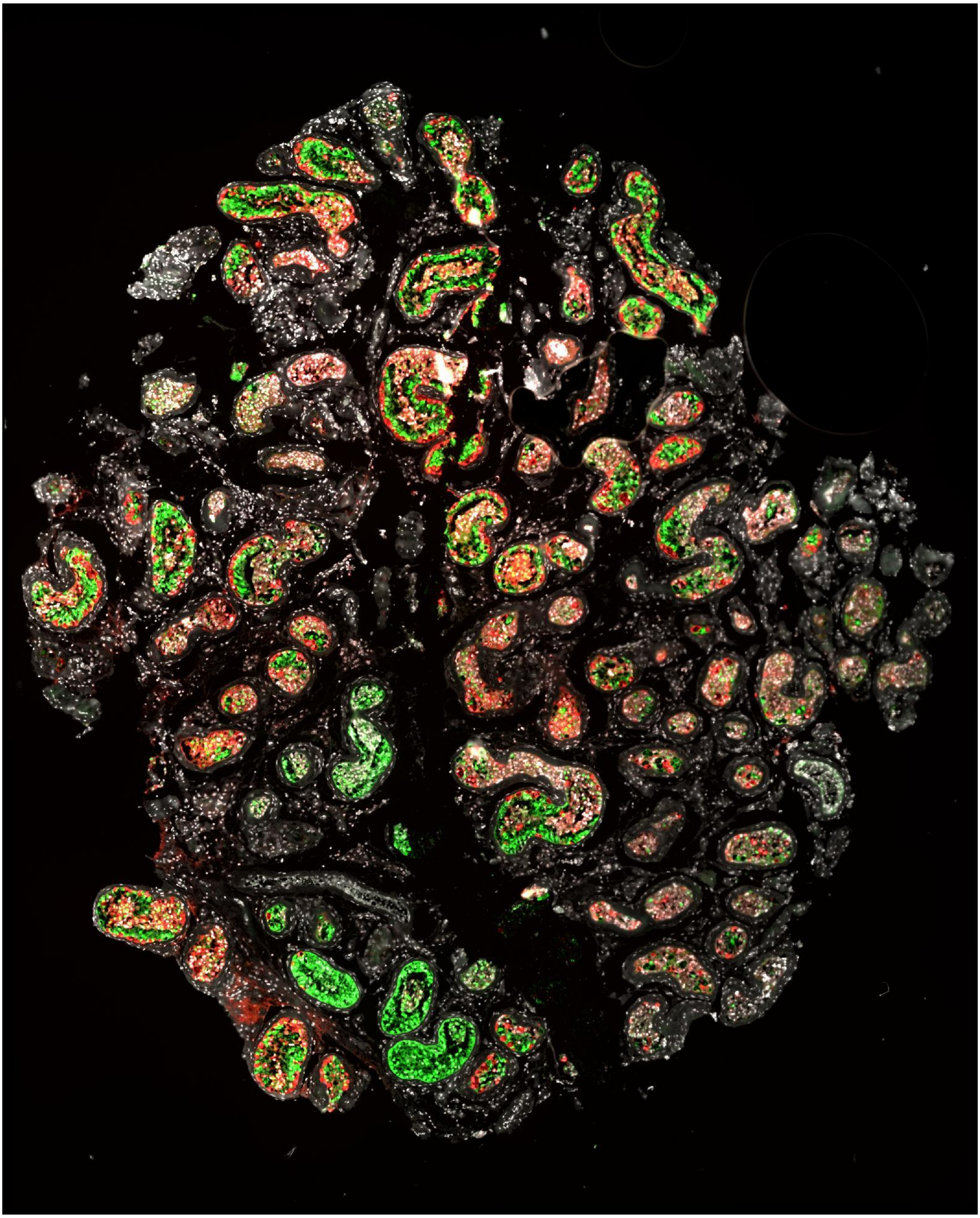

Figure S3B - Human prepubertal testicular tissue stained for nuclei (white), SOX9 (green) and MAGE-A (red). 2023-0248 PCC 19 1

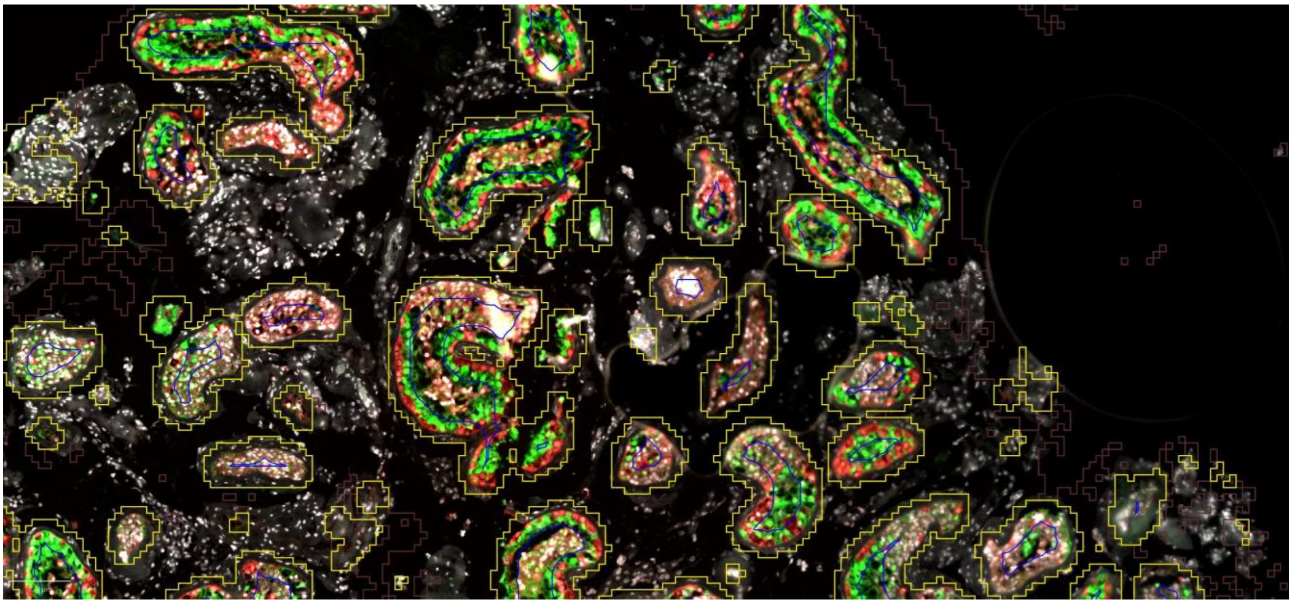

Figure S4A - Human prepubertal testicular tissue stained for nuclei (white), SOX9 (green) and MAGE-A (red). 2023-0248 PCC 19 2

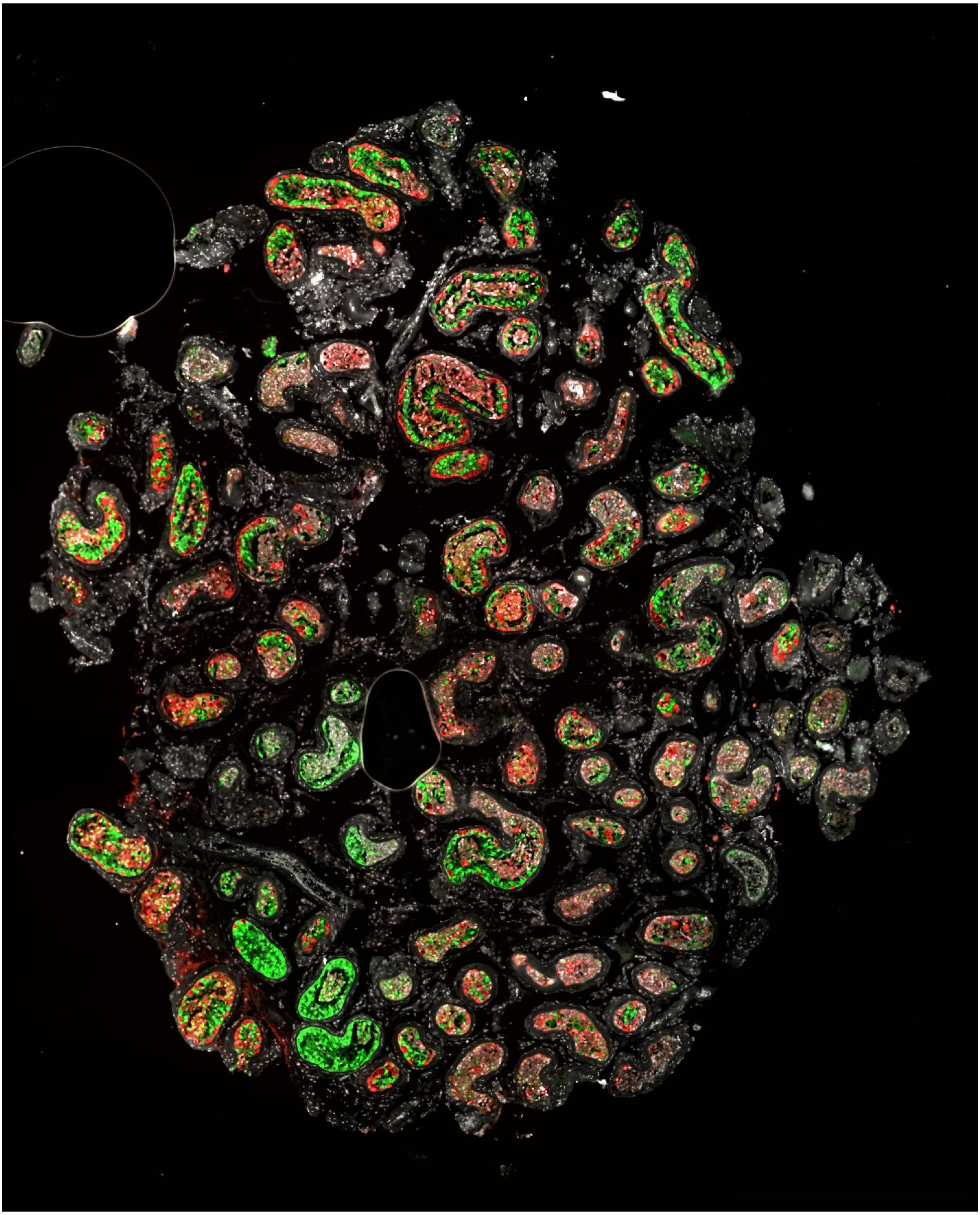

Figure S4B - Human prepubertal testicular tissue stained for nuclei (white), SOX9 (green) and MAGE-A (red). 2023-0248 PCC 19 2

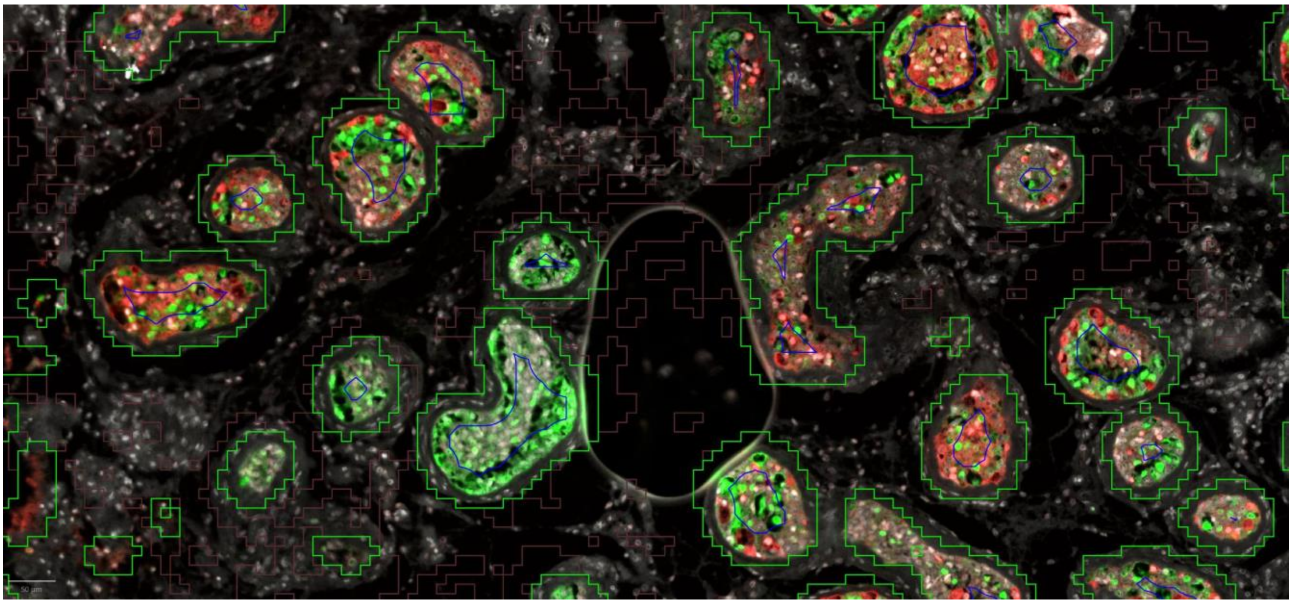

Figure S5B - Human prepubertal testicular tissue stained for nuclei (white), SOX9 (green) and MAGE-A (red). 2023-0248 PCC 19 3

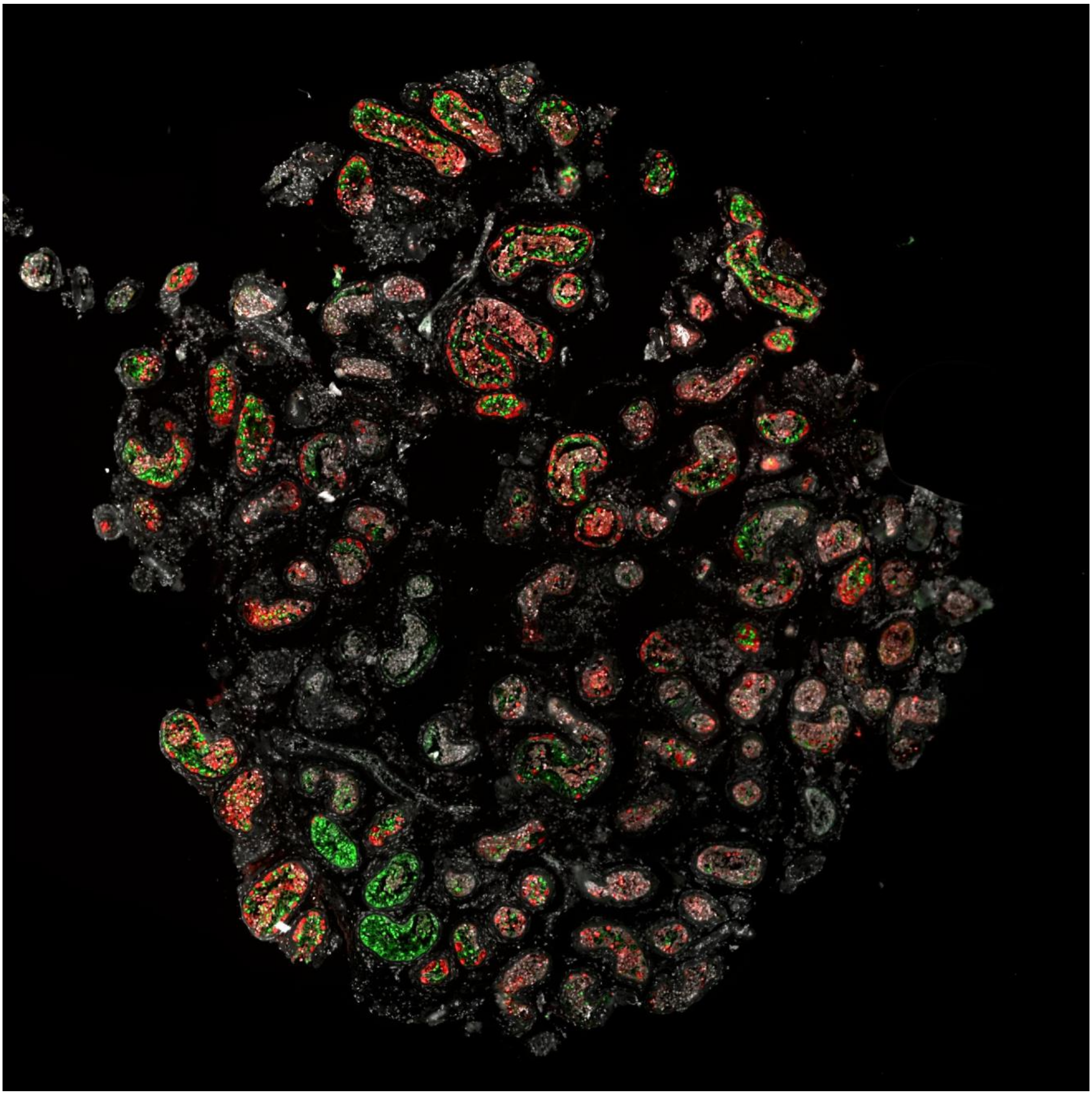

Figure S5B - Human prepubertal testicular tissue stained for nuclei (white), SOX9 (green) and MAGE-A (red). 2023-0248 PCC 19 3

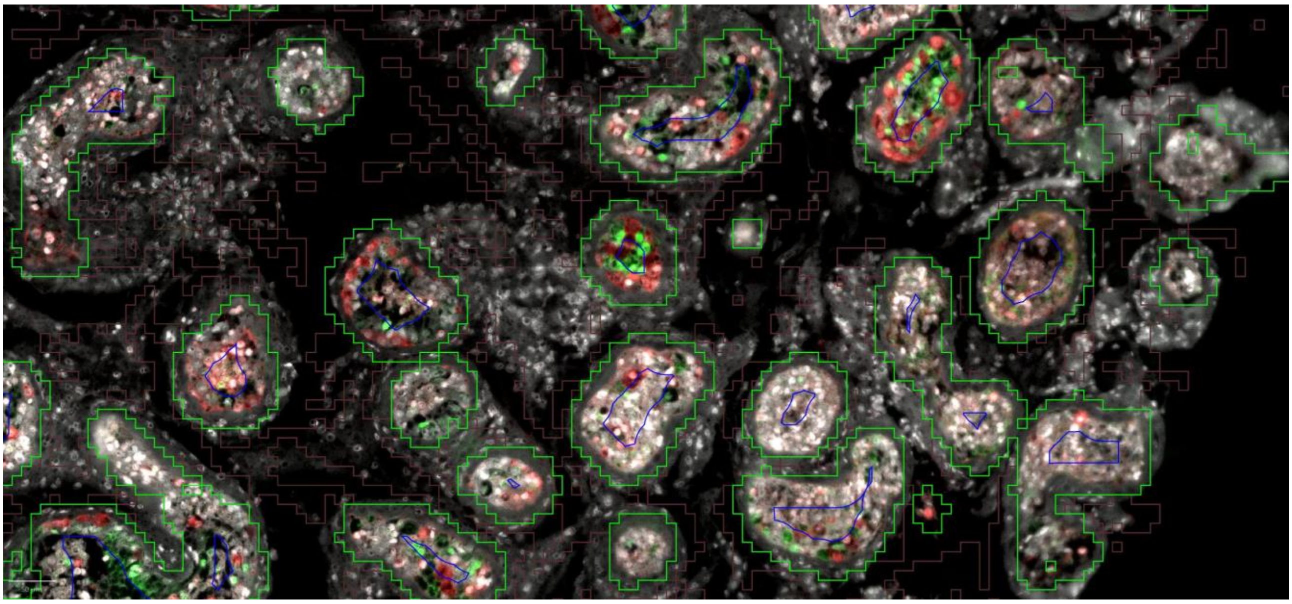

Figure S6A - Human prepubertal testicular tissue stained for nuclei (white), SOX9 (green) and MAGE-A (red). 2023-0248 PCC 25 PC2 1 – Primary antibody control, no MAGE-A primary antibody

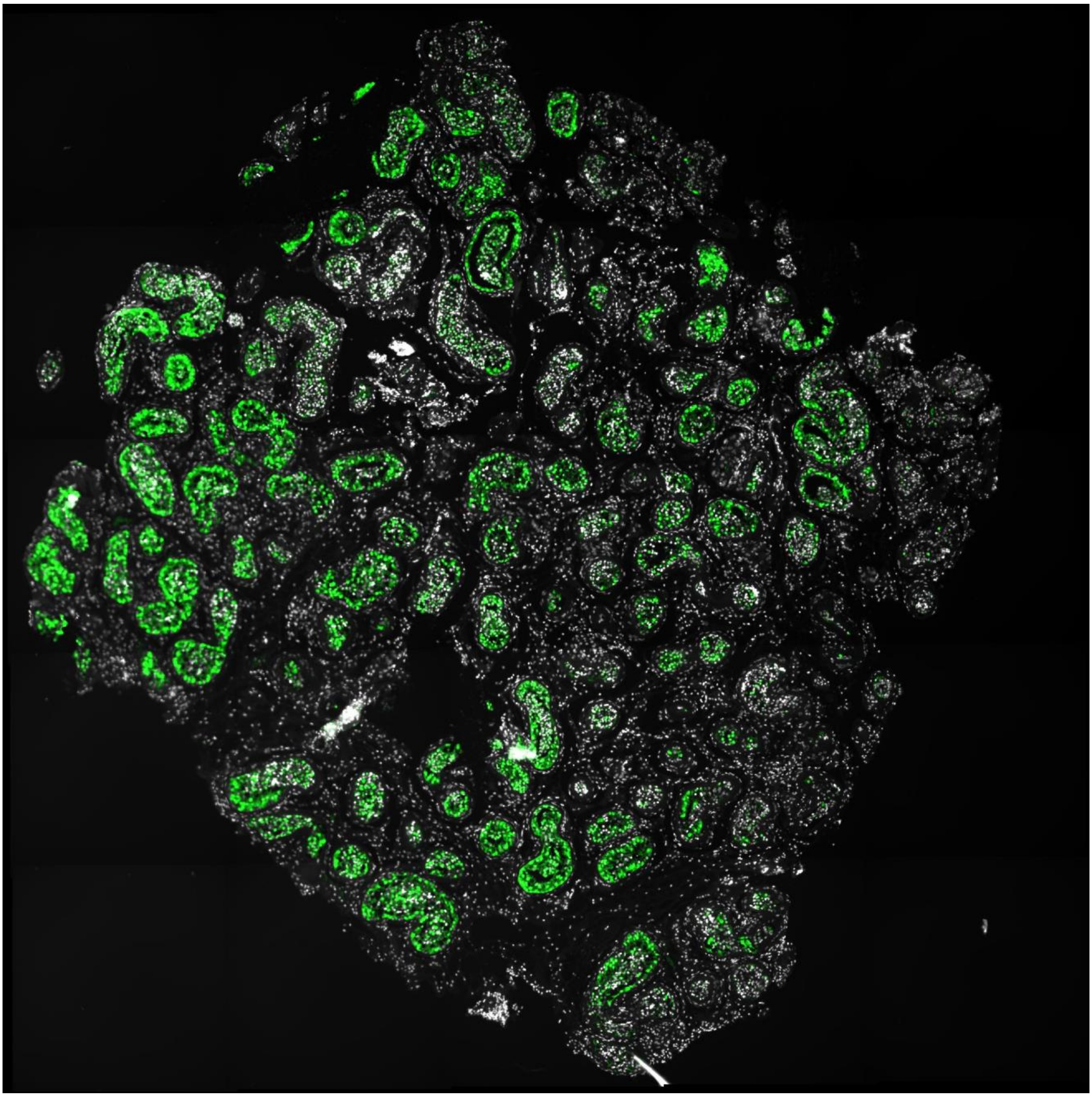

Figure S6B - Human prepubertal testicular tissue stained for nuclei (white), SOX9 (green) and MAGE-A (red). 2023-0248 PCC 25 PC2 1 – Primary antibody control, no MAGE-A primary antibody

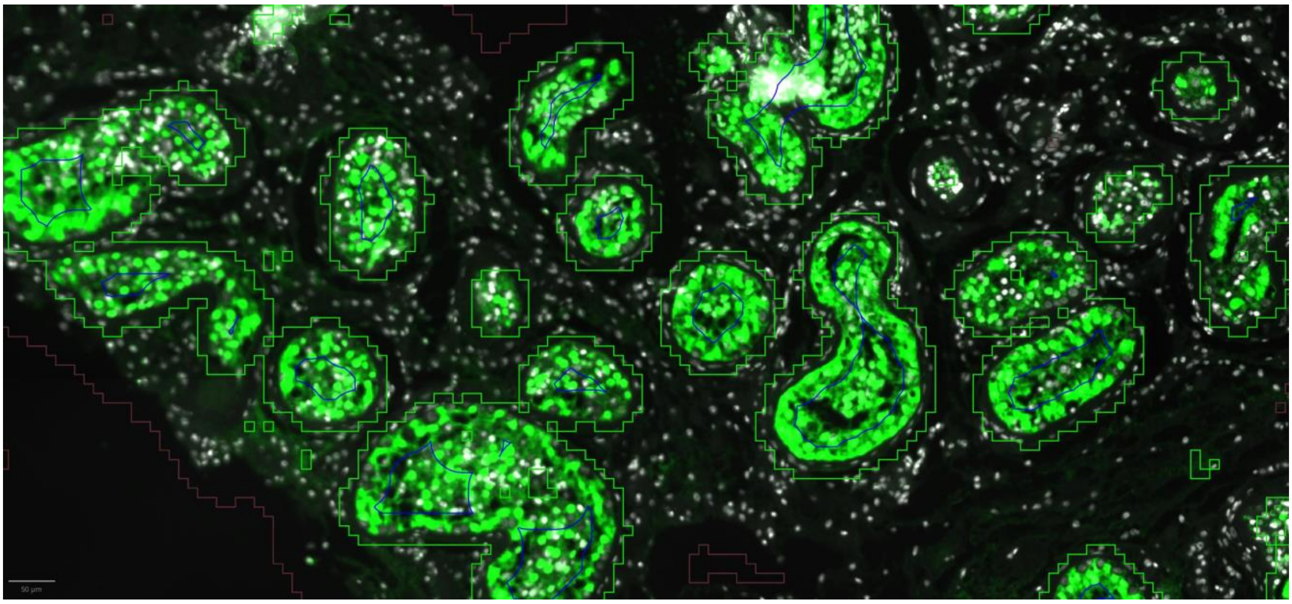

Figure S7B - Human prepubertal testicular tissue stained for nuclei (white), SOX9 (green) and MAGE-A (red)2023-0248 PCC 25 PC2 2. Primary antibody control, no MAGE-A primary antibody

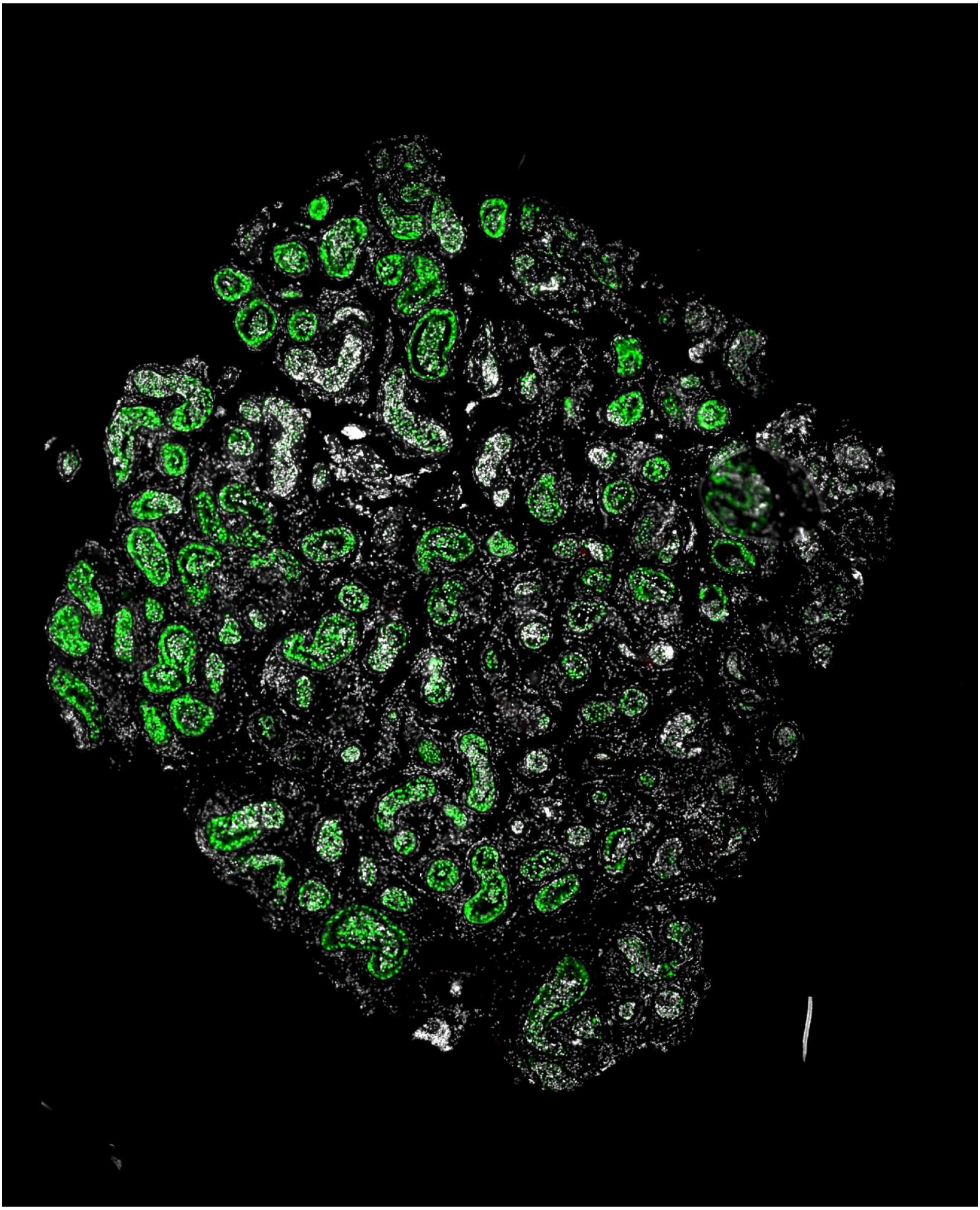

Figure S7B - Human prepubertal testicular tissue stained for nuclei (white), SOX9 (green) and MAGE-A (red)2023-0248 PCC 25 PC2 2. Primary antibody control, no MAGE-A primary antibody

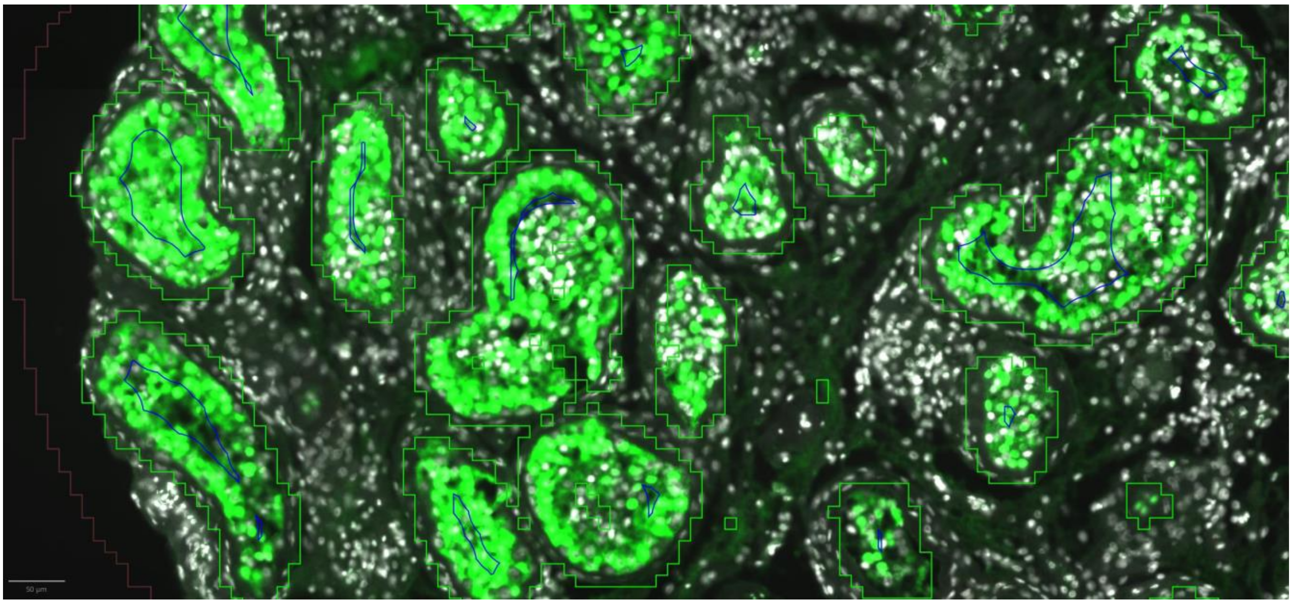

Figure S8A - Human prepubertal testicular tissue stained for nuclei (white), SOX9 (green) and MAGE-A (red) 2023-0248 PCC 26 SC1 1. Secondary antibody control, no SOX9 secondary antibody

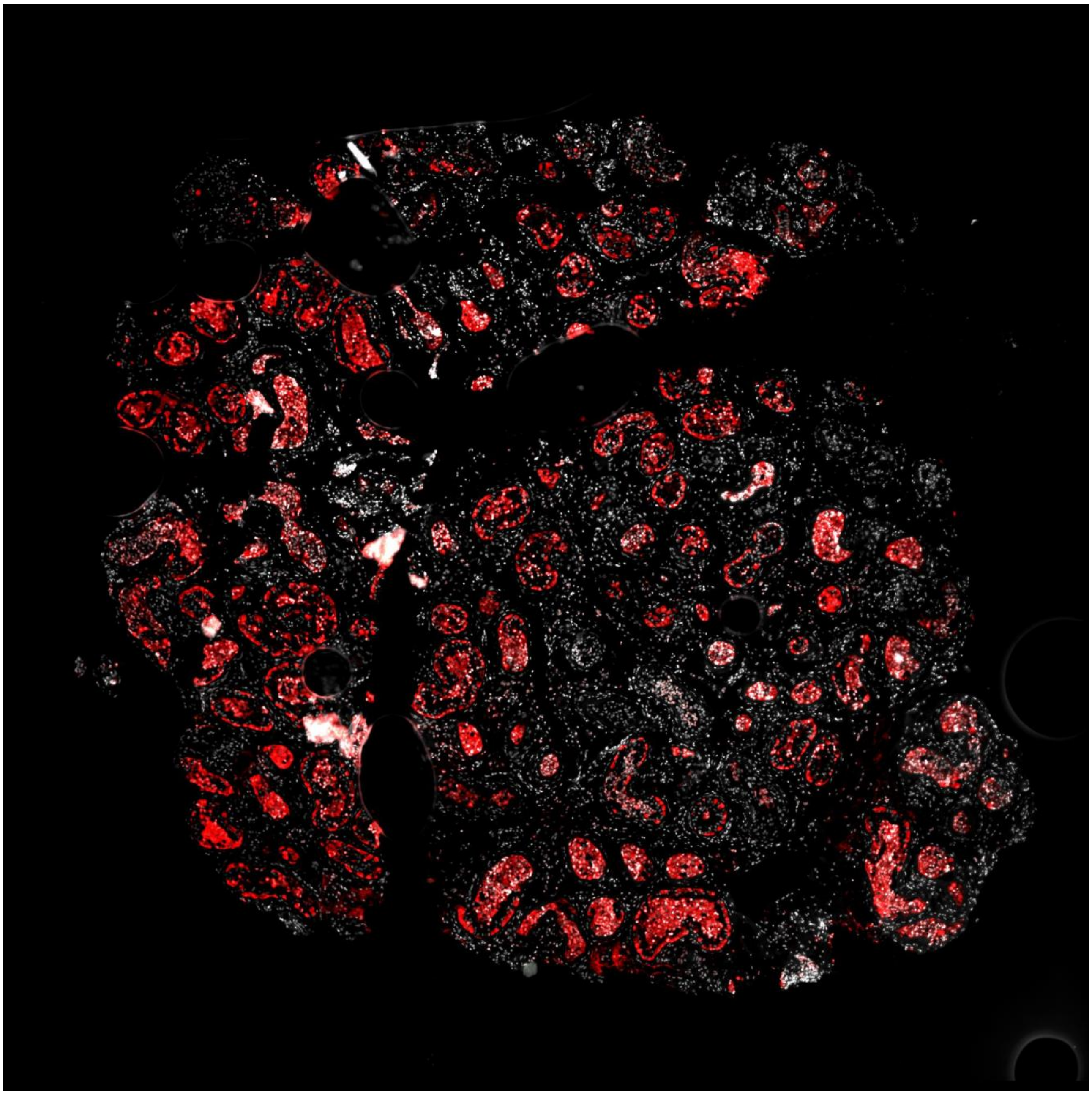

Figure S8B - Human prepubertal testicular tissue stained for nuclei (white), SOX9 (green) and MAGE-A (red) 2023-0248 PCC 26 SC1 1. Secondary antibody control, no SOX9 secondary antibody

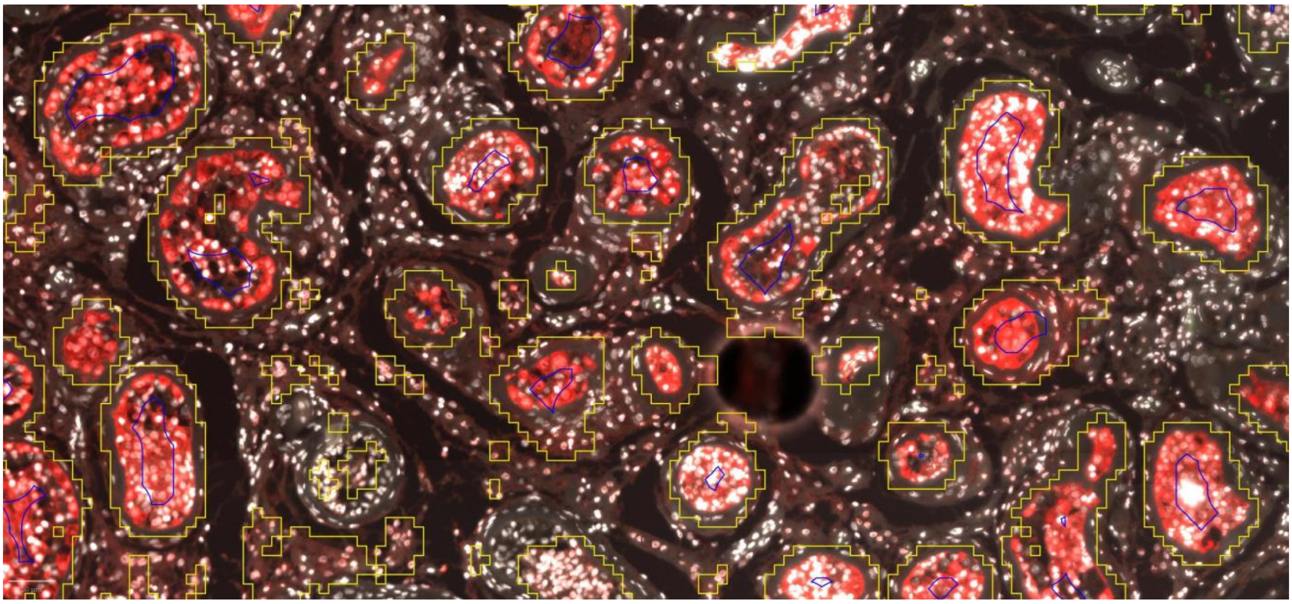

Figure S9A - Human prepubertal testicular tissue stained for nuclei (white), SOX9 (green) and MAGE-A (red). 2023-0248 PCC 30 1

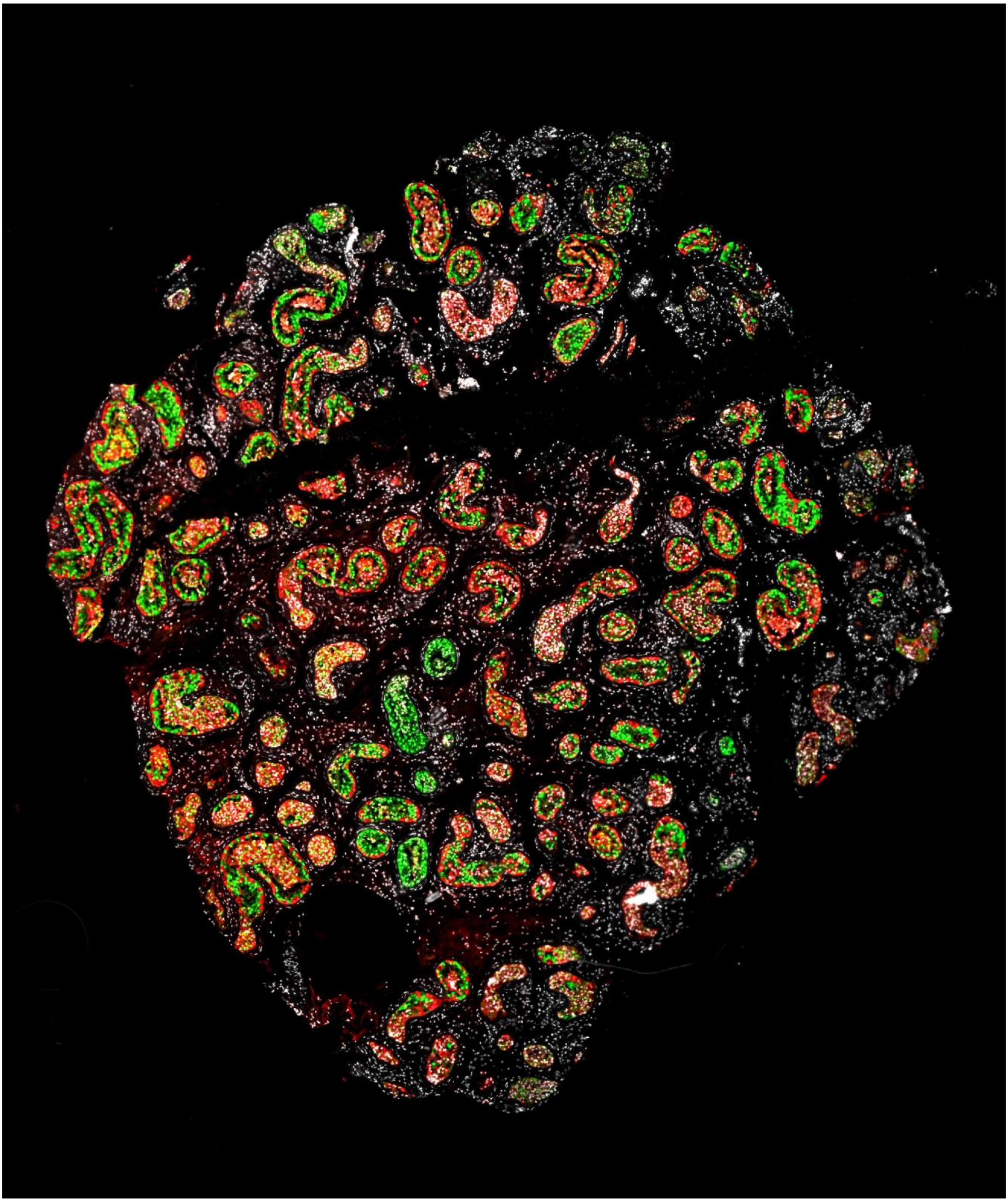

Figure S9B - Human prepubertal testicular tissue stained for nuclei (white), SOX9 (green) and MAGE-A (red). 2023-0248 PCC 30 1

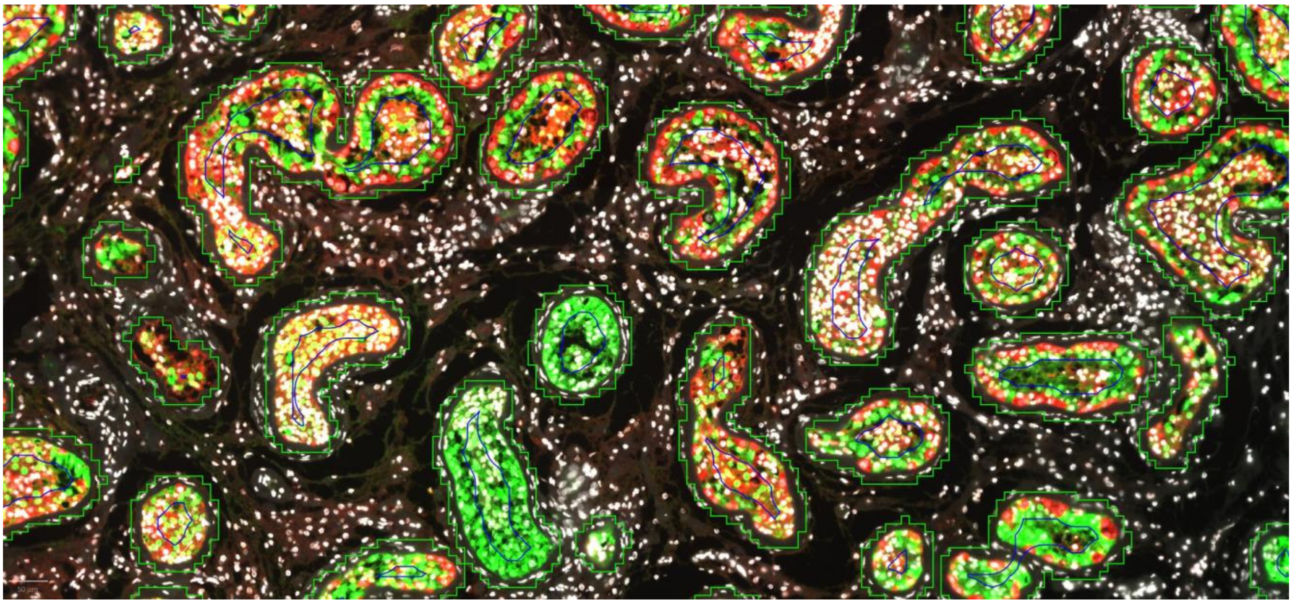

Figure S10A - Human prepubertal testicular tissue stained for nuclei (white), SOX9 (green) and MAGE-A (red). 2023-0248 PCC 34 PC1 1. Primary antibody control, no SOX9 primary antibody

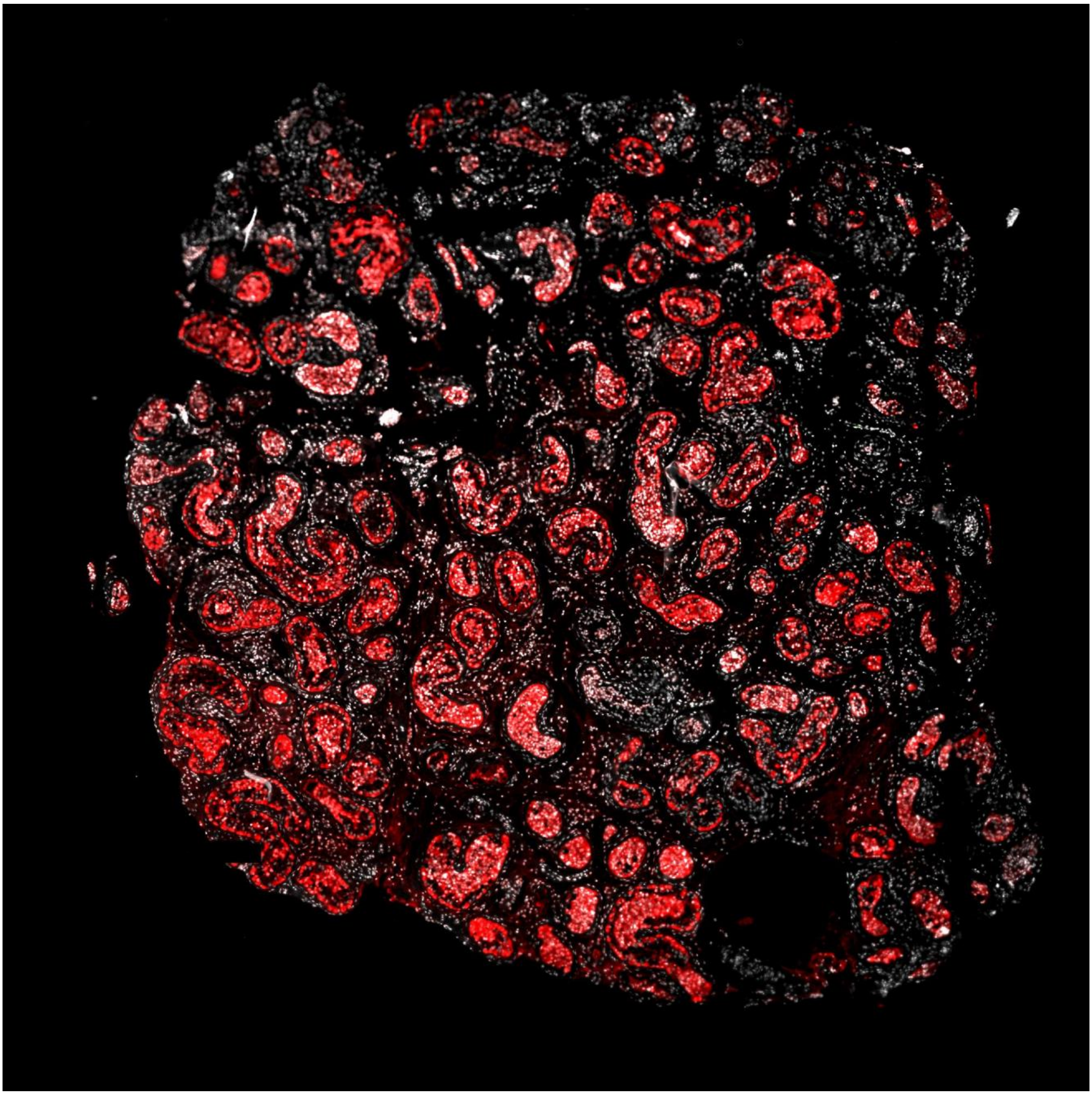

Figure S10B - Human prepubertal testicular tissue stained for nuclei (white), SOX9 (green) and MAGE-A (red). 2023-0248 PCC 34 PC1 1. Primary antibody control, no SOX9 primary antibody

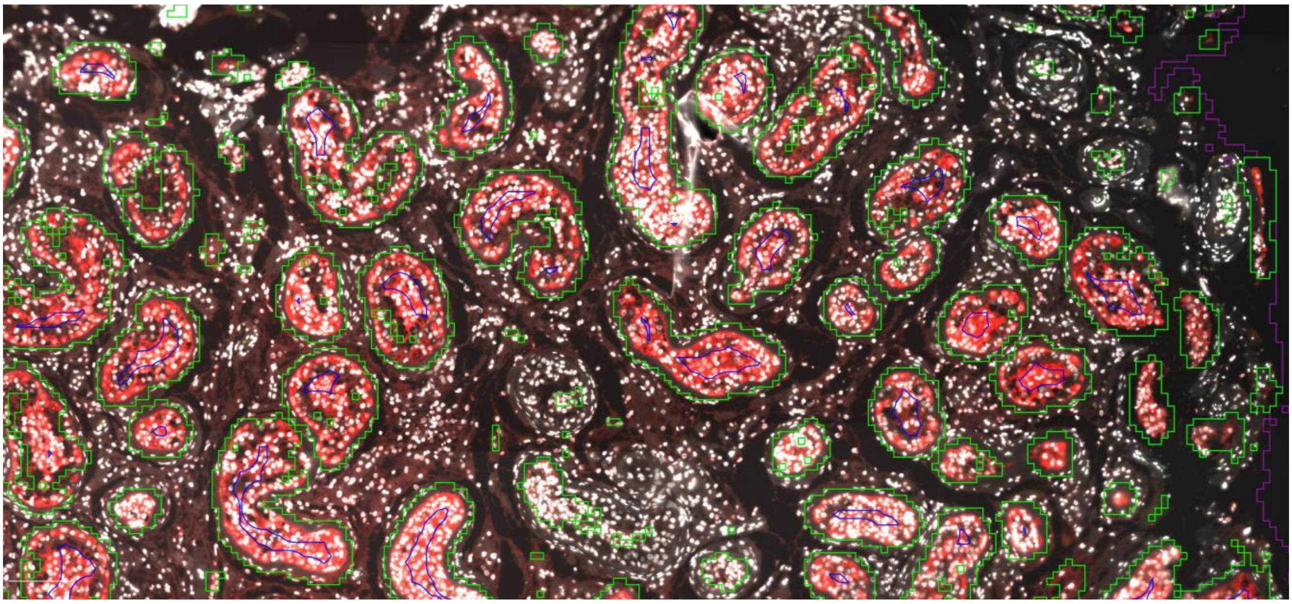

Figure S11A - Human prepubertal testicular tissue stained for nuclei (white), SOX9 (green) and MAGE-A (red). 2023-0248 PCC 35 SC2 2. Secondary antibody control, no MAGE-A secondary antibody

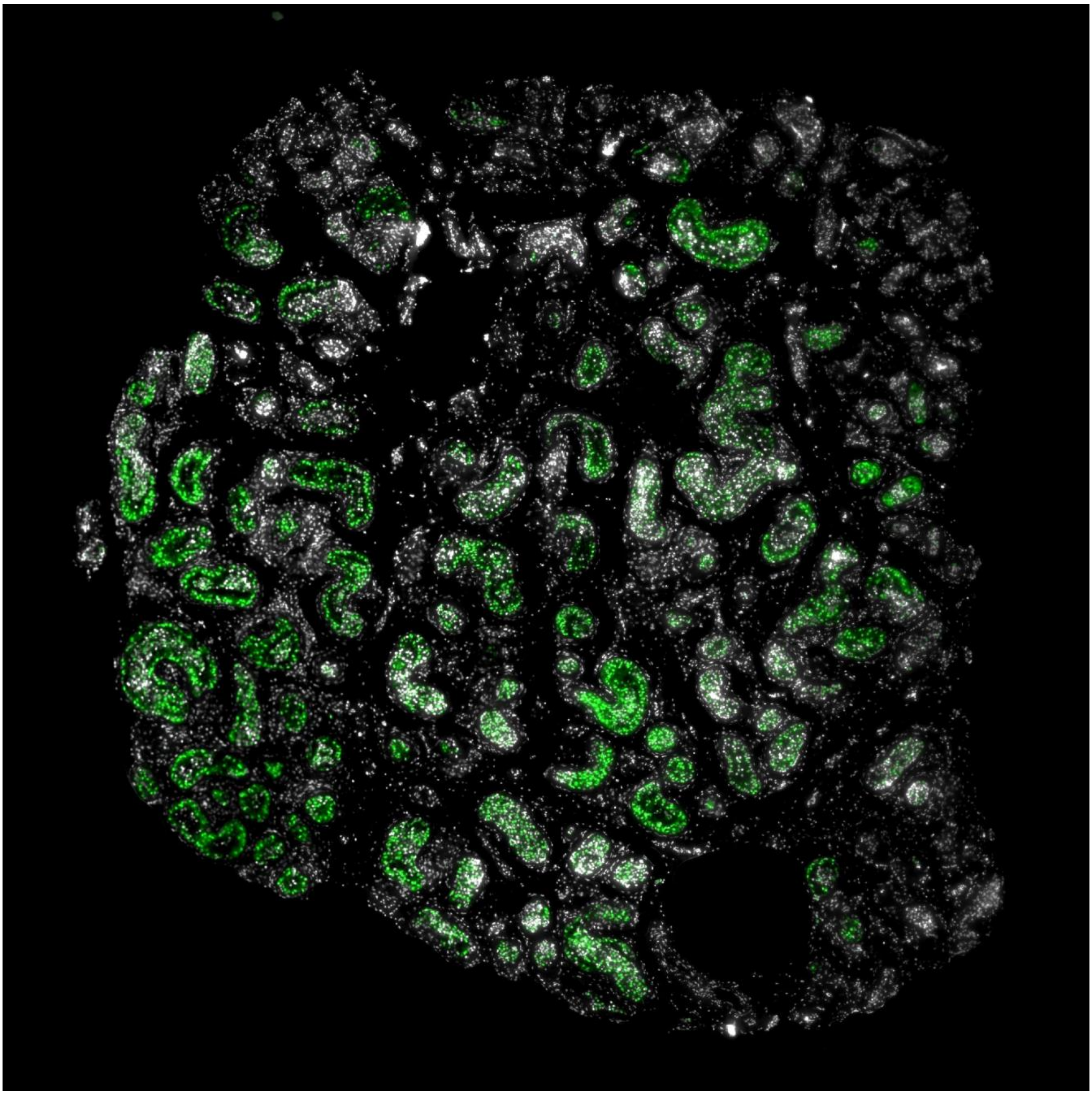

Figure S11B - Human prepubertal testicular tissue stained for nuclei (white), SOX9 (green) and MAGE-A (red). 2023-0248 PCC 35 SC2 2. Secondary antibody control, no MAGE-A secondary antibody

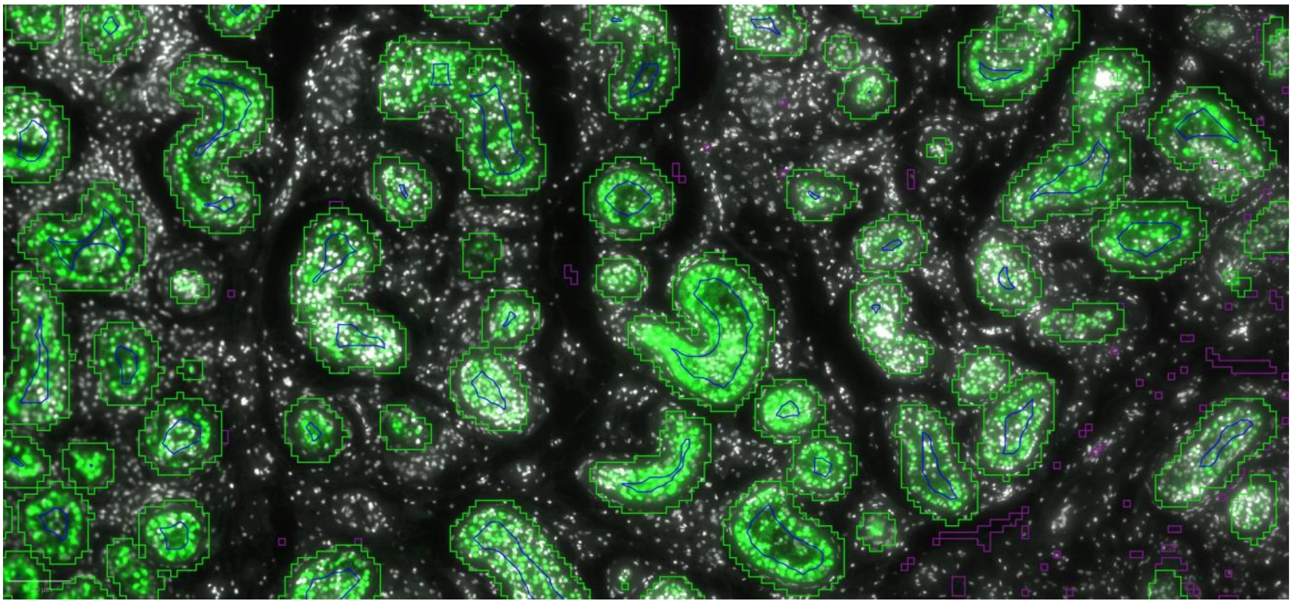

Figure S12A - Human prepubertal testicular tissue stained for nuclei (white), SOX9 (green) and MAGE-A (red). 2023-0248 PCC 35 SC2 3. Secondary antibody control, no MAGE-A secondary antibody

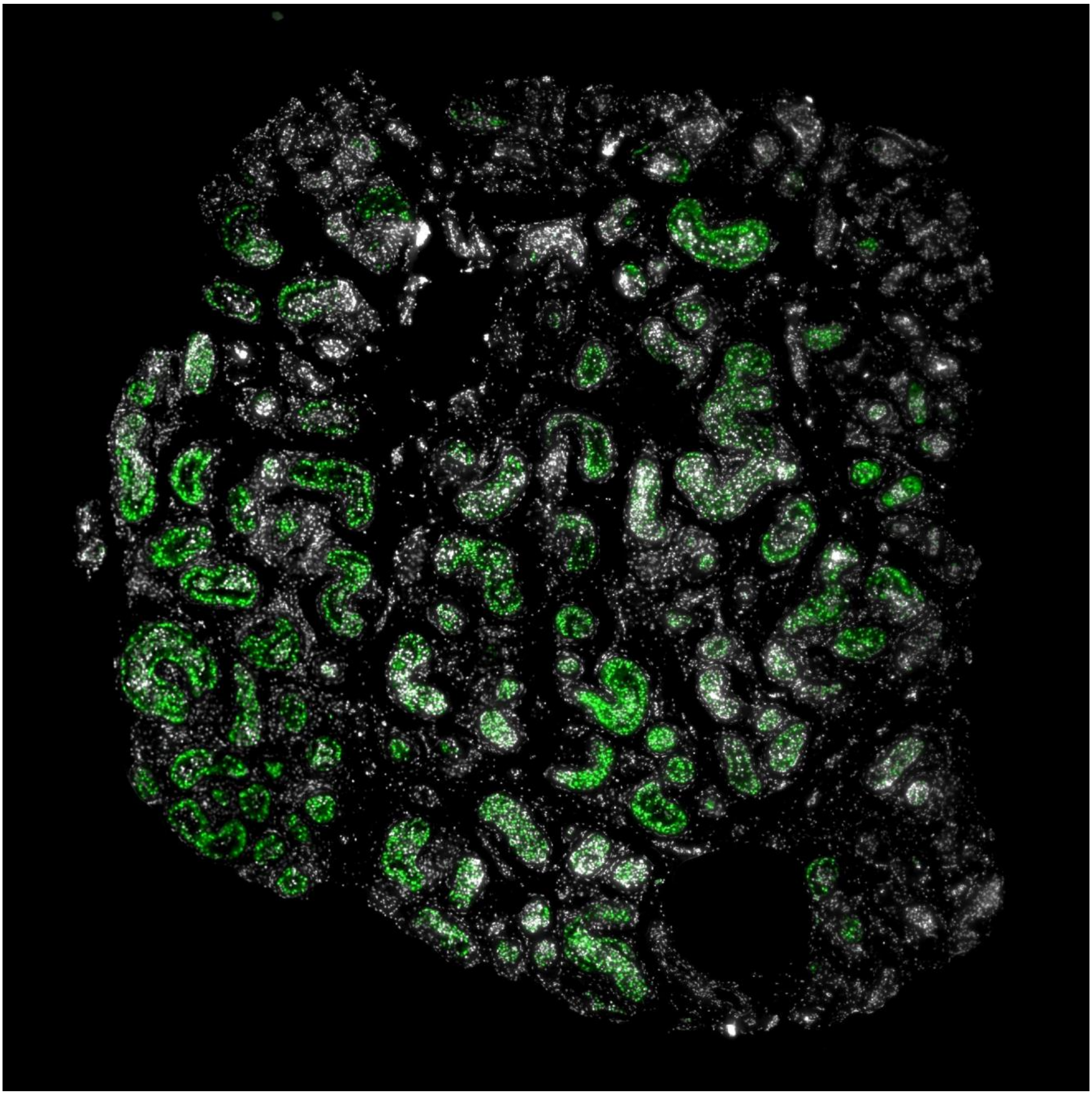

Figure S12B - Human prepubertal testicular tissue stained for nuclei (white), SOX9 (green) and MAGE-A (red). 2023-0248 PCC 35 SC2 3. Secondary antibody control, no MAGE-A secondary antibody

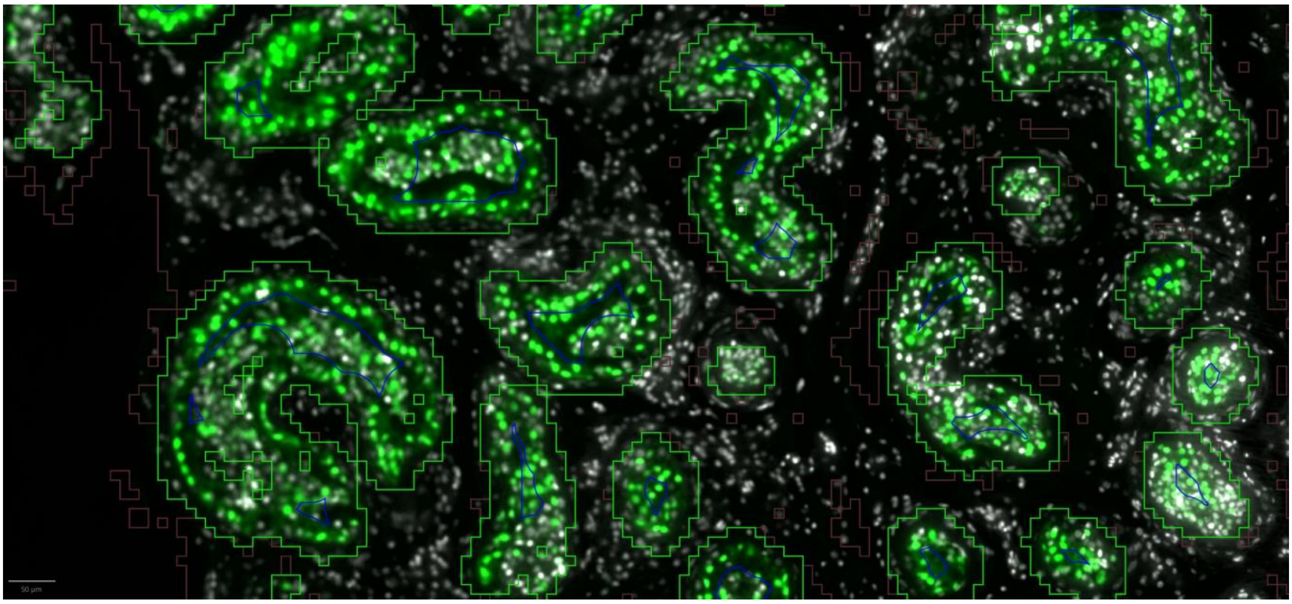

Figure S13A - Human prepubertal testicular tissue stained for nuclei (white), SOX9 (green) and MAGE-A (red). 2023-0248 PCC 39 1

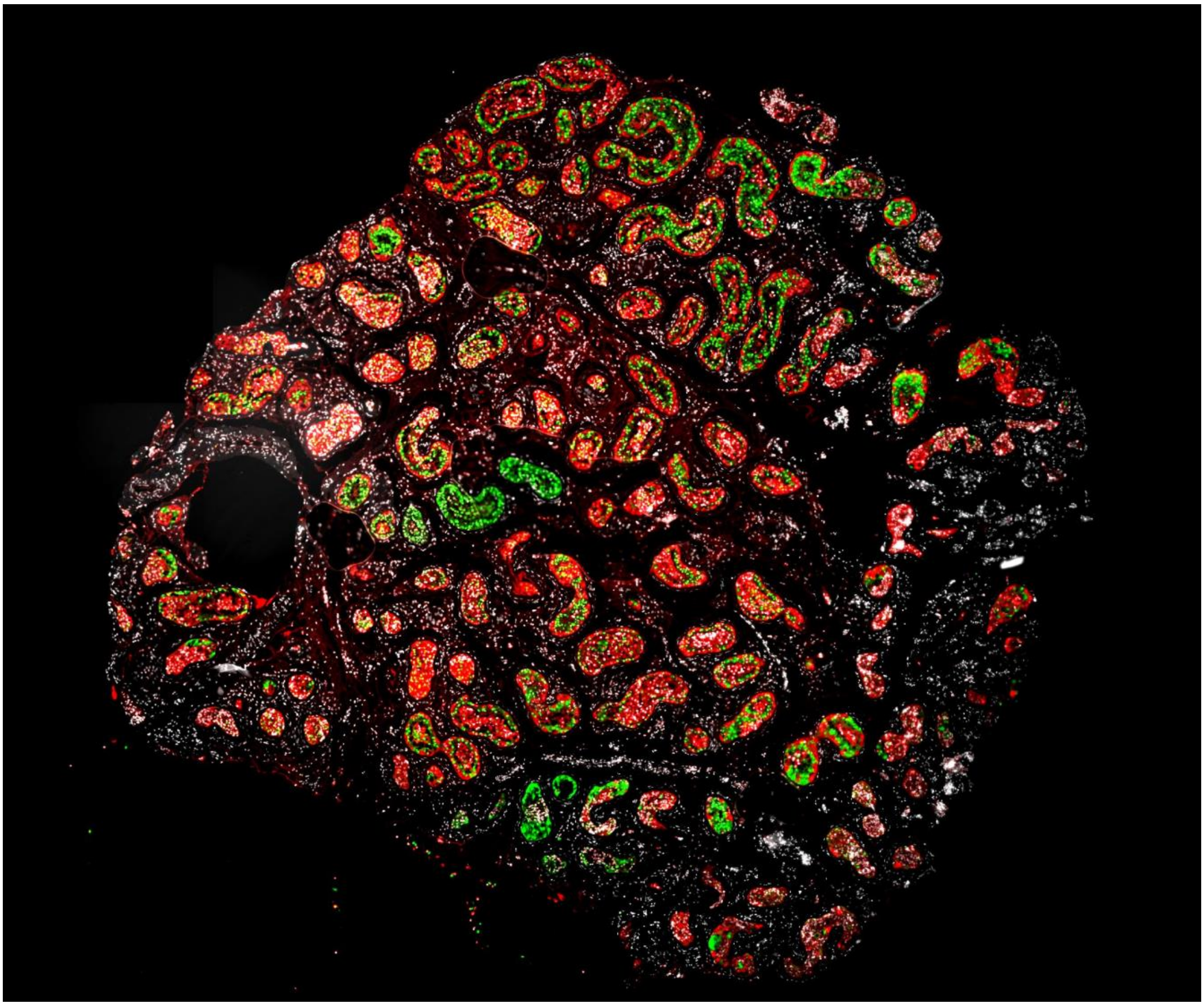

Figure S13B- Human prepubertal testicular tissue stained for nuclei (white), SOX9 (green) and MAGE-A (red). 2023-0248 PCC 39 1

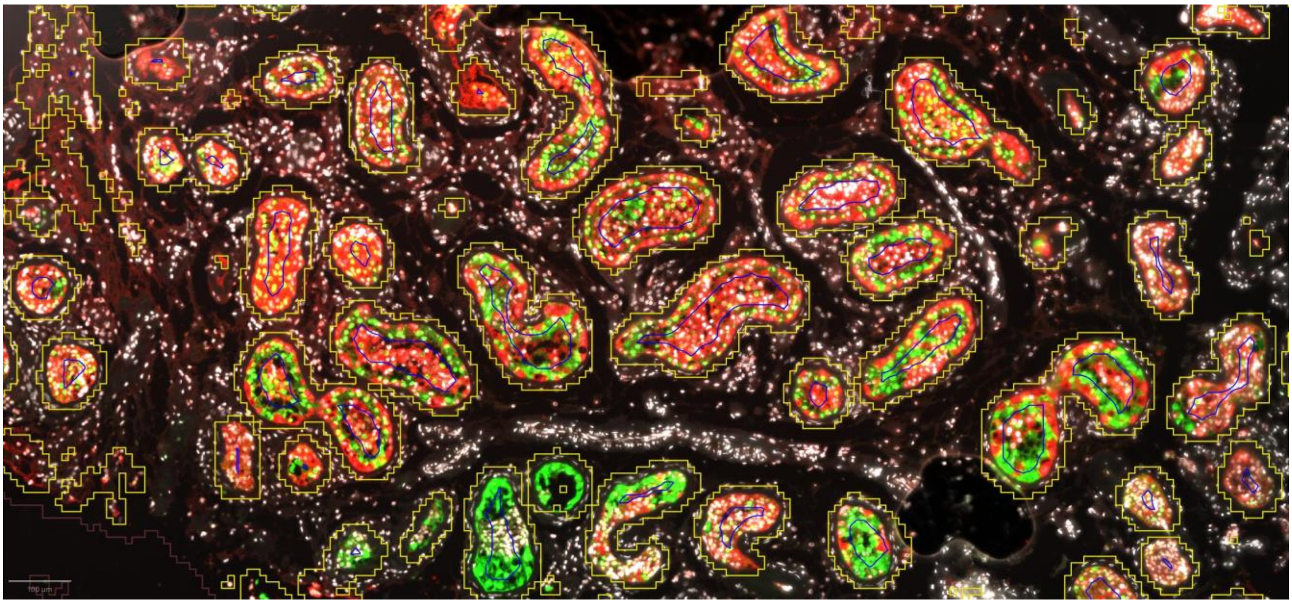

Figure S14A – Mouse testicular tissues stained for nuclei (blue), COUPT-FII (red) and MVH (green) 8185A #2.

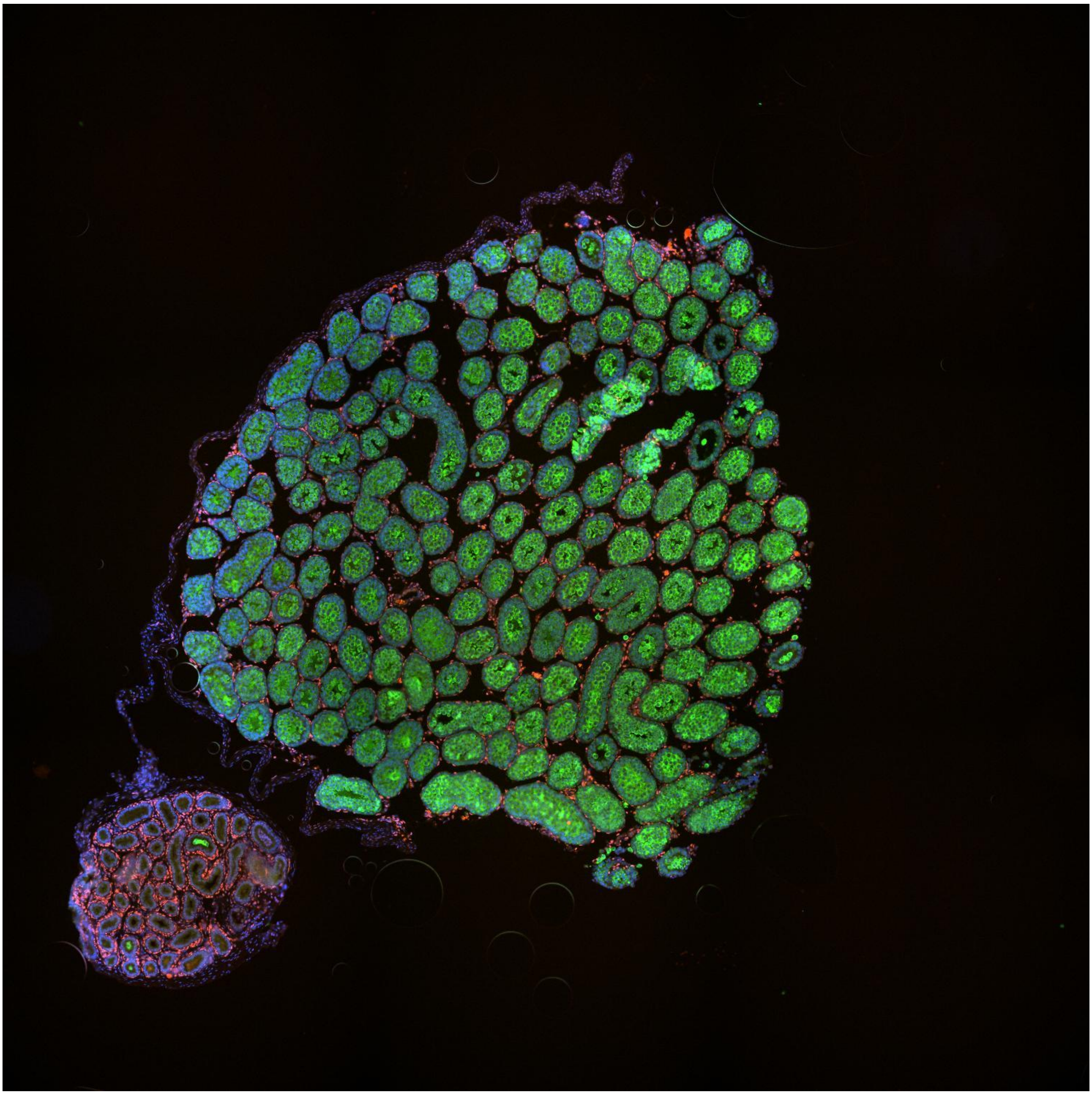

Figure S14B – Segmentation of Mouse testicular tissues stained for nuclei (blue), COUPT-FII (red) and MVH (green) 8185A #2.

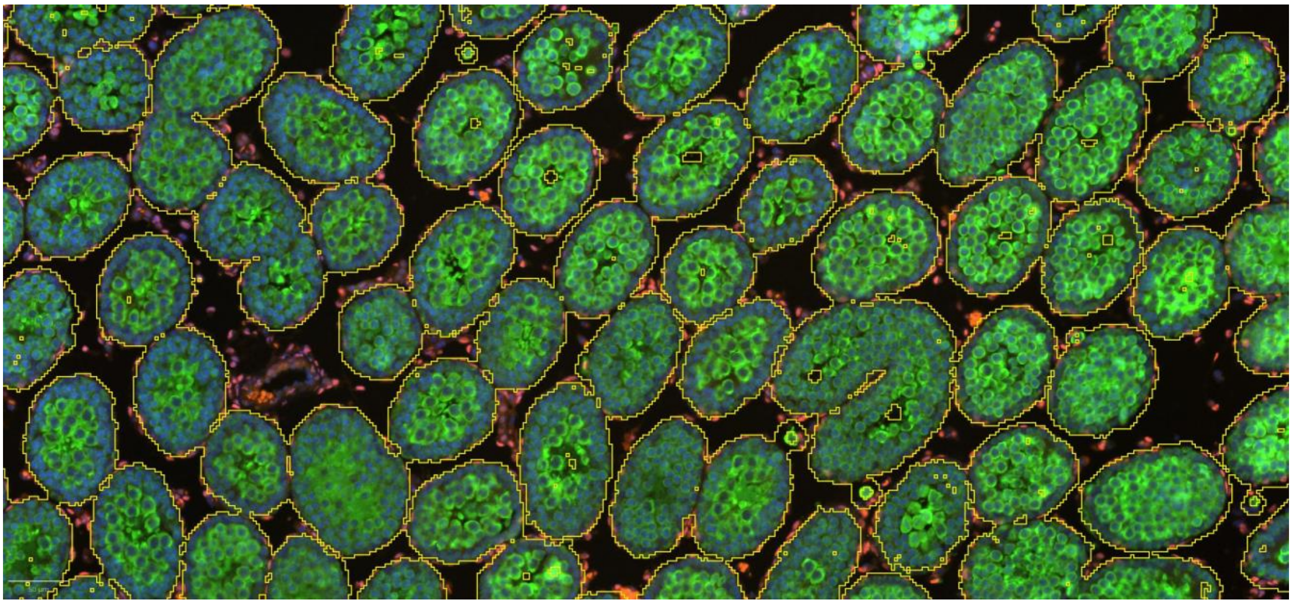

Figure S15A - Mouse testicular tissues stained for nuclei (blue), MVH (orange) and PLZF (green).

8178A #1

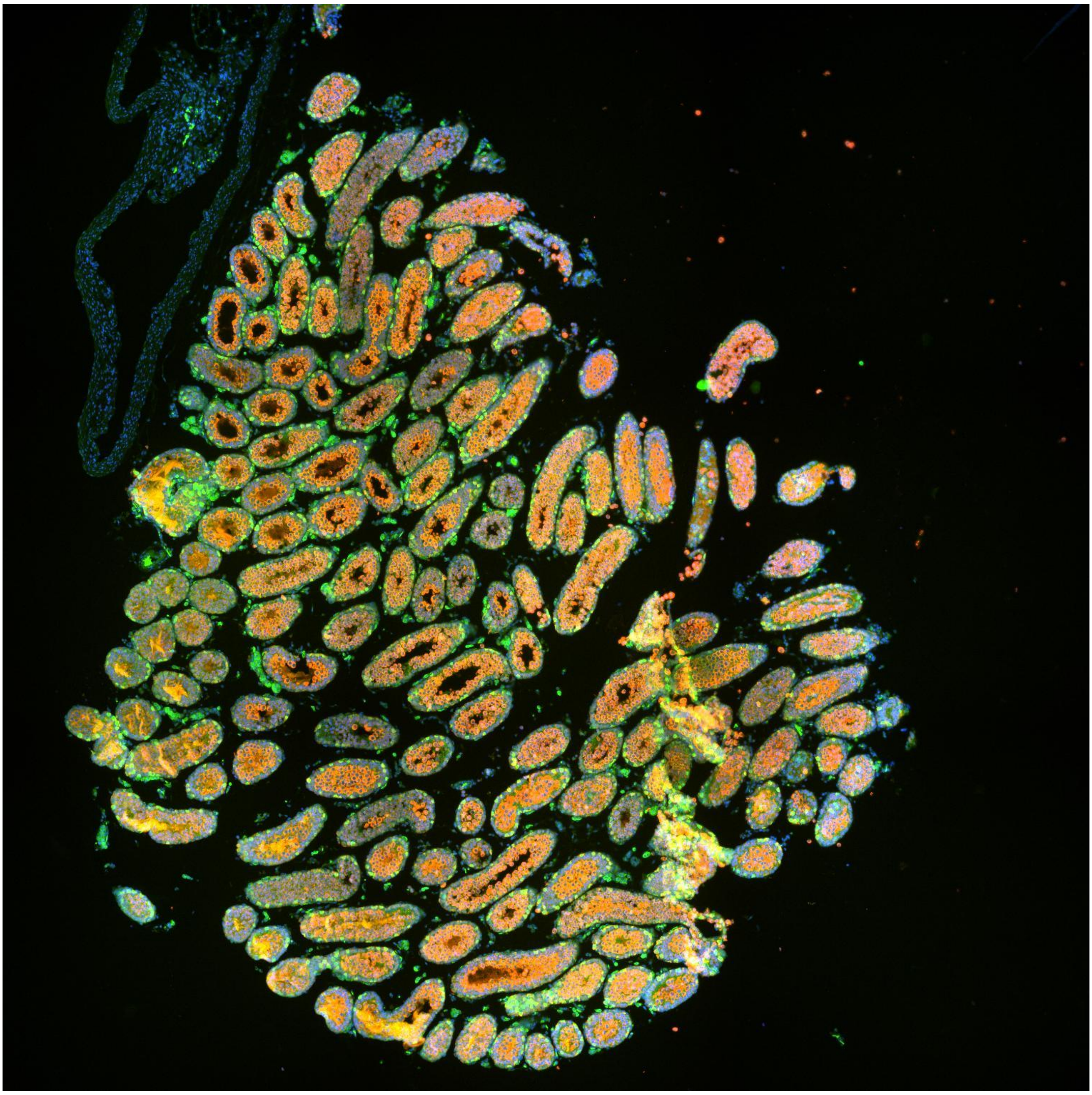

Figure S15B - Mouse testicular tissues stained for nuclei (blue), MVH (orange) and PLZF (green).

8178A #1

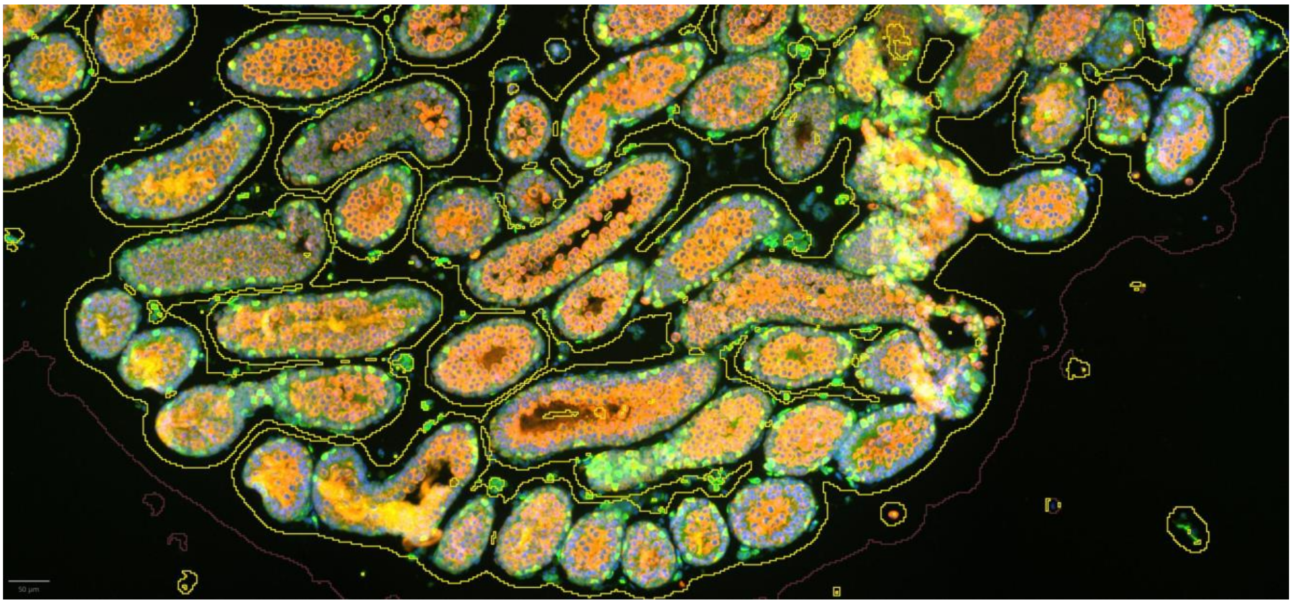

Figure S16 – example of annotations for different ROIs when training the ANN-MLP in QuPath

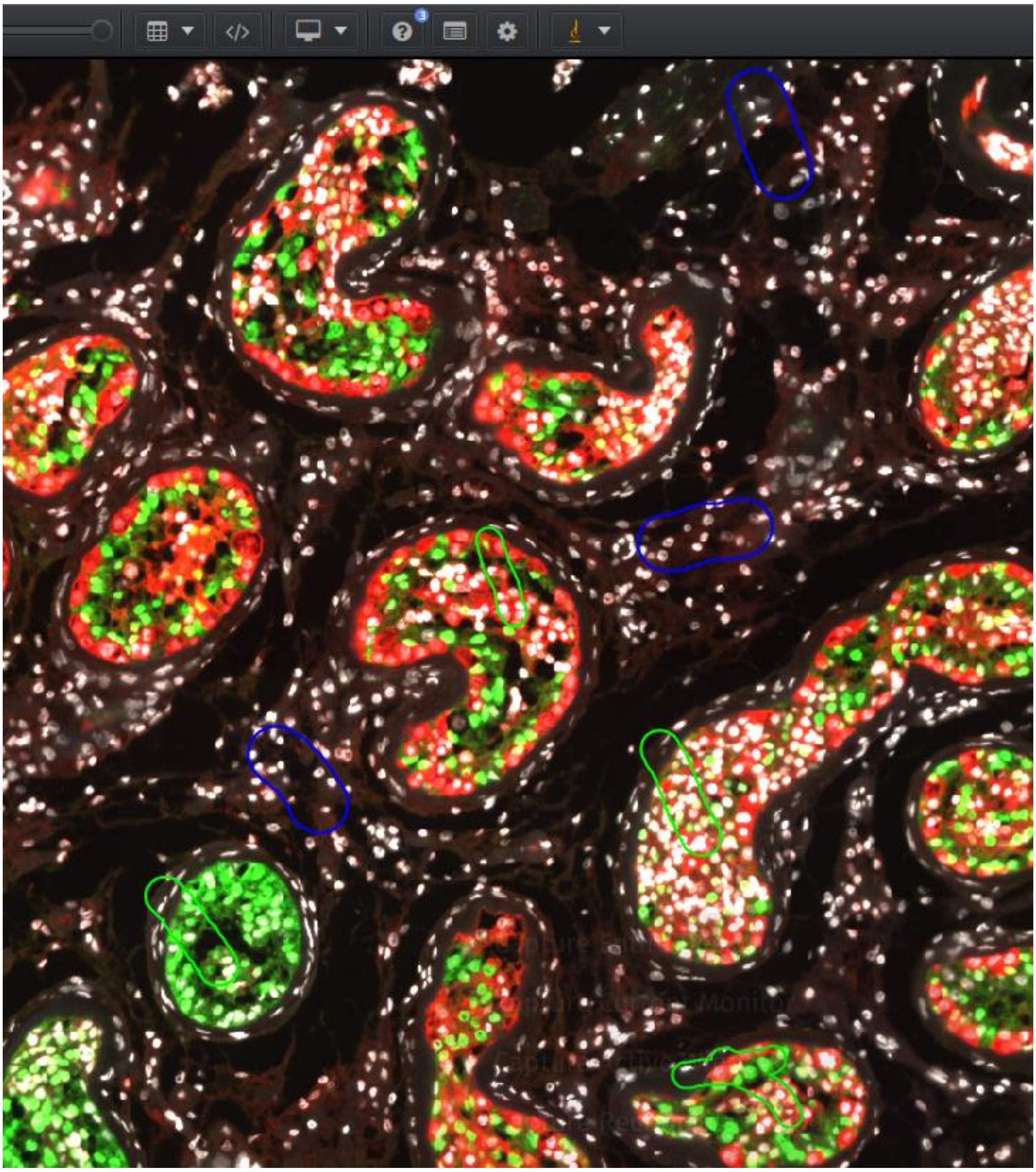

Supplement: Supplementary file 1 [file supplementary_materials_1.pdf]
