# Supplementary material for: Machine learning and automation methods for the segmentation, classification and quantification of testicular tissue sections
Source: Reprod Fertil. 2026 May 29;7(2):RAF250210. doi: 10.1530/RAF-25-0210 (PMC13232597; doi:10.1530/RAF-25-0210)
Supplement: Supplementary file 2 [file supplementary_materials_2.pdf]

QuPath for automated image analysis

Intro

This document will aim to demonstrate how to use a prepared template project in QuPath to directly analyse imuunofluorescent images including segmentation of tubules and quantification/classification of cells. In needed, it will demonstrate how to train a artificial neural network for the segmentation of ROIs such as tubules and and tie into the workflow for cell quantification/classification.

Table of Contents

Intro.......... 1
Preparing QuPath.......... 2
Downloading the QuPath template folder.......... 2
Preparing the QuPath template for the first time.......... 3
Analysing your first project from the QuPath template for segmentation of tubules and quantifying total cell number, SOX9 and MAGE-A4 cells in tubules.......... 4
Exporting data.......... 6
Opening your exported data.......... 7
Training a pixel classifier for segmentation.......... 8
Creating an object classification system.......... 15
Using the template script for image analysis.......... 18
Editing classifier threshold settings.......... 19

Preparing QuPath

1. Download the latest version of QuPath – If using MacOS check what processor you have. Click the apple in the top left of your screen and more info. The CPU will either be Intel or apple (M1, M2, M3 etc).

   1.1 https://qupath.github.io/

2. Once installed run QuPath.

3. The extension manager should launch allowing you to customise QuPath. If it does not open click extensions → manage extension.

4. In the dialogue box we need to install the StarDist plugin

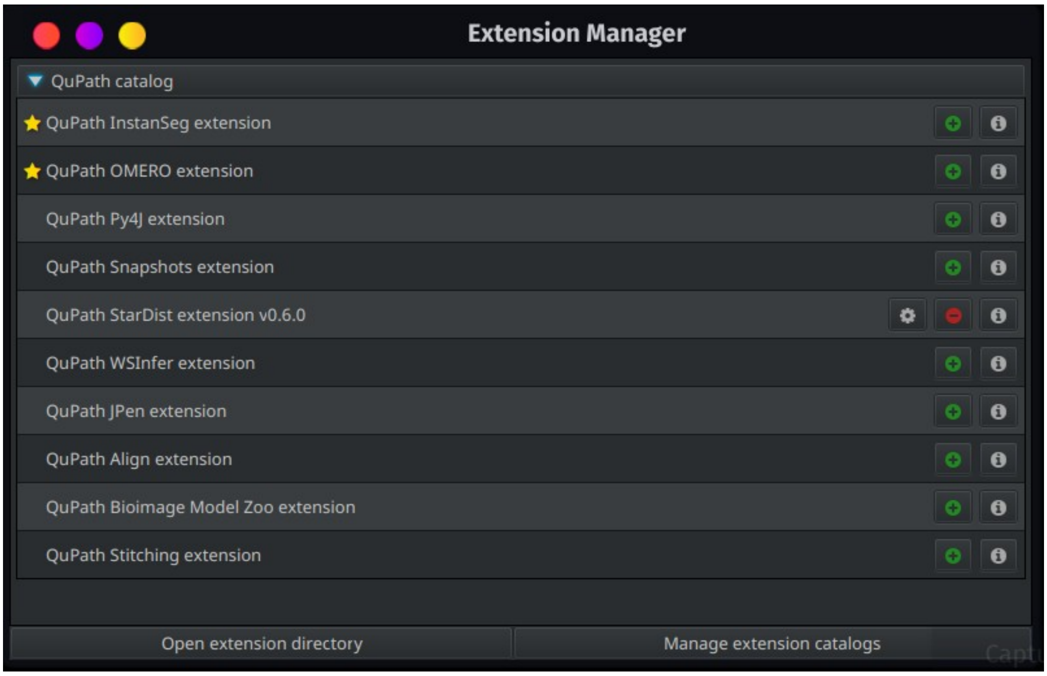

5. Click the Green plus icon to install StarDist. Once installed it should look like the above picture.

   5.1 If prompted 'Where to install – ether chose a location or default' – Choose default – **<u>This is important later!</u>**

6. Once complete close the manager.

Downloading the QuPath template folder.

1. Download the accompanying template folder from the journal called 'QuPath Template'

2. Extract the **<u>whole folder</u>** labelled QuPath template and save it somewhere useful.

   2.1 If using MacOS make sure this is your **<u>home directory</u>** not your documents.

3. Make another copy of the template folder and name it 'Tutorial' (Or something more useful) but we will refer to this as the 'Tutorial' folder from here on out.

4 There should now be a folder in /home/username/Documents/ called QuPath template and another called Tutorial.

Preparing the QuPath template for the first time

1 On your operating system file explorer, open the tutorial folder and browse to the scripts folder.

1.1 In here are some scripts we can use later. However we need to give StarDist a machine learning model to work with. We will use one trained on fluorescent images of nuclei (others are available).

1.2 Go to https://github.com/qupath/models/tree/main/stardist and download dsb2018_heavy_augment.pb (other StarDist models are available)

2 Copy the file ‘dsb2018_heavy_augment.pb’ to the QuPath default directory located at;

2.1 Linux is - /home/username/QuPath

2.2 MacOS is - /users/username/QuPath

2.3 Windows is – c:/Users/username/QuPath

3 Move your file explorer back to the ‘tutorial/scripts’ folder as we will need this later.

4 Back in QuPath, navigate to file → project → open project and navigate to the ‘Tutorial’ folder and open the project.qpproj file.

5 Navigate to Automate → Project scripts → and select the ‘setup’ script.

6 This will open QuPath’s script editor.

7 Browse to line 29 and change the path to where we just copied the StarDist model (dsb2018_heavy_augment.pb). For example on Linux - /home/username/QuPath/model name

```
17
18 import qupath.ext.stardist.StarDist2D
19 import qupath.lib.scripting.QP
20
21 // IMPORTANT! Replace this with the path to your StarDist model
22 // that takes a single channel as input (e.g. dsb2018_heavy_augment.pb)
23 // You can find some at https://github.com/qupath/models
24 // (Check credit & reuse info before downloading)
25 def modelPath = "/home/adam/QuPath/dsb2018_heavy_augment.pb"
26
27 // Customize how the StarDist detection should be applied
28 // Here some reasonable default options are specified
29 def stardist = StarDist2D
```

8 In the script editor, navigate to file → save.

9 Back in your operating system file explorer (which should be in the tutorial folder/scripts), copy the setup script and place a copy in your template folder/scripts.

10 Rename this script to tubules.groovy

11 The next time you make a copy of your template folder, you no longer need to configure StarDist.

12 The Template folder now an empty project that contains some scripts we can use.

13 Close QuPath and delete the ‘tutorial’ folder as we no longer need it.

14 Make another copy of the template folder and rename it ‘my first experiment’ or something useful relating to the project. This guide will refer to this folder as ‘my first experiment’ from here.

Analysing your first project from the QuPath template: Segmentation of tubules and quantifying total cell number, SOX9 and MAGE-A4 cells in tubules

If you have setup the template folder according the section ‘Preparing the QuPath template for the first time’ then continue, if not **read this section.**

1 Open QuPath and in file → project → open project browse to your new ‘my first experiment’ folder and open the project.qpproj file.

2 This is an empty project where we can add some images to analyse

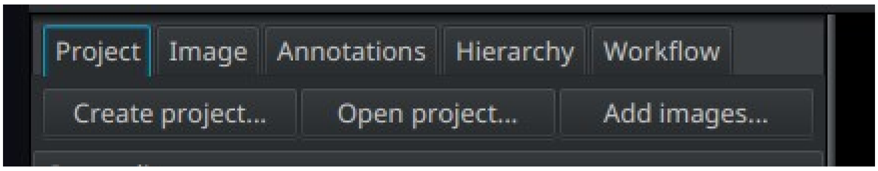

3 Click the ‘Add images’ button in the navigation panel on the left of the screen.

4 In the dialogue box select the image type and change to ‘Fluorescence’

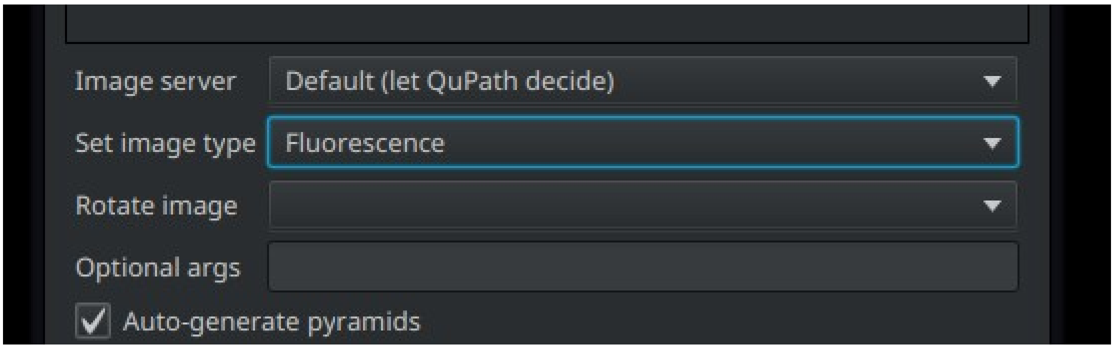

5 Click the import files button and browse to the file you wish to analyse.

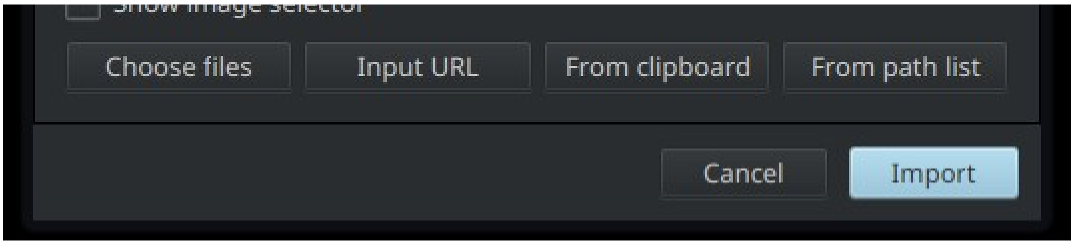

6 Check the file list is complete.

7 Then press import button.

8 A list of file will now appear in the navigation panel. We will analyse all the images in the same manner.

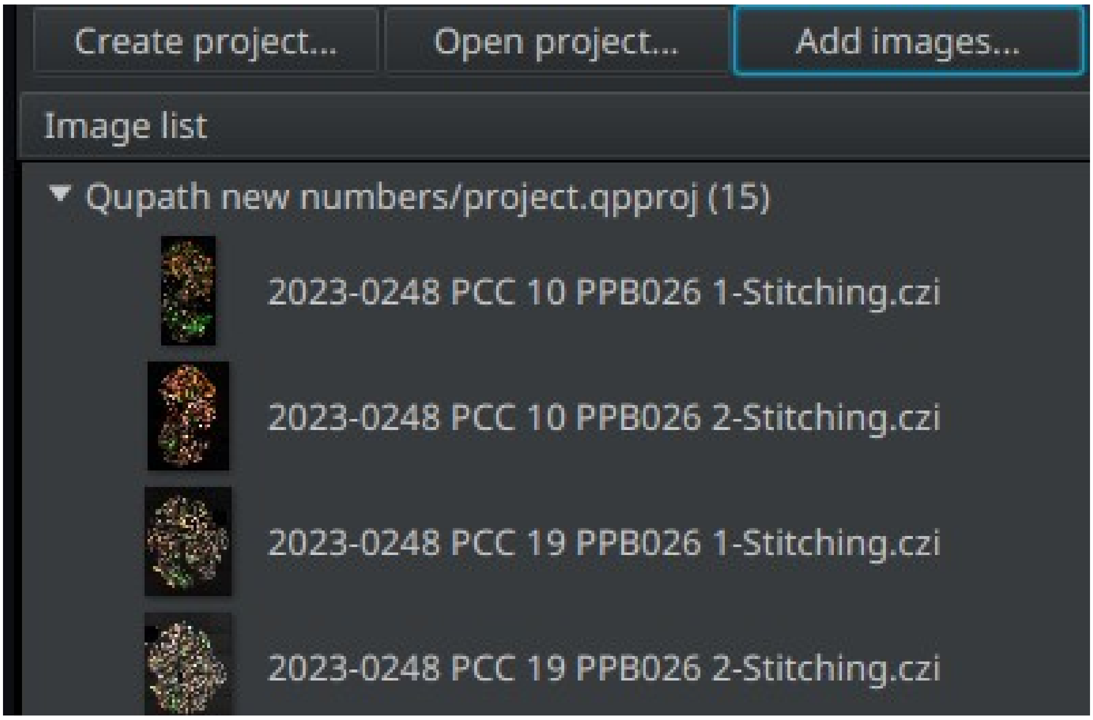

9 Go to Automate → project scripts and open the ‘Tubule’groovy’ script we customised earlier.

10 On the bottom right side of the script editor, click the ellipses and then ‘Run for project’. This will then run the script for the all of the selected images in the project.

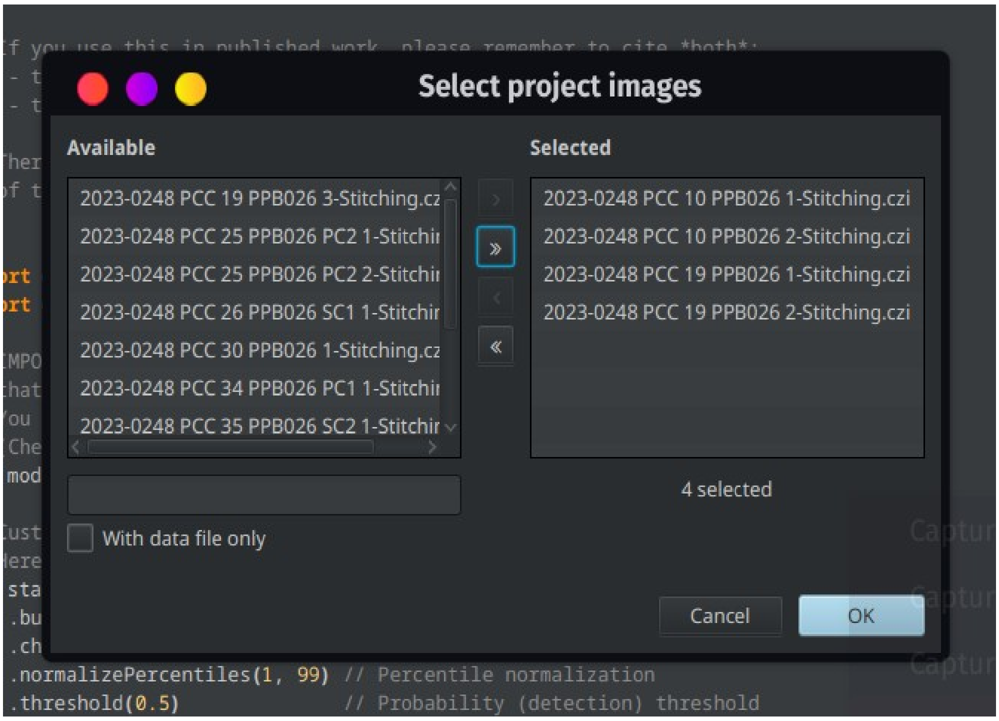

11 On the next dialogue window select the images you want to analyse. Click the double arrow to move all of the images to the right side. When happy, click OK to start the analysis.

12 QuPath will now move though each of the images segmenting the tubules and applying an object classifier.

In the likely event that either the segmentation does not work/ is not up to the correct standard or the classification of cell is not correct please see the corresponding sections; Training a pixel classifier for segmentation or Creating an object classification system for details on how to create your own classification and segmentation systems.

Exporting data

After the script has finished running we need to export the data.

1 Go to measure → export measurements

2 the following dialogue box will appear

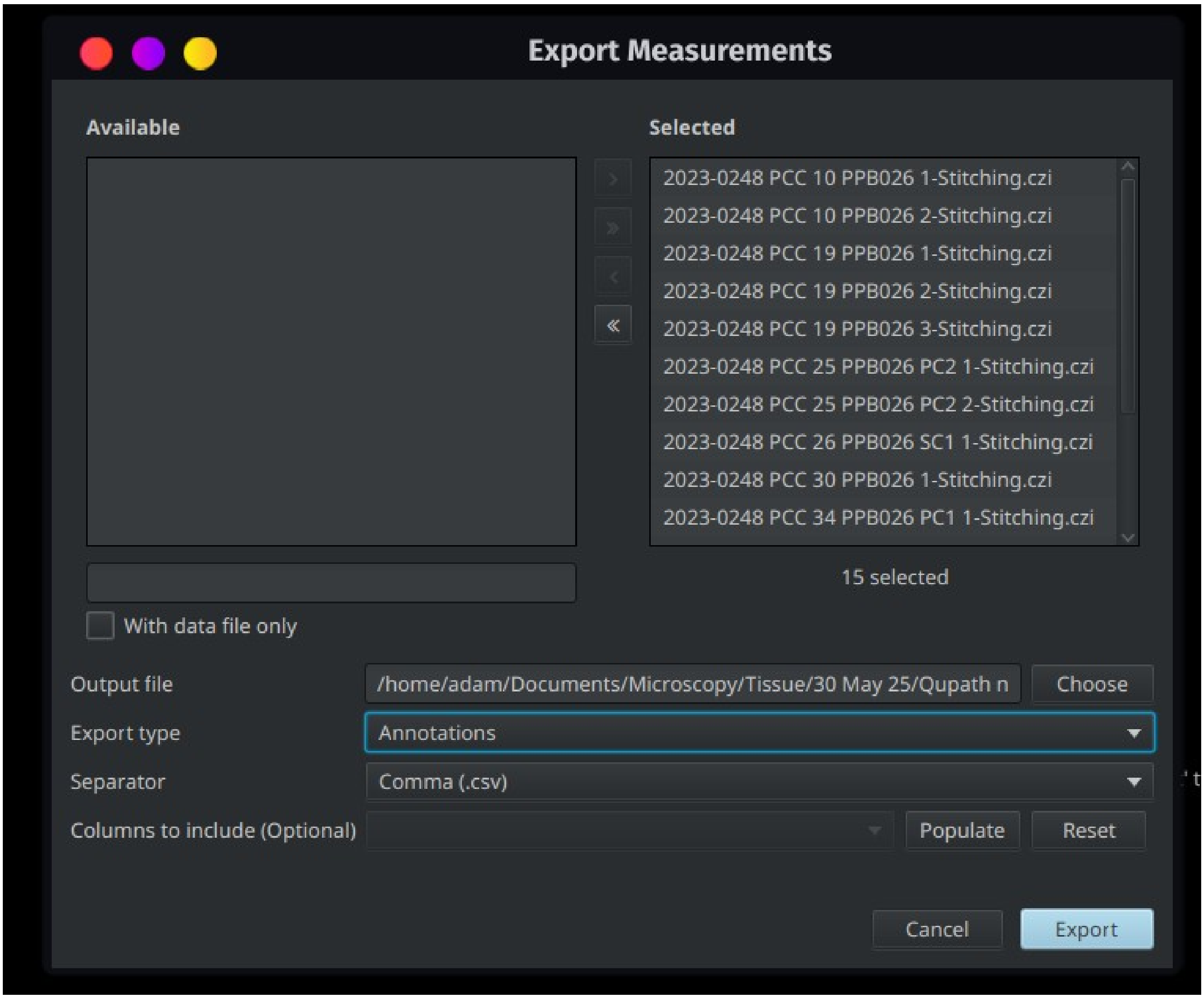

3 Again, move the images to the right side to export the data. Use the double arrow to move all the images which will by likely.

4 Next on the drop down box ‘Separator’ select comma (.CSV)

5 Next click choose to select where to save the csv file, for example /home/username/my first experiment/my first experiment.csv .

6 Finally in the ‘Export type’ select Annotations

7 When you are happy with the selections, click export.

Opening your exported data

1 Go to the location you saved your e.g /home/username/my first experiment/ and click to open my first experiment.csv.

1.1 We recommend using LibreOffice https://www.libreoffice.org/download/download-libreoffice/ for opening CSV due to the ease of use of the importer.

2 Click the CSV to open and when prompted select the separator options as ‘separated by’ and tick the comma box. A preview is displayed. Don't worry if you select the wrong options, close the program and start again. No settings are permanent.

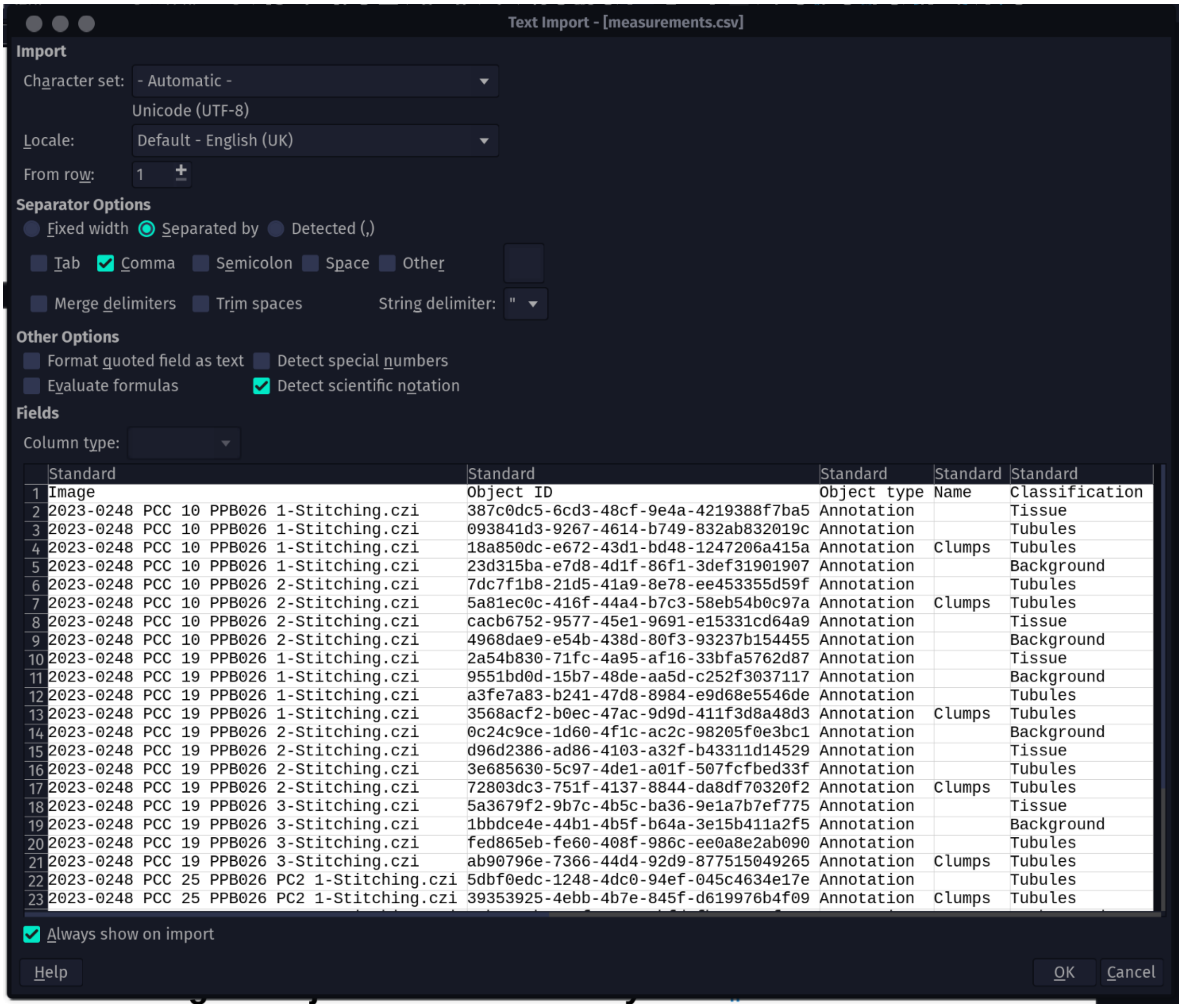

3 This will open your data in an spreadsheet format.

	Image	Object ID	Object type	Name	Classification	Parent	ROI	Centroid X µm	Centroid Y µm	Num Detections	Num AF488	Num AF647	Num AF647: AF488	Area µm^2	Perimeter µm
2	2023-0248 PCC 10 PPB026 1-Stitching.czi	387c0dc5-6cd3-48cf-9e4a-4219388f7ba5	Annotation		Tissue	Root object (Image)	Geometry	583.55	1316.7	0	0	0	0	1454535.7	57283.2
3	2023-0248 PCC 10 PPB026 1-Stitching.czi	093841d3-9267-4614-b749-832ab832019c	Annotation		Tubules	Root object (Image)	Geometry	678.08	1206.5	6933	620	468	1	1106368.6	44969.6
4	2023-0248 PCC 10 PPB026 1-Stitching.czi	18a850dc-e672-43d1-bd48-1247206a415a	Annotation	Clumps	Tubules	Annotation (Tubules)	Geometry	744.72	1259.3	1768	192	100	1	245666.3	16037.8
5	2023-0248 PCC 10 PPB026 1-Stitching.czi	23d315ba-e7d8-4d1f-86f1-3def31901907	Annotation		Background	Root object (Image)	Geometry	756.61	1290.1	0	0	0	0	777994.9	17326.4
6	2023-0248 PCC 10 PPB026 2-Stitching.czi	7dc7f1b8-21d5-41a9-8e78-ee453355d59f	Annotation		Tubules	Root object (Image)	Geometry	961.61	1156.6	8767	1102	1649	117	1100960.6	52166.4
7	2023-0248 PCC 10 PPB026 2-Stitching.czi	5a81ec0c-416f-44a4-b7c3-58eb54b0c97a	Annotation	Clumps	Tubules	Annotation (Tubules)	Geometry	999.53	938.36	1172	197	388	29	122262.8	11990.6
8	2023-0248 PCC 10 PPB026 2-Stitching.czi	cacb6752-9577-45e1-9691-e15331cd64a9	Annotation		Tissue	Root object (Image)	Geometry	927.22	1274.8	1	0	0	0	1766793.6	69846.4
9	2023-0248 PCC 10 PPB026 2-Stitching.czi	4968dae9-e54b-438d-80f3-93237b154455	Annotation		Background	Root object (Image)	Geometry	1015.9	1346.9	0	0	0	0	2107822.1	26291.2
10	2023-0248 PCC 19 PPB026 1-Stitching.czi	2a54b830-71fc-4a95-af16-33bfa5762d87	Annotation		Tissue	Root object (Image)	Geometry	1203.9	1588.9	1	0	0	0	2487355.5	125486.4
11	2023-0248 PCC 19 PPB026 1-Stitching.czi	9551bd0d-15b7-48de-aa5d-c252f3037117	Annotation		Background	Root object (Image)	Geometry	1367.4	1592.4	0	0	0	0	3937240.3	87360

4 Select, rearrange and copy the required data into graphpad prism.

	Num Detections	Num AF488	Num AF647	Num AF647: AF488	Area µm^2	Perimeter µm
7	0	0	0	0	1454535.7	57283.2
5	6933	620	468	1	1106368.6	44969.6
3	1768	192	100	1	245666.3	16037.8
1	0	0	0	0	777994.9	17326.4
6	8767	1102	1649	117	1100960.6	52166.4
6	1172	197	388	29	122262.8	11990.6
8	1	0	0	0	1766793.6	69846.4

4.1 Useful information is going to be the area of tubules in this example column N, number of SOX9 cells – column K and number of MAGE-A4 cells column L.

Training a pixel classifier for segmentation

In the likely event that you do not want to use (or cannot use) the pixel classifier included in the 'Template' folder you can train your own to detect and annotate regions of interest.

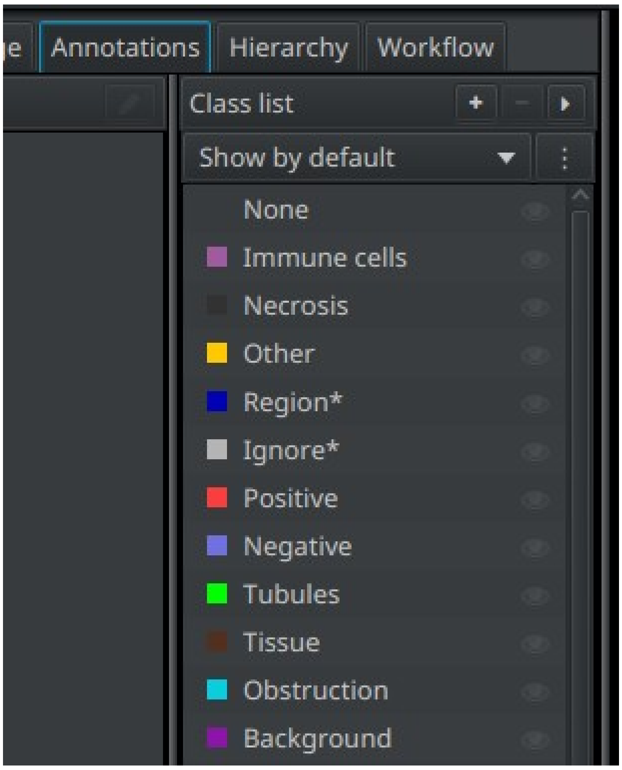

1 First create the classification that you wish the pixel classifier to detect.

1.1 Go to the annotation tab, Click the plus sign and in the dialogue box choose a name for the ROI such a 'Tubules', 'Tissue' etc etc.

2 Once you have added all the regions you want to train, annotate the training images with the regions.

3 Each image should have 3 small annotations for reach ROI. For example 3 x tubule annotations, 3 x tissue annotation and 3 x background annotations

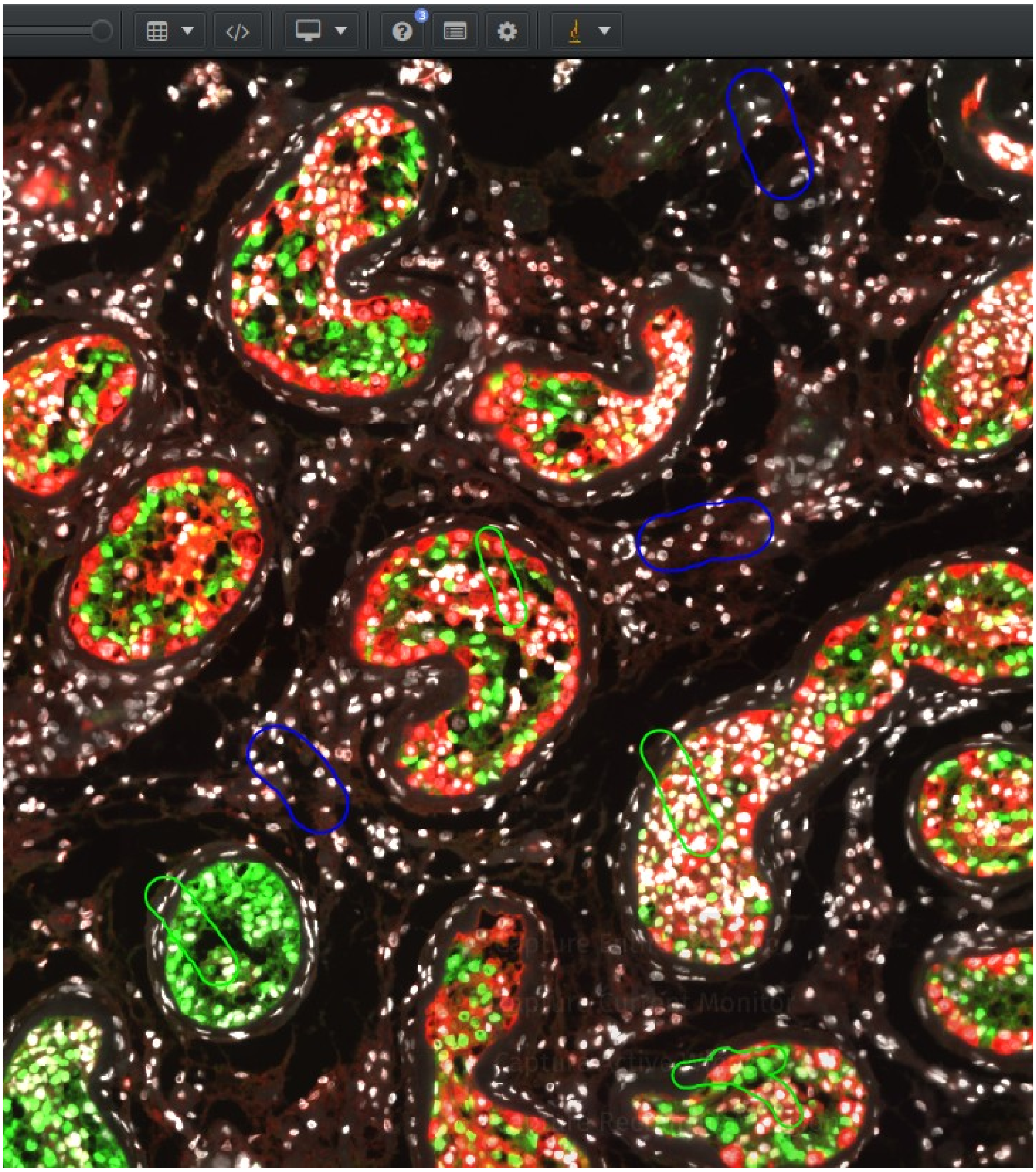

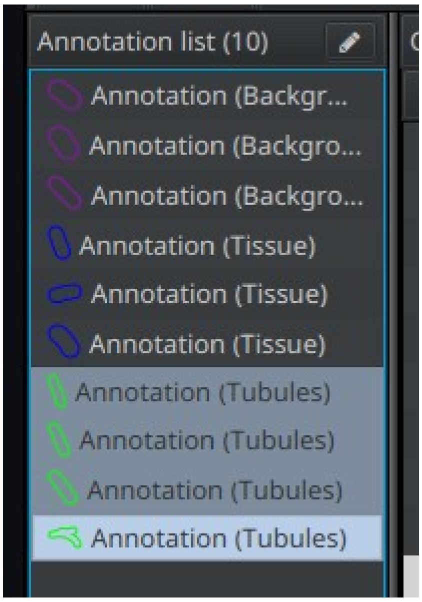

4 Assign the annotations to the correct classification by selecting multiple annotations and clicking the pen in the top corner

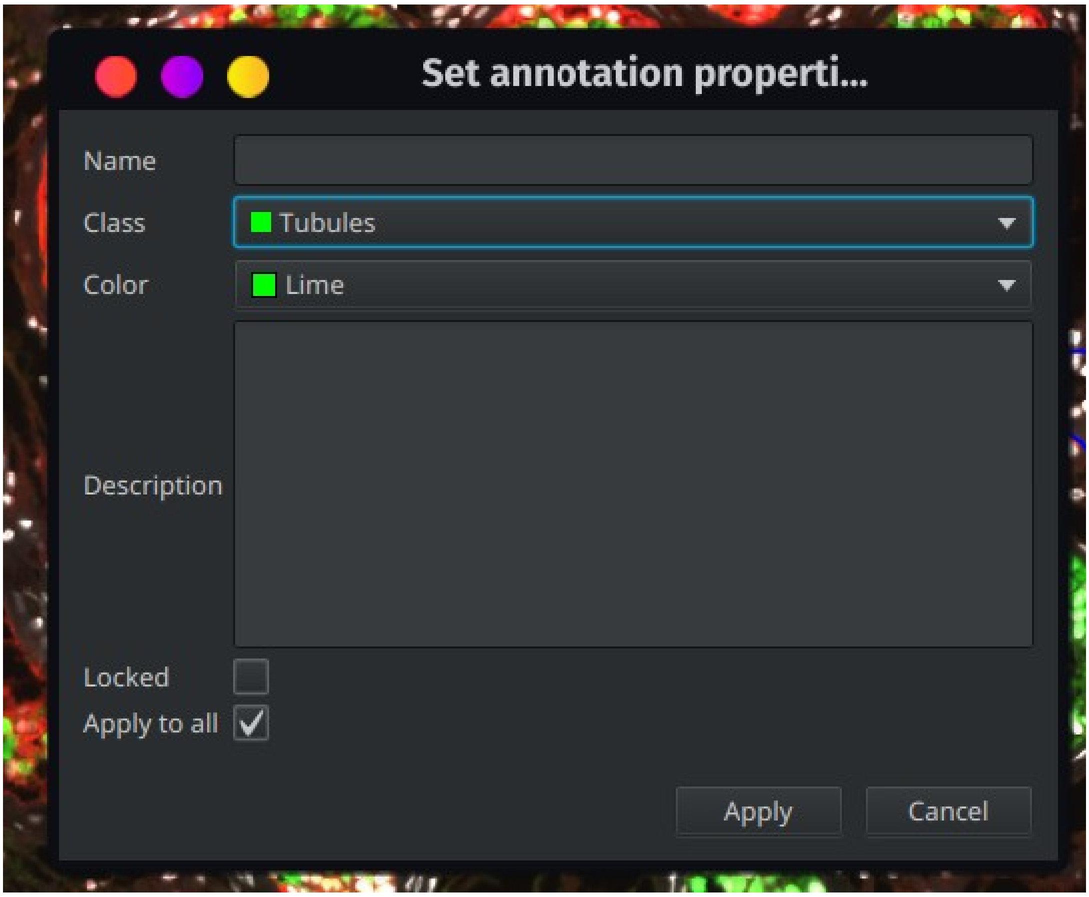

5 In the dialogue box, selected the corresponding class for the ROIs in this example a tubule.

6 Once you have annotated your training images, we can use them to train a pixel classifier.

7 Navigate to classify → pixel classification → train pixel classifier

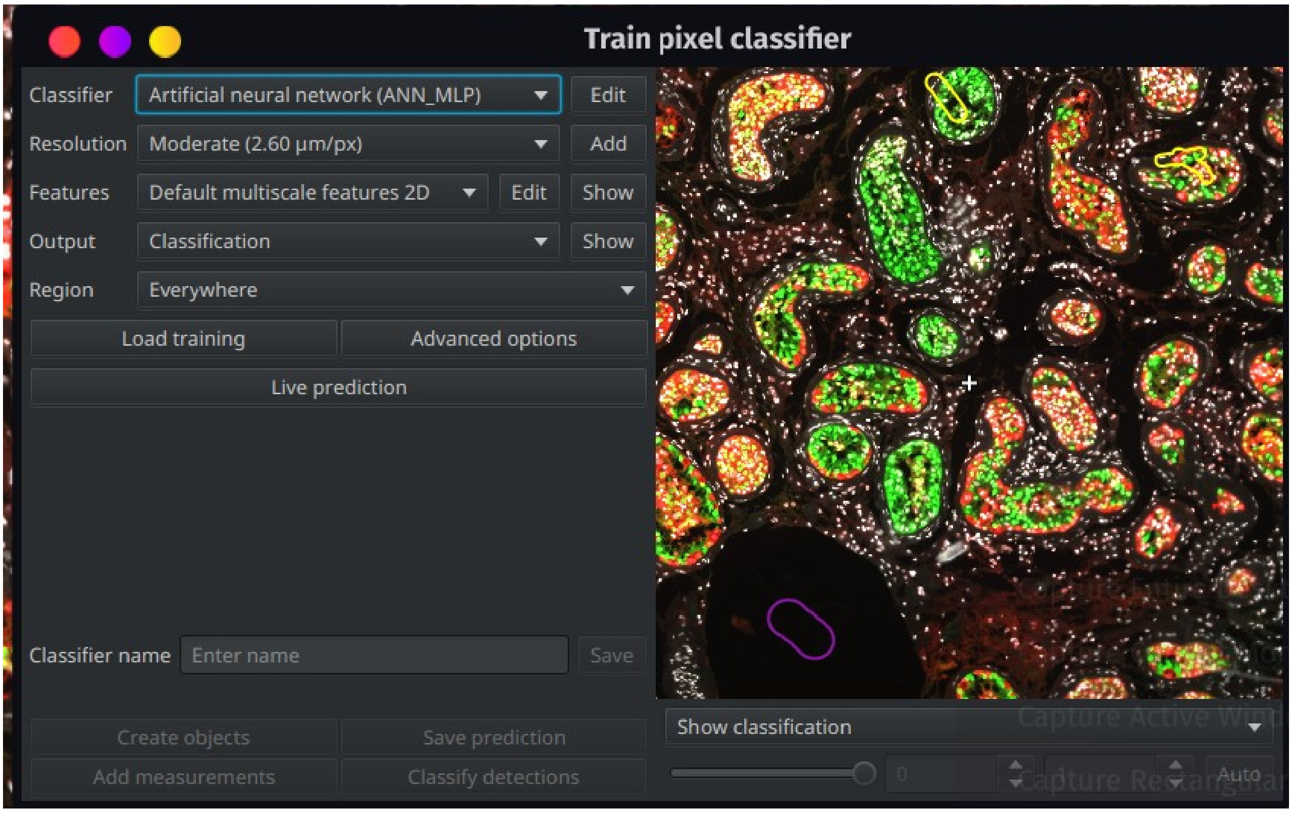

8 Click the load training button

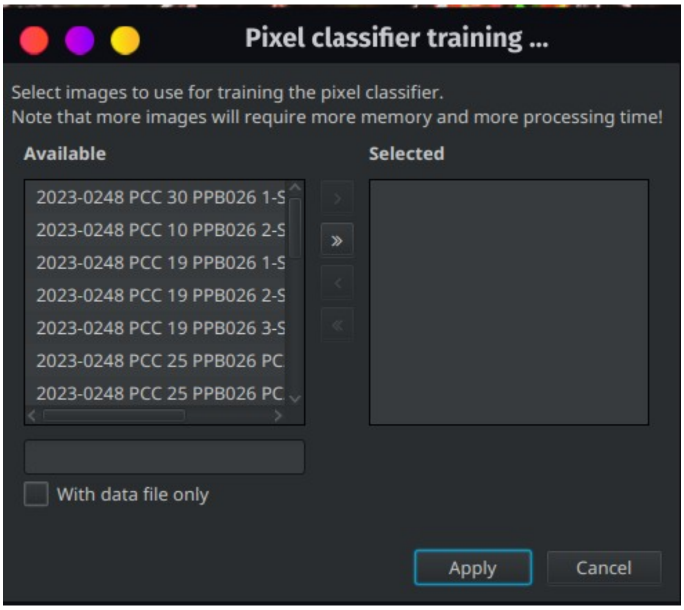

8.1 Move the **<u>only</u>** the selected images you have annotated to the right and click apply

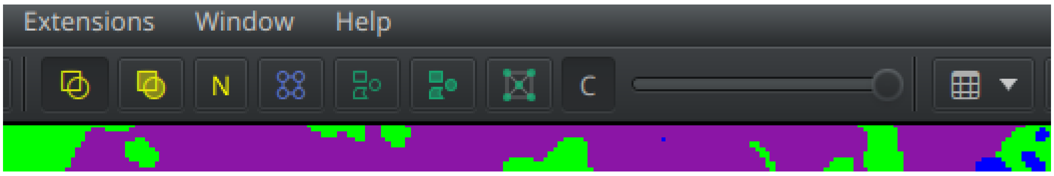

9 In the top menu, click the C button, then click 'live prediction' in the classifier training window.

9.1 This will give you a live preview of how the pixel classification is working based on the open image.

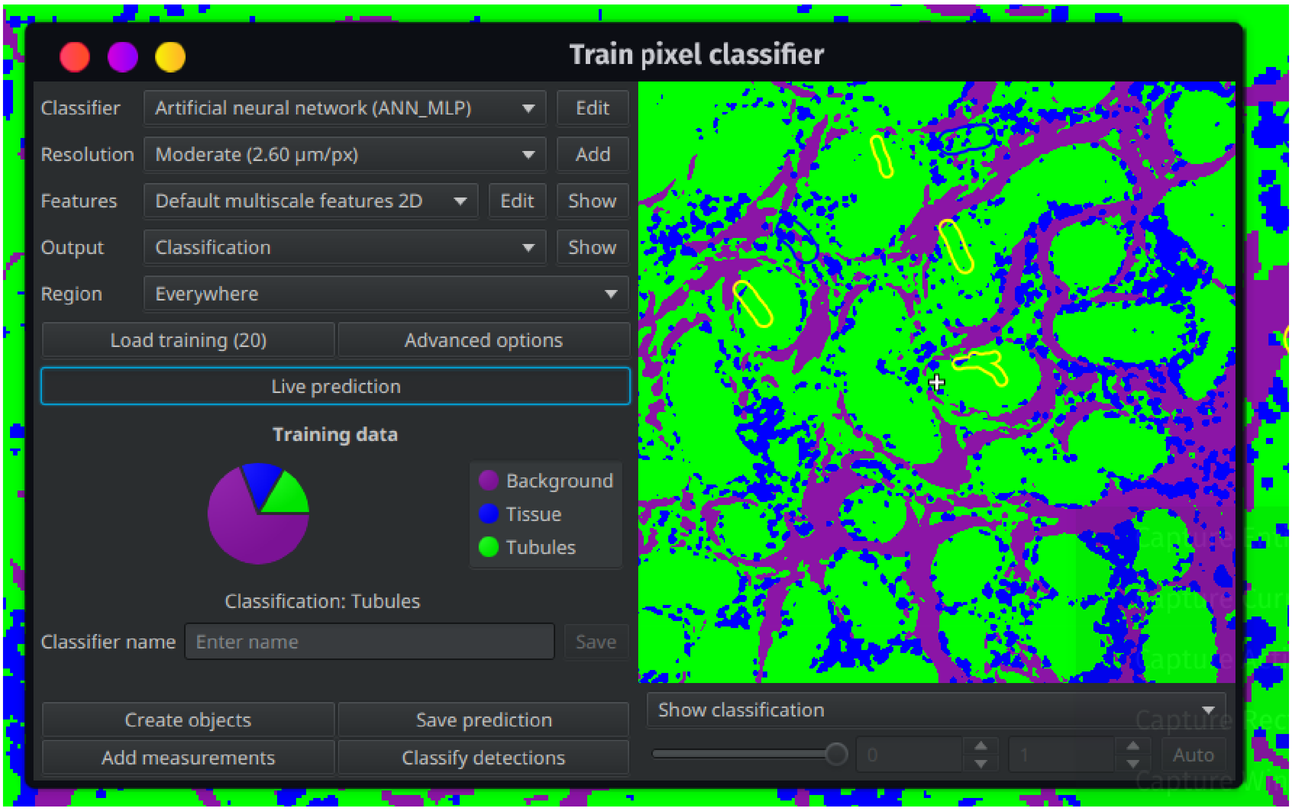

10 Depending on the results you are looking for it may be necessary to adjust the 'Features' by clicking the edit button.

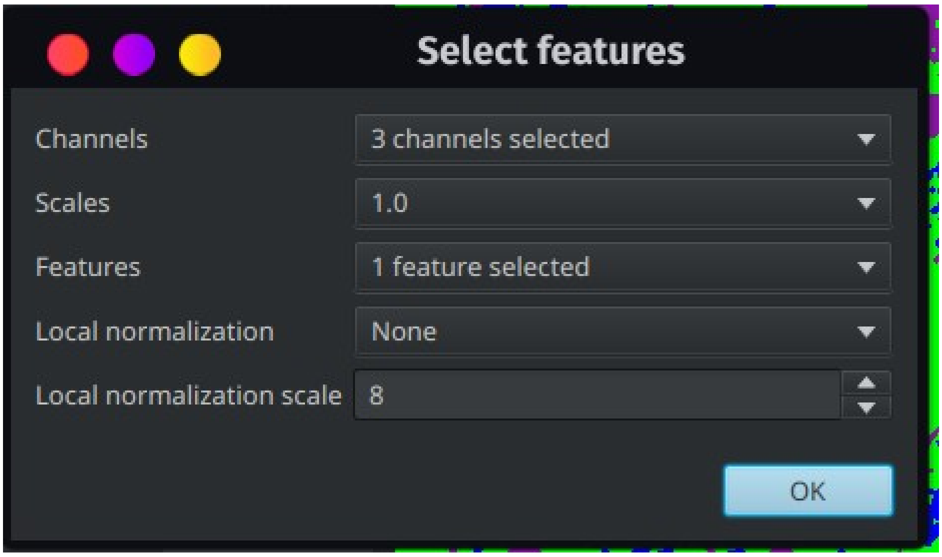

10.1 Select the channels you wish to train the classification on (potentially this could be all available channels).

10.2 Next select the features drop down and select the required features. This can depend on exactly what you are trying to segment. A good starting point is: Gaussian filter, Gradient magnitude, Structure tensor coherence and hessian determinant.

The usefulness of features

Feature	Purpose
Gaussian filter	General-purpose (color & intensity)
Laplacian of Gaussian	Blobby things, some edges
Weighted deviation	Textured vs. smooth areas
Gradient magnitude	Edges
Structure tensor eigenvalues	Long, stringy things
Structure tensor coherence	'Oriented' regions (e.g. aligned cells, fibers)
Hessian determinant	Blobby things (more specific than Laplacian)
Hessian eigenvalues	Long, stringy things

10.3 Then click OK to update the classifier.

11 Look at the live update and determine if the classification has worked based on the expected.

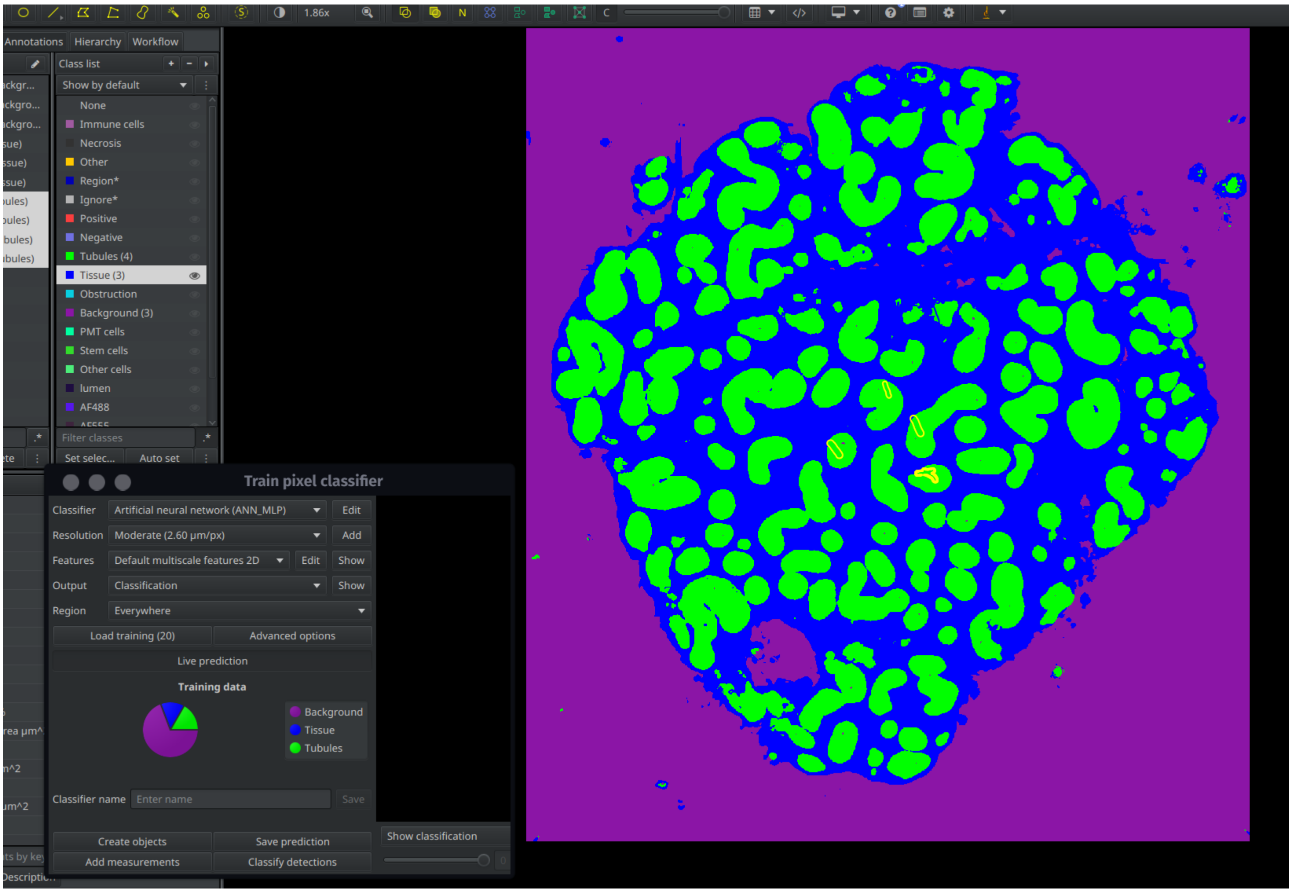

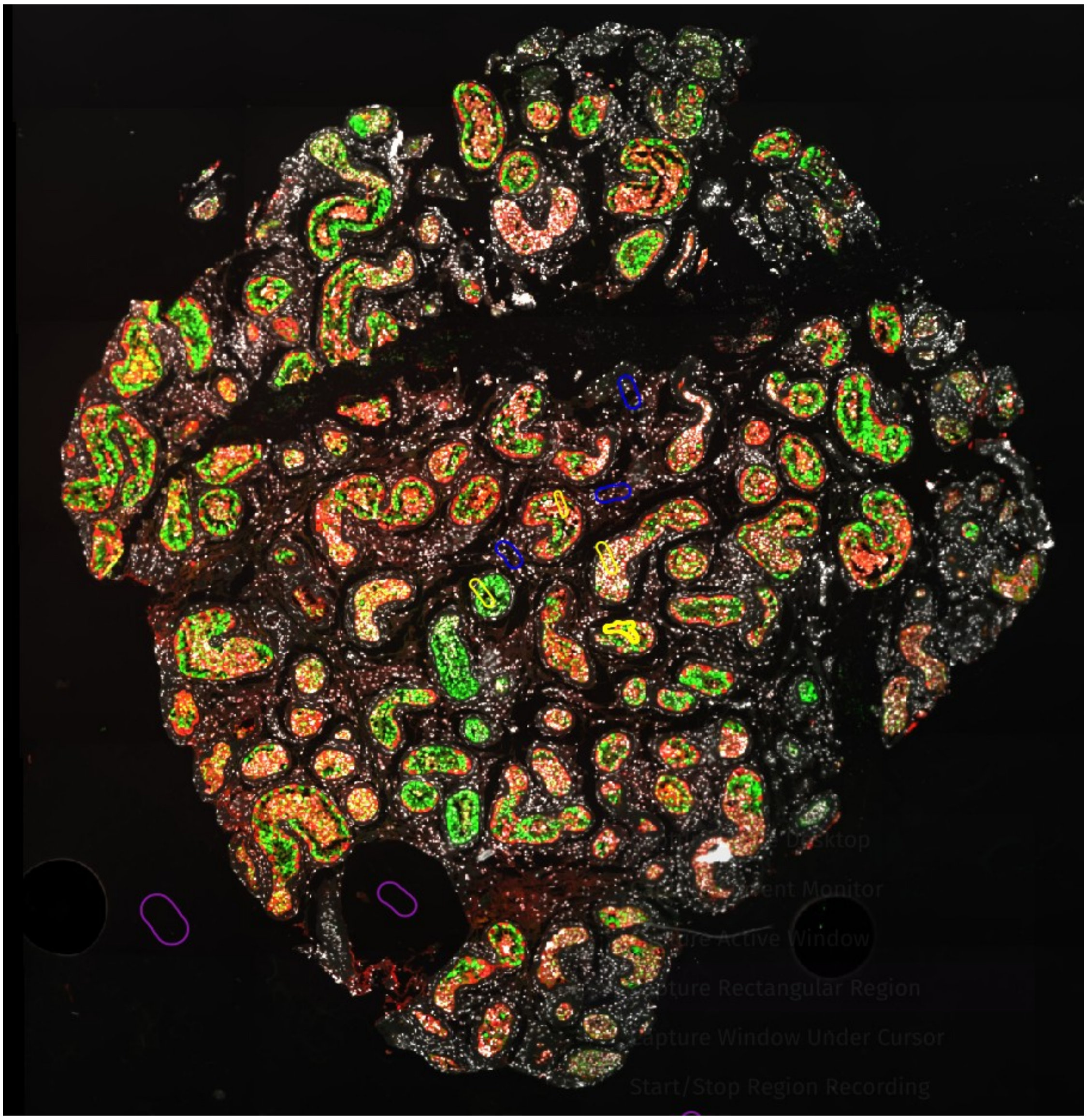

12 Once you are happy with the results save the classifier.

13 If you want to use the new classifier open the ‘Tubule’ script by going to automate → project scripts and open the tubule script.

13.1 In the script editor go to line 5 and change the classifier name to the one you just created. In this example the new classifier is called ‘Tubule’

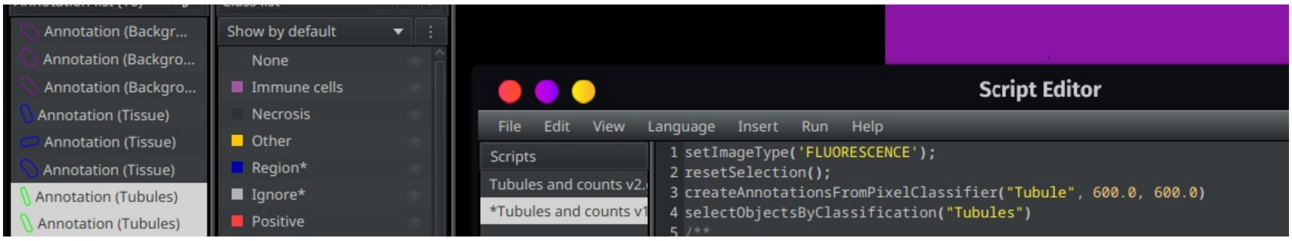

In addition change the region you want to count cells in, line 8 to any of the regions you have trained your classifier on. This is anything on the left. E.g Tissue, background or Tubules in this instance.

Creating an object classification system

When analysing images from different experiments or potentially when different people have performed the immunos, variation in the intensities of fluorescent staining is likely. Therefore the preconfigured classification system is unlikely to work for every experiment.

To generate an new classification system we need an image that has **<u>cells segmented in your ROI</u>**s:

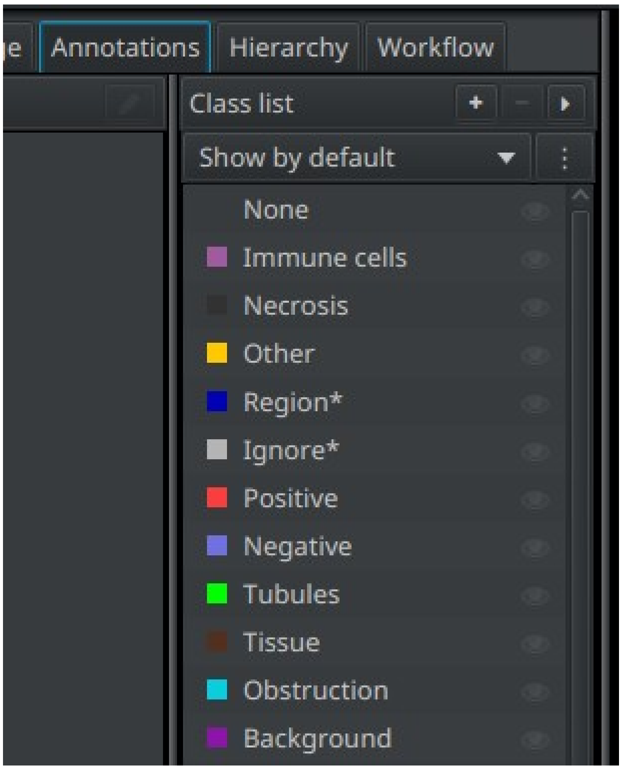

1 First add the cell classification you need to the class list by clicking the plus button and adding a suitable name. In this example we will use the fluorophores AF488 and AF647 but this could also be the type of marker or cell such as SOX9 and MAGE-A4.

2 Select a colour that contrasts to the fluorescent signal.

3 Next open the singe measurement classification system by going to Classify → object classification → create single measurement classifier.

3.1 This will open the following dialogue box

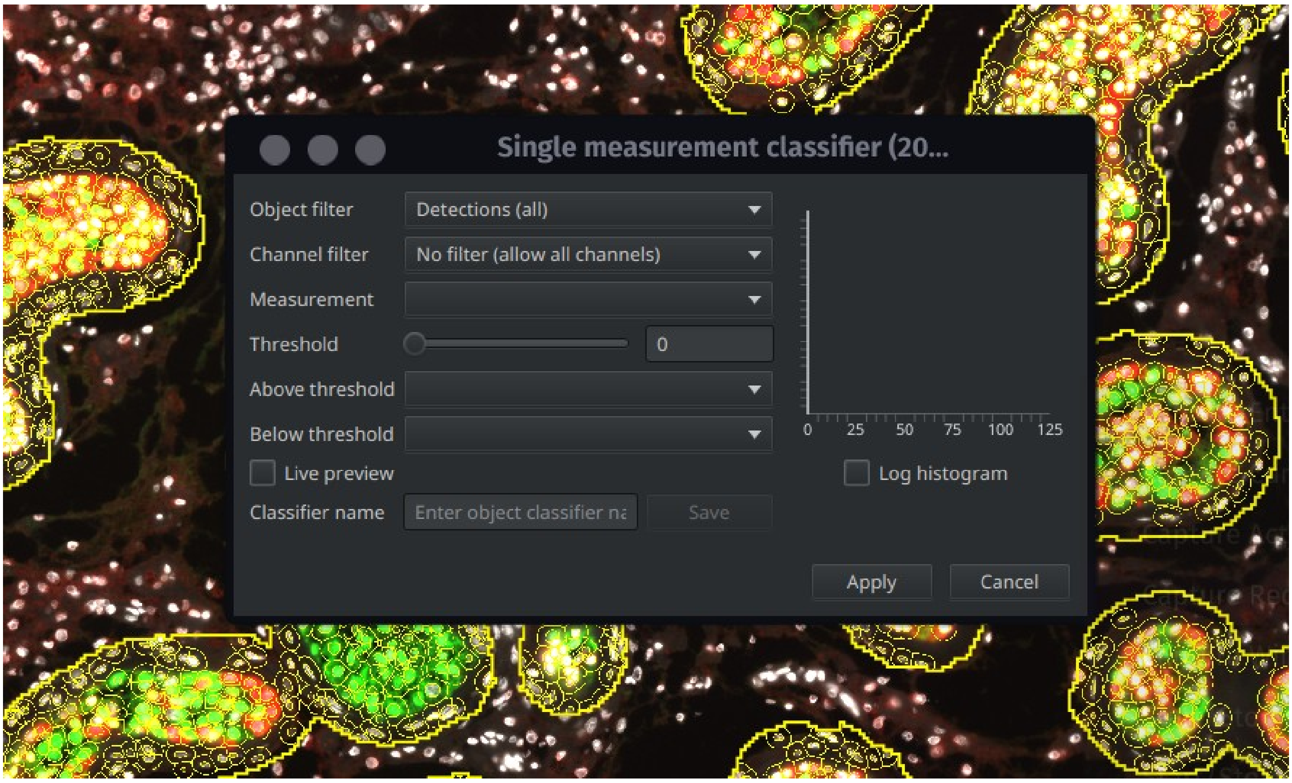

We will now adjust the setting to classify cells for SOX9 according to fluorescent intensity in the nucleus of AF488.

3.2 Currently all of the segmented cells in the tubules are unclassified (yellow outline).

3.3 We will select the 'object filter' to 'cells'. 'Channel filter' to 'AF488'. 'Measurement' to 'nucleus: AF488 mean'.

3.3.1 At this point the software will usually fill out the threshold value – This is usually a very good starting point so do not adjust it yet!

3.4 If not automatically selected, the 'above threshold' should be set to 'AF488' (this is the class we set earlier in this section).

3.5 Finally we will click live preview to see how the performance of object classification is working.

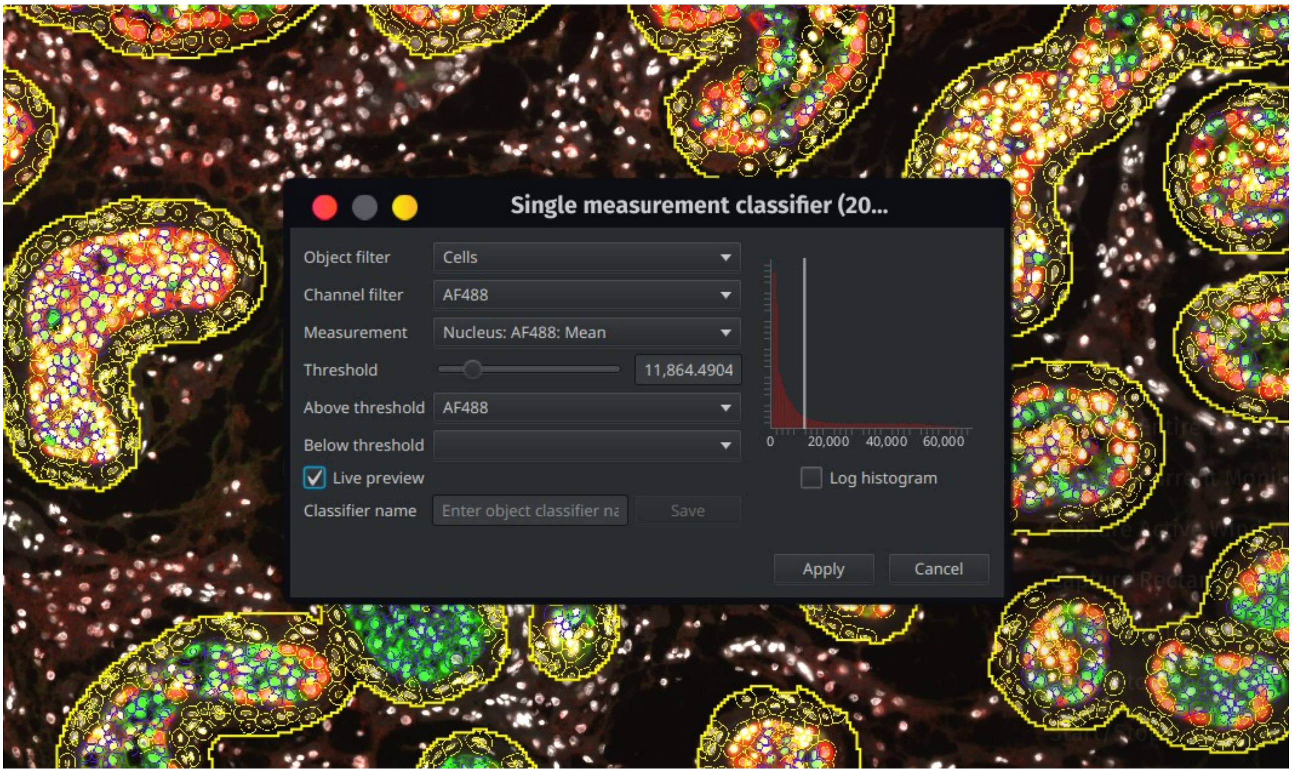

4 We can see that the outline of the green fluorescent cells are now blue indicating the classifier is working correctly.

5 Check the settings for your classifier of several images such as antibody controls, vehicle and treatment groups to ensure the classifier is working correctly.

6 Once you are happy with the performance save the classifier with an appropriate name. In this example we used ‘SOX9-AF488’.

7 Repeat this for all cell types you need. In this example we have produced two classifiers: ‘SOX9-AF488’ and ‘MAGE-A4-AF647’.

8 Once you have generated at least two single measurement classifiers we will combine this into a composite classifier.

9 Go to classify → object classification → create composite classifier

9.1 The following dialogue box will appear.

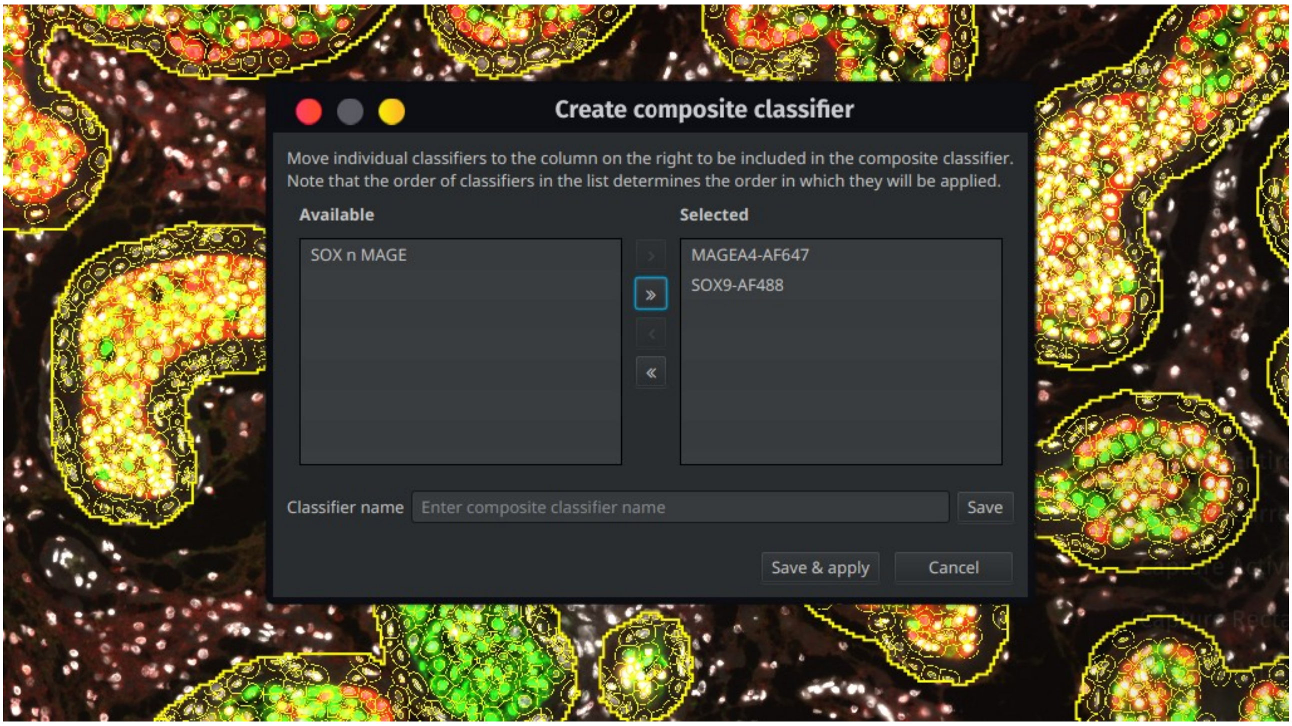

9.2 Move the single measurement classifiers we just created to the right. In this example we have two classifier ‘SOX9-AF488’ and ‘MAGE-A4-AF647’ that we will combine into a new classifier called ‘SOX n MAGE’ (which has all ready been created).

9.3 Enter a name and click save and apply.

10 We will then be able to classify images using multiple threshold or which ever detection settings you have chosen. We have created a very simple classification system of two fluorescent markers but this could be much more complex.

11 Review the results and if you are happy update the script.

11.1 Go to automate → project scripts and select tubule

11.2 Go to line 58 and change the name to the classifier you want to use in the script. In this example we are using the ‘Sox n MAGE’

Using the template script for image analysis

Please see the previous section Analysing your first project from the QuPath template: Segmentation of tubules and quantifying total cell number, SOX9 and MAGE-A4 cells in tubules for examples on how to use a configured script.

There is a default ‘Tubule’ script included in the template folder. This can either be run as is, or using the rest of this guide you can customise the segmentation and classification.

1 Once you are happy with the script configuration. Click run to run the script on a single image. This typically take anywhere from a few seconds to 60 depending on the size of the image and number of nuclei.

2 Once it has completed review the results and run on a couple of suitable test images such as antibody controls and vehicle/treatment groups to ensure the results are consistent.

3 Once you are happy, you can then click the ellipses and run for project. This will analyse all the images in your project with the same classification and segmentation setup.

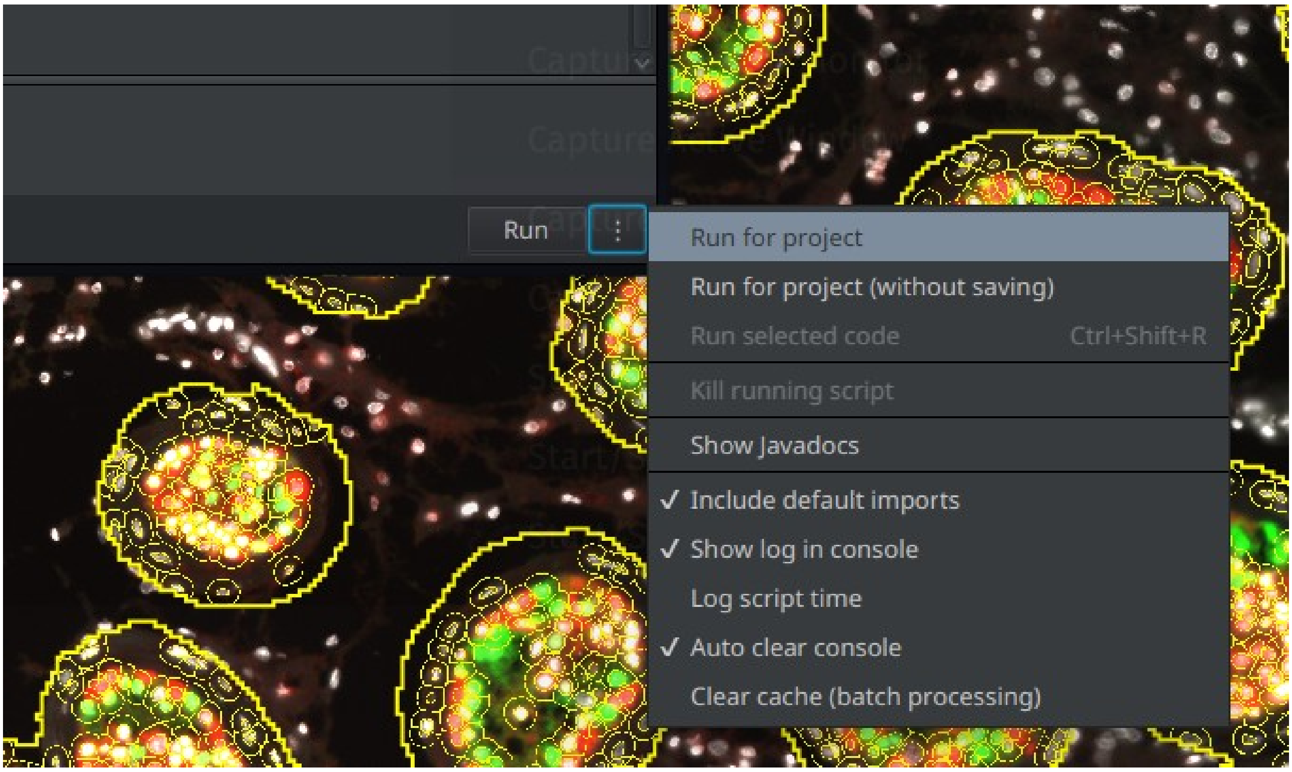

Editing object classifier threshold settings

In the event that you need to simply update the threshold vale of the object classification rather then recreating each component from scratch.

1 This can be done by modifying an existing classifier by opening the project folder → classifiers →object_classifiers and opening the corresponding .json file in your text editor.

```json
{
  "object_classifier_type": "SimpleClassifier",
  "function": {
    "classifier_fun": "ClassifyByMeasurementFunction",
    "measurement": "AF647: Nucleus: Mean",
    "pathClassEquals": {
      "name": "AF647",
      "color": [
        127,
        204,
        52
      ]
    },
    "pathClassAbove": {
      "name": "AF647",
      "color": [
        127,
        204,
        52
      ]
    },
    "threshold": 41210.7933180133
  },
```


2. Here you will find a section of the code relating to the threshold value for the AF647 classier which currently has a value of 41210.79….. you can modify this number and save.
3. The updated classifier can then be used without further modification.
